# Acupuncture for Migraine Without Aura and Connection-Based Efficacy Prediction: A Randomized Clinical Trial

**DOI:** 10.1001/jamanetworkopen.2025.55454

**Published:** 2026-01-27

**Authors:** Xinyu Zhang, Qiuyi Chen, Yuhan Liu, Jingyi Li, Limin Nie, Quan Miao, Feiyu Fu, Tianli Lyu, Zhongjian Tan, Yazhuo Kong, Bin Li, Lu Liu

**Affiliations:** 1 Department of Acupuncture and Moxibustion, Beijing Hospital of Traditional Chinese Medicine, Capital Medical University, Beijing Key Laboratory of Acupuncture Neuromodulation, Beijing, China; 2 School of Electronic and Information Engineering, Beijing Jiaotong University, Beijing, China; 3 Department of Radiology, Dong Zhimen Hospital Beijing University of Chinese Medicine, Beijing, China; 4 Department of Psychology, University of Chinese Academy of Sciences, Beijing, China

## Abstract

**Importance:**

Connectome-based predictive modeling (CPM) uses a data-driven whole-brain framework to identify connectivity patterns predicting clinical outcomes. However, CPM’s application to acupuncture in migraine without aura (MWOA) remains novel.

**Objective:**

To evaluate the clinical efficacy of real acupuncture and sham acupuncture in MWOA, and to identify acupuncture-response brain connectivity patterns using CPM analysis of baseline functional magnetic resonance imaging (fMRI) data.

**Design, Setting, and Participants:**

This single-blinded randomized clinical trial was conducted from June 2021 to June 2023 at Beijing Hospital of Traditional Chinese Medicine in China. It enrolled participants aged 18 to 65 years who met the MWOA criteria of the *International Classification of Headache Disorders, 3rd edition*. Eligible participants were randomly assigned to receive real acupuncture or sham acupuncture, and underwent baseline clinical assessments and baseline fMRI scans. Data analyses were performed between October 2024 and March 2025 following intention-to-treat and per-protocol principles.

**Interventions:**

Both treatment groups received 12 sessions of 30-minute acupuncture over 4 weeks. Real acupuncture involved 8 acupoints with deqi sensation, while sham acupuncture used sham acupoints without deqi.

**Main Outcomes and Measures:**

Primary outcome was the change from baseline in monthly migraine days (MMDs) during weeks 1 to 4. Secondary outcomes included 50% or greater reduction in MMDs and change from baseline in monthly headache days (MHDs), acute medication use days, pain score (visual analog scale [VAS] score range: 0 [indicating no pain] to 10 [indicating severe pain]), disability score (6-item Headache Impact Test [HIT-6] score range: 36 to 78, with the higher scores indicating severe headache effect), and quality-of-life score (Migraine-Specific Quality of Life Questionnaire [MSQ] score range: 0 to 100, with the higher scores indicating superior quality of life) during 4 weeks. Neuroimaging analyses used fMRI data to predict outcome changes via CPM.

**Results:**

Among the 120 participants (mean [SD] age, 36.8 [10.0] years; 95 females [79.2%]), 60 were randomly assigned to real acupuncture and 60 to sham acupuncture. The change from baseline in MMDs significantly improved for the real acupuncture group compared with the sham acupuncture group (median difference, −1.0; 95% CI, −2.0 to 0; *P* = .02). Significant differences were also observed in MHDs (median difference, −1.0; 95% CI, −2.0 to 0; *P* = .01), acute medication use days (median difference, −1.0; 95% CI, −2.0 to 0; *P* = .02), VAS score (median difference, −1.0; 95% CI, −1.0 to 0; *P* = .02), HIT-6 score (mean difference, −2.9; 95% CI, −5.4 to −0.5; *P* = .02), and MSQ score (Role Function-Restrictive domain: median difference, 8.6; 95% CI, 3.7-14.3; *P* < .001; Role Function-Preventive domain: median difference, 5.0; 95% CI, 0-10.0; *P* = .02; Emotional Function domain: median difference, 6.7; 95% CI, 0-13.3; *P* = .001). CPM revealed distinct neural signatures: negative connectivity predicted VAS score reduction (*r* = 0.23, *P* = .04) and positive connectivity predicted HIT-6 score improvement (*r* = 0.29, *P* = .02). Feature selection identified robust networks (12 connections for VAS score; 120 for HIT-6 score), in which default mode network and subcortical-cerebellum (DMN-SC) hypoconnectivity and SC-motor hyperconnectivity were identified as key connectivity patterns in predictive models of VAS and HIT-6 scores, respectively.

**Conclusions and Relevance:**

This trial demonstrated acupuncture’s efficacy for MWOA pain relief and functional improvement. CPM identified DMN-SC hypoconnectivity as predicting pain relief and SC-motor hyperconnectivity as predicting reduced disability, providing a personalized treatment framework.

**Trial Registration:**

Chinese Clinical Trial Registry Identifier: ChiCTR2100044251

## Key Points

**Question:**

Is acupuncture effective for migraine without aura, and can its efficacy be predicted from brain connectome data using machine learning?

**Findings:**

In this randomized clinical trial involving 120 participants with migraine without aura, acupuncture was effective in pain relief and functional improvement, and whole-brain resting-state functional connectivity can predict treatment outcomes.

**Meaning:**

The findings highlight changes in brain functional connectivity during acupuncture, showing the brain connectome’s potential for personalized therapy.

## Teaser

This randomized clinical trial assesses the efficacy of acupuncture for pain and disability reduction using a whole-brain predictive model to identify acupuncture’s therapeutic outcome for migraine without aura.

## Introduction

Migraine without aura (MWOA) is a prevalent and debilitating disorder impairing quality of life.^1^ Nonspecific medications are recommended for mild to moderate acute MWOA, while specific medications are indicated for severe attacks or nonsteroidal anti-inflammatory drug treatment failures. However, nearly 30% of patients show inadequate response to nonsteroidal anti-inflammatory drugs or triptans.^2,3,4^ Even with preventive agents, the proportion of patients who both respond to and tolerate treatment remains less than 60%.^3^ Acupuncture is an evidence-based alternative with comparable efficacy to drugs but a superior safety profile.^5,6,7^ Neuroimaging studies suggest that acupuncture modulates pain-processing networks such as the default mode network (DMN) and primary somatosensory cortex.^8,9,10^ However, predicting acupuncture response based on functional connectome in migraine remains unexamined.

Neuroimaging biomarkers for treatment prediction require careful consideration. Migraine-related pain involves whole-brain functional connectivity alterations,^11,12,13,14^ particularly in the DMN, subcortical-cerebellum (SC), and motor networks. These patterns can act as diagnostic markers and predict treatment responsiveness.^15,16,17^ We hypothesized that connectome analysis, by overcoming regional approach limitations, can better predict acupuncture outcomes in migraine.

Connectome-based predictive modeling (CPM) offers clear advantages over conventional neuroimaging for studying acupuncture’s neuromodulatory effects. Unlike hypothesis-driven seed-based analyses^18^ or region-specific measures, such as the amplitude of low-frequency fluctuation,^19^ CPM uses a data-driven whole-brain framework to identify connectivity patterns predicting clinical outcomes.^20^ It extracts predictive features without predefined assumptions, capturing distributed network interactions often missed by regional analyses, and generates individualized predictions from baseline connectivity. While CPM has predicted depression treatment response,^21^ its application to acupuncture in migraine, distinguishing pain relief from disability improvement, remains novel. The present study applies CPM to decode neural signatures underlying acupuncture’s therapeutic mechanisms in migraine.

We aimed to evaluate the clinical efficacy of real acupuncture and sham acupuncture in MWOA, with CPM analysis of baseline functional magnetic resonance imaging data and to identify acupuncture-response brain connectivity patterns. This approach offers novel insights into acupuncture’s neuromodulatory mechanisms and supports personalized migraine treatment.

## Methods

### Trial Design and Participants

The single-blinded randomized clinical trial (RCT) was conducted from June 2021 to June 2023 at Beijing Hospital of Traditional Chinese Medicine. It included a screening (lasting 3-14 days), a 4-week baseline, and a 4-week treatment period. All participants provided written informed consent. The Research Ethical Committee of Beijing Hospital of Traditional Chinese Medicine, Capital Medical University approved the RCT, which adhered to Good Clinical Practice and the Declaration of Helsinki.^22^ We followed the Consolidated Standards of Reporting Trials (CONSORT) reporting guideline. The statistical analysis plan and study protocol are available in Supplement 1 and Supplement 2, respectively.

The eligibility criteria included a diagnosis of MWOA according to the *International Classification of Headache Disorders 3rd edition* (*ICHD-3*),^23^ age 18 to 65 years, 2 or more migraine attacks in the past 4 weeks, and 1 year or longer migraine history. Main exclusion criteria were specific headache types per the *ICHD-3*; use of migraine preventive treatment, acupuncture, or migraine devices within the past 3 months; a history of medication overuse–related headache; serious medical conditions, pregnancy, lactation, or inadequate contraception; and contraindications to magnetic resonance imaging (MRI). The complete inclusion and exclusion criteria are provided in eMethods 1 in Supplement 3.

### Randomization and Masking 

**Figure 1.**
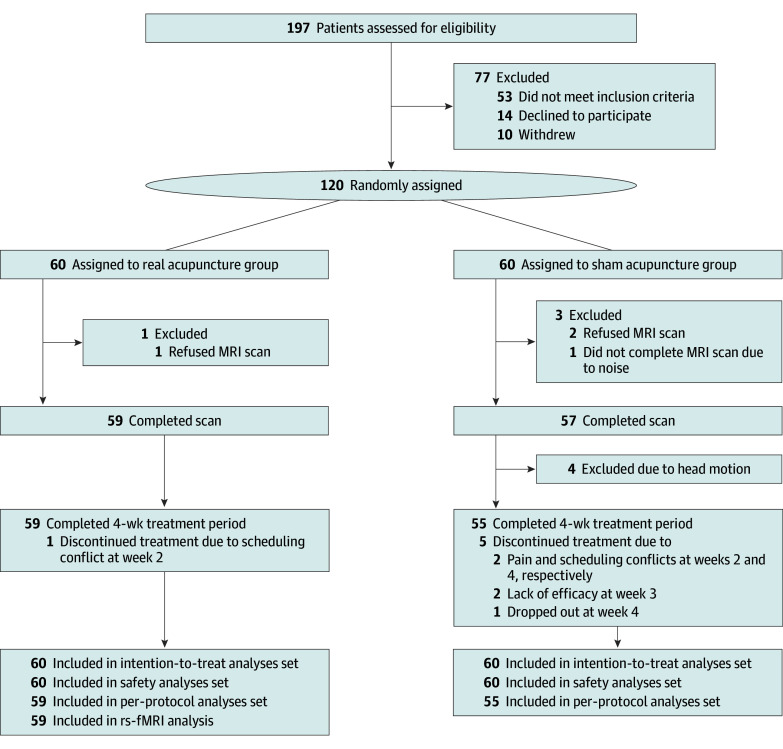
Trial Flow Diagram MRI indicates magnetic resonance imaging; rs-fMRI, resting-state functional MRI.

After the baseline period, eligible participants were randomly assigned 1:1 to receive real acupuncture or sham acupuncture (Figure 1) via an interactive web-based system (Beijing LNKMED Tech Co Ltd). A computer-generated code with fixed block size 4, performed by an independent statistician, ensured randomization. Participants, outcome assessors, and statistical analysts were blinded to the group assignments.

### Intervention 

Participants in the real acupuncture group received twelve 30-minute acupuncture sessions over 4 weeks (3 sessions per week, ideally every other weekday). Acupoint prescriptions followed those used in prior studies.^24,25^ Acupuncturists had at least 5 years of clinical experience. The 8 acupoints selected were Baihui (GV20), Fengfu (GV16), bilateral Fengchi (GB20), bilateral Taiyang (EX-HN5), and bilateral Hegu (LI4). Locations and procedures are shown in eFigure 1 and eTables 1 and 2 in Supplement 3. Single-use Hwato needles (25-40 mm length; 0.25-0.30 mm diameter) were used. Twirling, lifting, and thrusting aimed to elicit deqi (distention, numbness, soreness, or radiating sensations).

The sham acupuncture group received superficial needling at 8 sham acupoints (eFigure 2 and eTable 3 in Supplement 3), which were chosen based on prior work^26^ to minimize physiological effects and because they were not located on recognized meridians. Manipulation matched real acupuncture but omitted deqi.^27^ Treatments were delivered individually, with standardized instructions for maintaining consistency.

Participants recorded detailed migraine attack information (duration, frequency, pain intensity by visual analog scale [VAS] score, and acute medication use) in a headache diary.^28^ Neurologist-prescribed acute medications were permitted if they were the same used in the baseline. Further details on diary content are provided in the Study Protocol in Supplement 2.

### Outcomes

The primary outcome was the change from baseline in monthly migraine days (MMDs) during weeks 1 to 4. A migraine day was defined as a calendar day with a headache lasting 4 or more hours, meeting the *ICHD-3* criteria, or a day that acute migraine-specific medication (triptans or ergot derivatives) was used.^29,30^

Secondary outcomes included the proportions of participants achieving a 50% or greater reduction in MMDs and changes from baseline in monthly headache days (MHDs), acute medication use days, pain score (on the VAS; score range: 0 [indicating no pain] to 10 [indicating severe pain]), disability score (on the 6-item Headache Impact Test [HIT-6]; score range: 36-78, with the higher scores indicating severe headache effect), and quality-of-life score (on the Migraine-Specific Quality of Life Questionnaire [MSQ]; score range: 0-100, with the higher scores indicating superior quality of life) during weeks 1 to 4.^31,32,33,34,35^ All scales were validated in Chinese. Details of the scales are provided in eMethods 2 in Supplement 3.

Safety outcomes included adverse events (AEs) and serious adverse events (SAE). AEs were defined as those persisting from first treatment to study end. SAEs were defined as medical incidents resulting in death, life-threatening conditions, or other serious outcomes. A data monitoring committee oversaw the safety data.

Acupuncture expectancy was evaluated before treatment. Participant satisfaction and blinding via Bang Blinding Index (BBI)^36^ were assessed after treatment. Compliance was defined as completion of at least 10 of 12 sessions (≥80%).

### Neuroimaging Acquisition and Network Construction

MRI scans were performed at baseline (3.0 T Siemens Magnetom Verio; Siemens) at the MRI Center of Dong Zhimen Hospital, Beijing University of Chinese Medicine. Scanning occurred in the interictal phase at least 72 hours after a migraine attack. Acquisition parameters are provided in eMethods 3 in Supplement 3.

Image preprocessing is described in eMethods 4 in Supplement 3. Whole-brain functional connectivity was analyzed with BioImage Suite^37^ following previously reported methods.^38,39,40^ Network nodes were defined by the 268-node Shen atlas (eMethods 5 in Supplement 3), consistent with prior CPM work (eMethods 6 in Supplement 3).^21,41^ We computed the mean time course for each node and calculated node-by-node Pearson correlations. The resulting *r* values (correlation coefficients) were Fisher *z*–transformed to produce symmetric 268 × 268 connectivity matrices, with each element representing an edge strength.^20,40^

### Statistical Analysis 

#### Efficacy Analysis

Data analyses were performed between October 2024 and March 2025. Based on a previous study,^42^ the change from baseline in mean (SD) MMDs during 4 weeks was 2.2 (2.0) and 1.6 (3.0) in the real acupuncture and sham acupuncture groups, respectively. We calculated that 54 patients would provide 80% or more study power at a 2-sided α = .05. Accounting for a 10% dropout, we aimed for 60 patients per group.

Primary and secondary outcomes were analyzed using intention-to-treat and per-protocol sets. The intention-to-treat analysis included all randomized participants, while the per-protocol set comprised participants who met eligibility criteria and completed planned treatment without major deviations. The safety analysis set included participants receiving 1 or more study treatment.

Participants with at least 14 days of nonmissing diary data (≥50% of 28 days) had their data prorated to 28 days. Participants with fewer than 14 days of diary data were treated as having missing data. The statistical data were derived from 10 imputed datasets. The SD of the mean was adjusted based on the imputation variance estimates.

Variables were first assessed for distributional characteristics. For outcomes that departed from normality, comparisons between the real acupuncture and sham acupuncture groups were performed using the Wilcoxon rank-sum test (Mann-Whitney *U*), and the Hodges-Lehmann estimate was used as the point estimate of the median difference; the Hodges-Lehmann estimate and its 95% CI were derived using the Wilcoxon test. All tests were 2-sided with α = .05. Results are presented as medians and IQRs (25th-75th percentiles) for each group, with between-group differences given as the Hodges-Lehmann estimates and 95% CIs along with 2-sided *P* values. For outcomes that were normally distributed, comparisons were made with independent-sample, 2-tailed *t* tests. Results were reported as mean changes with SEs and 95% CIs, and between-group differences as mean differences with 95% CIs and 2-sided *P* values. The proportion of participants with 50% or greater reduction in MMDs was assessed using a logistic regression model. The AEs were summarized in terms of counts and percentages.

Blinding effectiveness was assessed through participant perceptions using the χ^2^ test and BBI, categorizing beliefs (real acupuncture, sham acupuncture, or unknown). All tests were 2-sided with a significance level of *P* < .05. Secondary end point analyses were not adjusted for multiple comparisons and, given the potential for type I error, should be interpreted as exploratory. Analyses used R, version 4.0.3 (R Core Team).

#### CPM of Clinical Outcome

In the primary analysis, we used CPM with custom MATLAB scripts (MathWorks Inc), building predictive models from pretreatment whole-brain functional connectivity matrices and behavioral data. CPM, which was selected for its ability to handle spatially distributed connectivity changes in MWOA and improve prediction precision, extracts predictive indicators. Protocols and scripts were obtained from Shen et al.^20^ eFigure 3 in Supplement 3 shows a schematic of CPM. In the secondary analysis, we compared selected connections with anatomical structures and canonical functional networks.

CPM comprised 3 stages: feature selection, model construction, and model evaluation. Edges with significant positive or negative Pearson correlations (*P* < .01) with behavioral data were identified. Summing Fisher *z*–normalized *r* values of selected positive and negative edges yielded 2 network strength measures per participant, which, along with data changes, served as model inputs. Leave-one-out cross-validation was used, training iteratively on all but 1 participant and predicting the left-out participant. Model accuracy between predicted and actual score changes was assessed by Spearman correlation.

Statistical significance was estimated via 5000 random permutations of changes with leave-one-out cross-validation, yielding a null distribution of correlation coefficients. Permutation *P* value was the proportion of permuted *r* values equal to or greater than the observed *r* values. An edge was deemed robust if selected in 50% or more of cross-validation iterations,^21^ balancing sensitivity and specificity for smaller samples and highlighting acupuncture-affected edges.

## Results

### Participant Characteristics

Between June 2021 and June 2023, 197 participants were screened and 77 were excluded. A total of 120 participants (mean [SD] age, 36.8 [10.0] years; 95 females [79.2%], 25 males [20.8%]) were randomly assigned to either the real acupuncture group (n = 60) or sham acupuncture group (n = 60). Six participants dropped out after randomization due to time conflicts or doubts about the treatment efficacy (Figure 1). Baseline demographic characteristics showed no between-group differences (eTables 4 and 5 in Supplement 3).

### Clinical Efficacy and Secondary Outcomes

After 4 weeks, changes in MMDs from baseline significantly improved in the real acupuncture group compared with the sham acupuncture group (median difference, −1.0; 95% CI, −2.0 to 0; *P* = .02) (Table). This finding supports our primary hypothesis of the specific physiological effects of real acupuncture. Analysis for the per-protocol dataset showed similar results (eTable 6 in Supplement 3).

**Table.** Primary and Secondary Clinical Efficacy Outcomes in the Intention-to-Treat Population During the Treatment Period

Outcome	Participants, median (IQR)		Median difference (95% CI)	*P* value
	Real acupuncture group (n = 60)	Sham acupuncture group (n = 60)		
Primary outcome				
Change from baseline in MMDs during wk 1 to 4	−3.0 (−5.0 to −2.0)	−3.0 (−4.0 to −1.0)	−1.0 (−2.0 to 0)	.02
Secondary outcomes				
≥50% Reduction in MMDs during wk 1 to 4, percentage (SE)	66.7 (6.1)	53.3 (6.5)	OR: 1.80 (0.84 to 3.66)	.14
Change from baseline in MHDs during wk 1 to 4	−4.0 (−6.0 to −2.8)	−3.0 (−4.0 to −1.0)	−1.0 (−2.0 to 0)	.01
Change from baseline in monthly acute medication use days during wk 1 to 4	−3.0 (−4.0 to −2.0)	−2.0 (−4.0 to −1.0)	−1.0 (−2.0 to 0)	.02
Change from baseline in VAS total score at wk 4^a^	−2.0 (−4.0 to −1.0)	−1.0 (−2.1 to −1.0)	−1.0 (−1.0 to 0)	.02
Change from baseline in HIT-6 total score at wk 4, mean (SE)^b^	−8.3 (0.8)	−5.4 (0.9)	Mean difference: −2.9 (−5.4 to −0.5)	.02
Change from baseline in MSQ RR score at wk 4^c^	20.0 (11.4 to 27.8)	8.6 (2.1 to 20.7)	8.6 (3.7 to 14.3)	<.001
Change from baseline in MSQ RP score at wk 4^c^	15.0 (5.0 to 25.0)	10.0 (0 to 20.0)	5.0 (0 to 10.0)	.02
Change from baseline in MSQ EF score at wk 4^c^	13.3 (6.7 to 20.0)	0 (0 to 15.0)	6.7 (0 to 13.3)	.001

Abbreviations: EF, Emotional Function; HIT-6, 6-item Headache Impact Test; MHD, monthly headache day; MMD, monthly migraine day; MSQ, Migraine-Specific Quality of Life Questionnaire; OR, odds ratio; RP, Role Function-Preventive; RR, Role Function-Restrictive; VAS, visual analog scale.

^a^ VAS score range: 0 (indicating no pain) to 10 (indicating severe pain).

^b^ HIT-6 score range: 36 to 78, with the higher scores indicating severe headache effect.

^c^ MSQ score range: 0 to 100, with the higher scores indicating superior quality of life.

#### Reduction in Migraine Severity

The real acupuncture group showed significant changes in MHDs (median difference, −1.0; 95% CI, −2.0 to 0; *P* = .01), VAS score (median difference, −1.0; 95% CI, −1.0 to 0; *P* = .02), and monthly acute medication use days (median difference, −1.0; 95% CI, −2.0 to 0; *P* = .02) (Table; eTable 6 in Supplement 3). However, no significant difference was observed for 50% or greater reduction in MMDs (OR, 1.80; 95% CI, 0.84-3.66; *P* = .14).

#### Disability and Quality of Life

After 4 weeks of treatment, the real acupuncture group compared with the sham group had significantly improved mean HIT-6 score (mean difference, −2.9; 95% CI, −5.4 to −0.5; *P* = .02). The real acupuncture group also had improved MSQ scores (Role Function-Restrictive domain: median difference, 8.6; 95% CI, 3.7-14.3; *P* < .001; Role Function-Preventive domain: median difference, 5.0; 95% CI, 0-10.0; *P* = .02; Emotional Function domain: median difference, 6.7; 95% CI, 0-13.3; *P* = .001) compared with the sham acupuncture group (Table).

#### Satisfaction, Expectation, Compliance, and Blinding

At week 4, the real acupuncture group reported higher satisfaction compared with the sham acupuncture group (58 [96.7%] vs 44 [73.3%]; *P* = .01) (eTable 7 in Supplement 3). No baseline differences in improvement expectation were observed (eTable 8 in Supplement 3). Participants who received real acupuncture showed better but nonsignificant compliance than those who received sham acupuncture (eTable 9 in Supplement 3). There were no between-group response differences. BBI results between the sham acupuncture and real acupuncture groups indicated successful blinding (BBI = −0.40 [95% CI, −0.59 to −0.29] vs 0.60 [95% CI, 0.35-0.85]) (eTable 10 in Supplement 3).

#### Safety Analyses

Of 120 patients, 5 (4.2%) in the real acupuncture group reported AEs (subcutaneous hematoma, needling pain, and numbness). The same number of AEs occurred in the sham acupuncture group, with no significant difference (eTable 11 in Supplement 3). All AEs were mild and self-limiting, and no SAEs were reported.

### Connectome-Based Clinical Outcome Predictions

**Figure 2.**
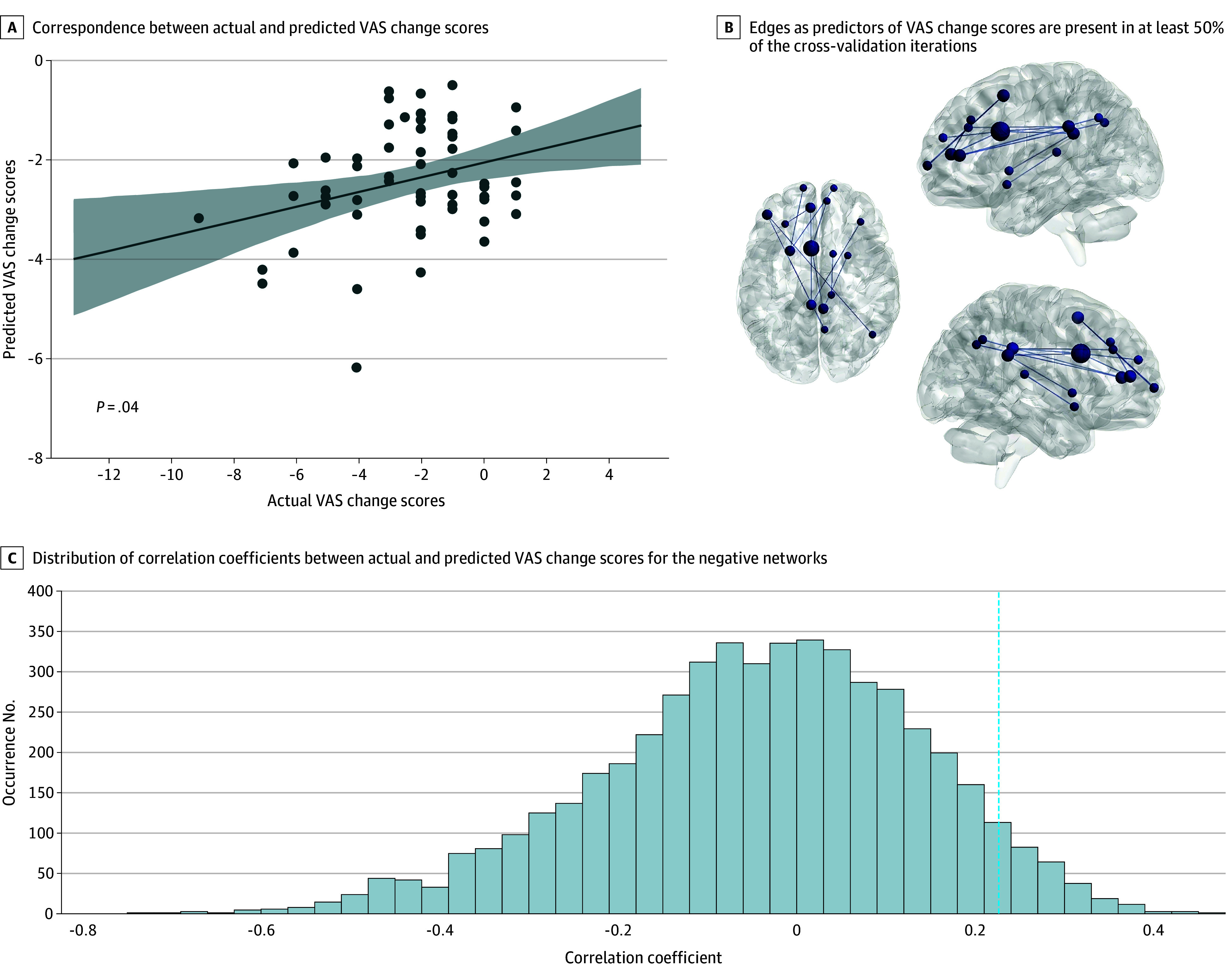
Connectome-Based Predictive Modeling for Visual Analog Scale (VAS) Score Changes A, Straight line represents the linear regression line showing the association between actual and predicted VAS score changes. Shaded area represents the 95% CI around the regression line. B, The thickness of the edges indicates the occurrence frequency. Circles represent brain nodes, and straight lines represent brain edges. C, Dashed line represents the position of the observed correlation coefficient for each negative network.

**Figure 3.**
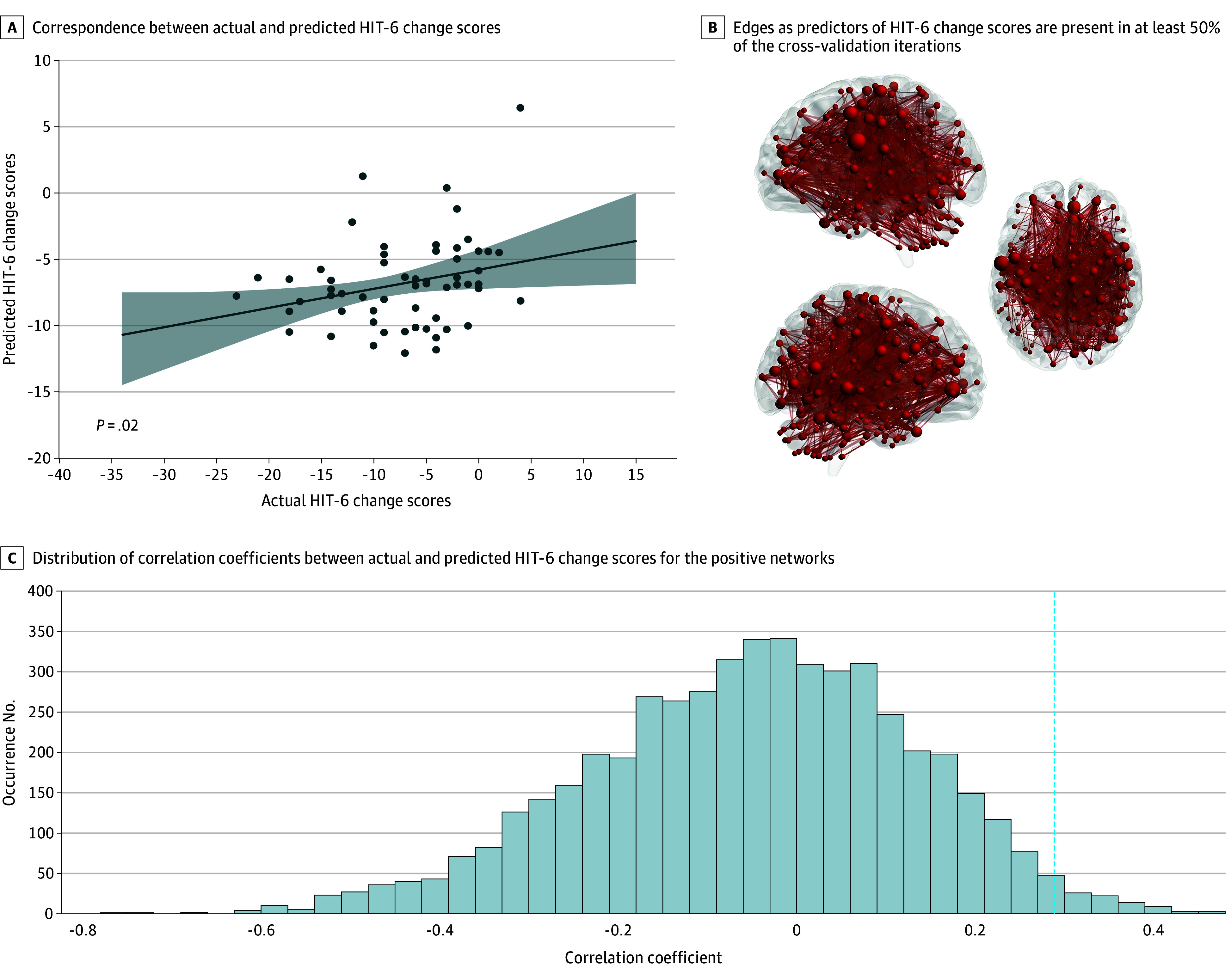
Connectome-Based Predictive Modeling for 6-Item Headache Impact Test (HIT-6) Score Changes A, Straight line represents the linear regression line showing the association between actual and predicted HIT-6 score changes. Shaded area represents the 95% CI around the regression line. B, The thickness of the edges indicates the occurrence frequency. Circles represent brain nodes, and straight lines represent brain edges. C, Dashed line represents the position of the observed correlation coefficient for each positive network.

Baseline negative functional connectivity predicted VAS score reduction (*r* = 0.23, *P* = .04; permutation *P* < .001) (Figure 2). Positive connectivity better predicted HIT-6 score improvement (*r* = 0.29, *P* = .02; permutation *P* < .001) (Figure 3). Among connections selected in 50% or greater cross-validation iterations, 12 belonged to the VAS score–related negative network (Figure 2) and 120 to the HIT-6 score–related positive network (Figure 3). These robust connections were widely distributed, showing no preference for particular known functional networks.

However, this predictive power did not extend to all clinical outcomes. CPM for MMDs, MHDs, acute medication use days, and MSQ scores did not yield significant predictive performance (eAppendix in Supplement 3).

### Nodal Distribution and Functional Overlap With Canonical Neural Networks

**Figure 4.**
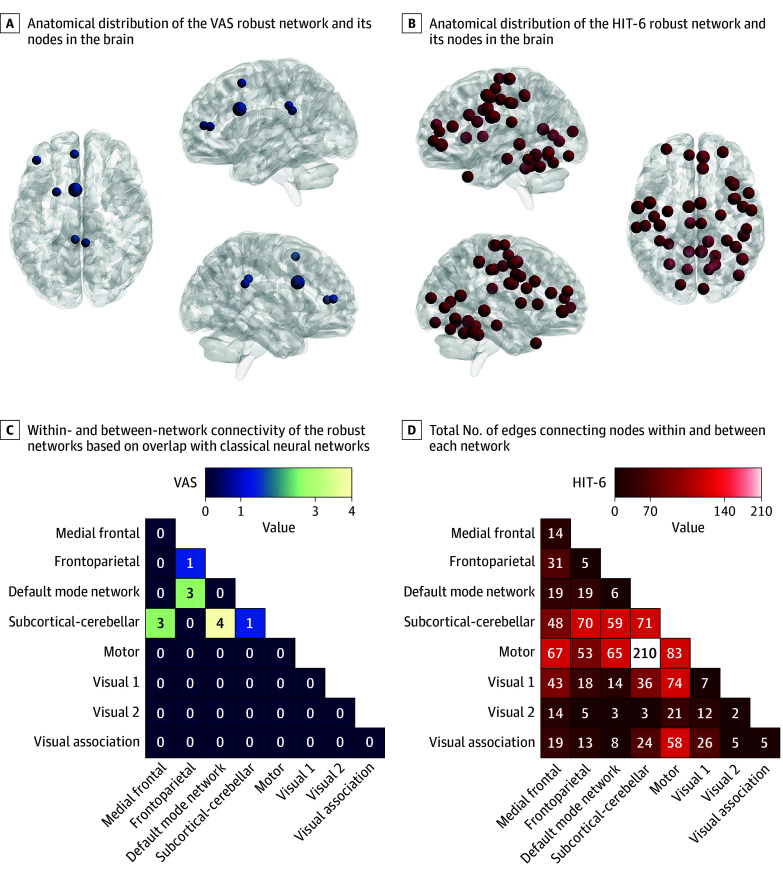
Anatomical Distribution of Robust Predictive Networks and Nodes Overlaid With Canonical Brain Networks A and B, Circles represent brain nodes. The size of the circles indicates their significance within the respective robust network; larger nodes denote greater importance. C and D, Classical neural networks include the visual analog scale (VAS) score–predictive robust negative network and the 6-item Headache Impact Test (HIT-6) score–predictive robust positive network. Brighter colors indicate higher edges. Numbers in boxes indicate the number of edges within and between networks.

Figure 4 summarizes the anatomical distribution of robust network nodes for VAS and HIT-6 scores and their overlap with canonical neural networks, revealing potential mechanisms of acupuncture treatment for MWOA. Important VAS score–related nodes localized mainly to the limbic system, prefrontal cortex, putamen, and occipital lobe. Important HIT-6 score–related nodes were primarily found in the caudate nucleus, hippocampus, thalamus, motor cortex, prefrontal cortex, and cerebellum. Regarding network overlap, the VAS score–predictive robust negative network showed greater overlap with DMN-SC network connections. The HIT-6 score–predictive robust positive network was more prominently involved in SC-motor network connections.

## Discussion

This RCT with CPM identified baseline connectivity biomarkers predicting acupuncture-related improvements in pain and disability in patients with MWOA. Our findings revealed distinct functional brain patterns linked to improved treatment responses. Refined, representative internetwork connectome features showed strong predictive value for clinical improvements following acupuncture. Negative network connections (eg, DMN-SC) were predictive of changes in VAS score, while positive network connections (eg, SC-motor) were predictive of changes in HIT-6 score.

We predicted acupuncture efficacy using whole-brain functional connectome analysis via CPM. CPM, a supervised approach, objectively extracts predictive features without predefined assumptions, capturing distributed network interactions often missed by regional analyses. Brain connectivity strength reflects interregional collaboration, information processing, and functional integration, influenced by pathological and physiological factors and noise.^43,44^ To reduce false-positive results, we used a 50% feature selection threshold and conducted an overlap analysis with established functional brain networks. CPM identified positive and negative networks associated with acupuncture effects, mapped onto 7 canonical functional brain networks. This pattern may also indicate MWOA symptom severity and prognosis. While CPM does not eliminate all false-positive results, it could be refined in future studies.

Our findings suggest that baseline functional connectivity indicates a predisposition for treatment responsiveness, reflecting a covariation between connectivity strength and therapeutic efficacy rather than a direct causal association. Migraine pain reduction and functional restoration rely on brain remodeling: neuroplastic adjustments, pain-modulatory network reintegration, altered cortical excitability, and white matter connectivity repair.^45,46,47,48,49^ This remodeling may modify pain perception, enhancing functional coordination; improving emotional regulation, daily activity, and pain coping; and thus promoting recovery. Previous research indicated inconsistent DMN changes in MWOA,^50,51,52^ possibly reflecting dysregulation in self-referential pain perception.^53^ Disrupted SC-pain region connectivity suggests impaired sensory integration or motor coordination,^54,55^ while motor network alterations may be related to dysregulated sensory-motor integration and pain processing.^12^ These dysfunctions collectively form migraine’s neural basis.

The study found that baseline DMN-SC hypoconnectivity and SC-motor hyperconnectivity predicted acupuncture efficacy. DMN-SC hypoconnectivity may indicate greater modulability of limbic-cerebellar pain appraisal,^14,56,57^ while SC-motor hyperconnectivity may reflect maladaptive cerebellar-motor compensation in migraine chronification.^58,59^ Acupuncture-associated normalization of these connectivities could reduce pain and improve motor function and disability, but causality remains unproven.

While CPM successfully predicted VAS and HIT-6 score changes, its predictive utility did not extend to other outcomes such as MMDs, MHDs, acute medication use days, and MSQ domains. This variation suggests the identified neuroimaging signatures are specifically sensitive to direct pain intensity and functional disability rather than to broader frequency or quality-of-life measures, which may involve more complex behavioral, environmental, or psychological factors that are not fully captured by baseline functional connectivity. It is possible that these more intricate outcomes require different neuroimaging modalities, advanced network analyses, or longitudinal data for accurate prediction.

CPM predicted which patients with MWOA benefit from acupuncture, identifying baseline connectivity signatures that refine therapeutic targets and support personalized treatment selection.^60,61^ The ability to predict treatment outcomes could transform patient care by improving treatment selection and identifying likely responders. Applying CPM to reduce the trial-and-error approach common in migraine management may promote more efficient use of health care resources and limit ineffective or unnecessary interventions.^62,63^

### Strengths and Limitations

This study has several strengths. First, we pioneered the application of an established whole-brain predictive model (CPM) to the field of migraine. This model can effectively capture complex brain connectivity patterns and reveal their associations with clinical outcomes. Second, we followed the TRIPOD+AI (Transparent Reporting of a Multivariable Prediction Model for Individual Prognosis or Diagnosis) and PROBAST (Prediction Model Risk of Bias Assessment Tool) guidelines strictly. By doing so, we ensured transparency of the predictive model and a systematic assessment of bias and thereby minimized reporting bias and enhanced the scientific rigor of the study.^64,65,66^

However, the study has several limitations to consider. First, to ensure sample homogeneity, we excluded patients with other types of migraine, which limited the sample size; therefore, caution is required when generalizing the findings to different clinical populations. Second, the therapeutic effects of acupuncture and the resulting changes in brain functional connectivity take time to emerge, and this study lacked posttreatment MRI data. Future studies should include such data to capture the temporal dynamics of whole-brain functional patterns.

## Conclusions

In this trial of acupuncture for MWOA that integrated CPM, we identified which patients were more likely to respond to treatment. Clinical results demonstrated acupuncture’s efficacy in pain relief and functional improvement. Neuroimaging analyses indicated that low DMN-SC connectivity was associated with pain relief and high SC-motor connectivity was associated with reduced disability. These findings uncovered baseline functional connectivity markers of better outcomes and offered objective neuroimaging biomarkers for personalized treatment.

## Notes

Supplement 1.
Statistical Analysis Plan

Supplement 2.
Study Protocol

Supplement 3. **eMethods 1.** Patient Recruitment and Study Sample Selection **eMethods 2.** Scales for Outcome Measures **eMethods 3.** Neuroimaging Data Acquisition **eMethods 4.** Image Preprocessing **eMethods 5.** Shen Atlas Registration **eMethods 6.** Network Anatomy **eAppendix.** Negative Results **eFigure 1.** The Real Acupoints Location **eFigure 2.** The Sham Acupoints Location **eFigure 3.** The Flowchart of Connectome-based Predictive Modeling **eTable 1.** The Real Acupoints Location **eTable 2.** The Manipulations of the Real Acupoints **eTable 3.** The Sham Acupoints Location **eTable 4.** Baseline Characteristics in the Intention-to-Treat Population **eTable 5.** Baseline Characteristics in the Per-Protocol Population **eTable 6.** Primary and Secondary Clinical Efficacy Outcomes in the Per-Protocol Population During the Treatment Period **eTable 7.** Participants’ Satisfaction at Week 4 **eTable 8.** Acupuncture Expectancy Scale Score at Baseline **eTable 9.** Compliance Data at Week 4 **eTable 10.** Participant-Blinding Assessment Results **eTable 11.** Adverse Events Related to Treatment
eReferences

Supplement 4.
Data Sharing Statement
