# Supplementary material for: Acupuncture for Migraine Without Aura and Connection-Based Efficacy Prediction: A Randomized Clinical Trial
Source: JAMA Netw Open. 2026 Jan 27;9(1):e2555454. doi: 10.1001/jamanetworkopen.2025.55454 (PMC12848631; doi:10.1001/jamanetworkopen.2025.55454)
Supplement: Supplement 1. — Statistical Analysis Plan [file jamanetwopen-e2555454-s001.pdf]

SUPPLEMENT 1: Statistical Analysis Plan

Acupuncture for Migraine Without Aura and Connection-Based Efficacy Prediction: A Randomized Clinical Trial

Contents

Statistical Analysis Plan.. 2

Acupuncture for Migraine Without Aura and Connection-Based Efficacy Prediction: A Randomized Clinical Trial

STATISTICAL ANALYSIS PLAN

Beijing Hospital of Traditional Chinese Medicine, Capital Medical University, Beijing, China

Principal Investigator

Lu Liu, MD

Version: 1.0

17 October 2024

TABLE OF CONTENTS

LIST OF ABBREVIATIONS ---------- 1
1.INTRODUCTION ---------- 2
2.STUDY OBJECTIVES AND OUTCOMES---------- 2
3.STUDY DESIGN ---------- 4
3.1 General Design ---------- 4
3.2 Sample Size and Power Considerations---------- 6
3.3 Randomization and Blinding---------- 6
4. DEFINITION---------- 6
5. ANALYSIS SETS ---------- 6
5.1 Intention-to-treat Analysis Set---------- 6
5.2 Per-protocol Analysis Set---------- 6
5.3 Safety Analysis Set ---------- 7
6. GENERAL ISSUES FOR DATA ANALYSIS ---------- 7
6.1 General---------- 7
6.2 Specification of Baseline Value ---------- 7
7. STUDY POPULATION SUMMARY ---------- 7
7.1 Participant Disposition ---------- 7
7.2 Withdrawals---------- 8
7.3 Demographics and Baseline Characteristics ---------- 8
7.4 Prior Medications or Therapy---------- 8
7.5 Diary Compliance---------- 8
8. PROTOCOL DEVIATIONS ---------- 8
9.EFFICACY ANALYSIS---------- 8
9.1 General---------- 8
9.2 Primary Efficacy Variable and Analysis---------- 9
9.2.1 Variable Definition---------- 9
9.2.2 Primary Efficacy Analysis ----------10
9.3 Secondary Efficacy Variables and Analysis ----------10
9.3.1 Variable Definition----------10
9.3.1.1. Headache Related Data During Treatment Period ----------11
9.3.1.1.1. Migraine Responder Rates----------11
9.3.1.1.2. Other Headache Related Data ----------11
9.3.1.2. Questionnaires During Treatment Period----------11
9.3.1.2.1. HIT-6 Headache Impact Test----------11
9.3.1.2.2 Migraine-Specific Quality of Life (MSQoL)----------12
9.3.1.2.3 Patient Global Impression of Change Scale (PGIC) ----------14
9.3.1.2.4 Acupuncture Expectancy Scale (AES) ----------14
9.3.1.2.5 Blinding assessment ----------14
9.3.2 Secondary Efficacy Analysis----------15
10. SAFETY ANALYSIS ----------15
10.1 General ----------15
10.2 Acupuncture ----------15
10.3 Adverse Events----------15

10.4 Allowed or Disallowed Concomitant Treatments --- 16
11. REFERENCES -- 18
APPENDIX--- 19
Appendix A. Headache Diary-- 19
Appendix B. Headache Impact Test-6 (HIT-6)--- 26
Appendix C.Migraine-Specific Quality Of Life Questionnaire (MSQ) (VERSION 2.1) 28
Appendix D. Patient Global Impression of Change Scale (PGIC) --------------------------- 33
Appendix E. Acupuncture Expectancy Scale (AES)-- 34
Appendix F. Blinding Questionnaire --- 35

LIST OF ABBREVIATIONS

Abbreviation	Definition
ACEI	Angiotensin-Converting Enzyme Inhibitor
AEs	Adverse events
AES	Acupuncture Expectancy Scale
ARB	Angiotensin Receptor Blocker
CGRP	Calcitonin gene related peptide
CIs	Confidence intervals
CPM	Connectome-based Predictive Modeling
CRF	Case Report Form
DMN	Default Mode Network
HIT-6	Headache Impact Test-6
ICHD-3	International Classification of Headache Disorders 3 version
ITT	Intention-to-Treat
LSM	Least-Squares Mean
MMDs	Monthly migraine days
MHDs	Monthly headache days
MRI	Magnetic Resonance Imaging
MSQoL	Migraine-Specific Quality of Life
MWoA	Migraine Without Aura
PGIC	Patient Global Impression of Change Scale
PP	Per-Protocol
RA	Real Acupuncture
RCTs	Randomized controlled trials
SA	Sham Acupuncture
SAE	Serious adverse event
SC	Subcortical-Cerebellum
SE	Standard error
SD	Standard deviation
SOP	Standard operating procedures
VAS	Visual Analog Scale
WHO	World Health Organization

1.INTRODUCTION

This Statistical Analysis Plan (SAP) describes the planned analysis and reporting based on the protocol for the clinical trial entitled: Acupuncture for Migraine Without Aura and Connection-Based Efficacy Prediction: A Randomized Clinical Trial. Study protocol version 1.0 was reviewed in preparation of this SAP.

The purpose of this SAP is to outline the planned analyses to be completed to support the completion of the trial reports. The planned analyses identified in this SAP will be included in future manuscripts.

2.STUDY OBJECTIVES AND OUTCOMES

Objectives	Outcomes
Primary Objectives<br>•To evaluate the efficacy of acupuncture in treating participants with MWoA	•Primary Outcomes<br>- Change from baseline in number of MMDs during weeks 1-4<br><br>•Secondary Outcomes<br>- ≥50% reduction in number of monthly migraine days during weeks 1-4<br>- Change from baseline in number of MHDs during weeks 1-4<br>- Change from baseline in VAS score at week 4<br>- Change from baseline in number of days with acute medication use during weeks 1-4
Secondary Objectives<br>•To evaluate the disability scores of participants<br><br>•To evaluate the quality of life of participants	•Secondary Outcomes<br>- Change from baseline in HIT-6 total score at week 4<br><br>- Change from baseline in MSQOL Role Function-Restrictive domain at week 4<br>- Change from baseline in MSQOL Role Function-Preventive domain at week 4<br>- Change from baseline in MSQOL Emotional Function domain at week 4
Safety Objectives<br>•To evaluate the safety of acupuncture	•Safety Outcomes<br>- Occurrence of adverse events throughout the

	study

3.STUDY DESIGN

3.1 General Design

This study is a single-blind randomized controlled trial in China to evaluate the efficacy of acupuncture for migraine and predict its efficacy from brain connectome data using machine learning.

This study will include female and male participants, aged 18 to 65 years, suffering from migraine for at least 1 year before screening. Participants meet migraine without aura (MWoA) diagnosis of the International Classification of Headache Disorders 3 version (ICHD-3) criteria[1]. The diagnosis will be prospectively confirmed via a review of headache diary recorded daily during a 4-week baseline period. The target population for this study is defined as patients with MWoA, as outlined in the International Headache Society ICHD-3 guidelines via prospectively collected information in the diary during the baseline period.

It is planned that 120 patients will be randomly allocated to one of two treatment groups: real acupuncture group (RA group) or sham acupuncture group (SA group), in a ratio of 1:1. The patient will receive treatment for 4 weeks. Patients assigned to RA group will receive acupuncture. Patients assigned to the SA group will receive sham acupuncture intervention. An overview of the study is presented in fig 1 and the scheduled study procedures and assessments are summarized in **table 1**.

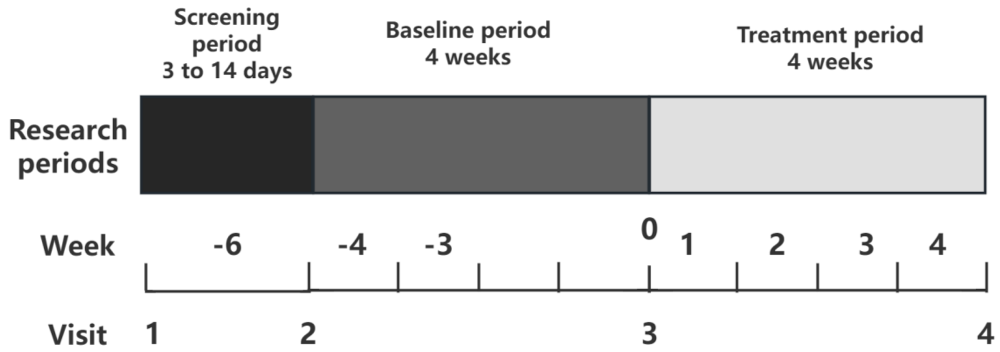

Fig 1. Study design. The study for each participant will be divided into 3 periods: a screening period (3 to 14 days), a 4-week baseline period, and a 4-week treatment period.

Table 1. The schedule of enrolment, interventions, and assessments

**STUDY PERIOD**							
	**Screening**	**Baseline**	**Allocation**	**Treatment**			
**TIMEPOINT(W, week)**	-6	-4	0	1	2	3	4
**Enrolment**							
Informed consent	×						
Eligibility criteria	×	×					
Demography Characteristics		×					
Disease history of migraine		×					
Randomization			×				
**Interventions**							
RA group				⟷			
SA group				⟷			
**Assessments**							
Headache diary		⟷					
HIT-6			×				×
MSQoL			×				×
Participants' satisfaction							×
AES			×				
Assessment of blinding				×			×
Participant's compliance				×	×	×	×
Adverse events				⟷			

HIT-6=Headache Impact Test; MSQoL=Migraine-Specific Quality of Life Questionnaire; AES=Acupuncture Expectancy Scale.

3.2 Sample Size and Power Considerations

According to previous studies[2], during the first to fourth cycles, the reduction of MMDs after treatment was 2.2±2.0 in the RA group and 1.6±3.0 in the SA group. To detect the difference between the two groups with a power of 80% at an alpha level of 0.05 and considering a 10% dropout rate, the sample size for randomization was determined to be 120 participants (60 in each treatment group).

3.3 Randomization and Blinding

After the baseline period, eligible participants will be randomly assigned (1:1) to either RA group or SA group using an interactive web-based response system (Beijing LNKMED Tech Co., Ltd). Randomization will be performed by a computer-generated random code with constant block size. The randomization sequence will be created by an independent biostatistician who did not participate in the study. The participants, outcome assessors, and the statistician will be blinded to treatment assignment.

4. DEFINITION

Definition of a MWoA day: A migraine day was defined as a calendar day with a headache lasting at least 4 consecutive hours, with features meeting ICHD-3 criteria (without aura) or probable migraine (subtype in which only one migraine criterion is absent), or a day in which acute migraine-specific medication (triptans or ergots derivatives) was used to treat a headache of any duration.

Definition of a headache day: A headache day is defined as any calendar day on which a qualified migraine headache or a non-migraine headache (onset, continuation, or recurrence of the headache) attacked lasting at least 4 consecutive hours, or a headache attack of any duration with acute headache medication (non-steroidal anti-inflammatory drugs; triptans; ergotamine and derivatives; acetaminophen; or combinations thereof) given to treat headache pain.

Definition of response rates: Proportion of patients with ≥50% reduction from baseline in monthly migraine days in each period.

Definition of acute headache medication day: Any calendar day during which the subject took an acute headache medication.

5. ANALYSIS SETS

5.1 Intention-to-treat Analysis Set

The Intention-to-Treat (ITT) analysis set will include all randomized participants. In this population, treatment will be assigned based on the treatment to which participants are randomized, regardless of which treatment they actually received.

5.2 Per-protocol Analysis Set

The per-protocol (PP) analysis set consisted of all participants who met all eligibility criteria and were randomized, complete the treatment and follow-up plan, and were without any major protocol deviations.

5.3 Safety Analysis Set

The safety analysis set will include all participants who receive at least one treatment. In this population, treatment will be assigned based upon the treatment participants actually received, regardless of the treatment to which they are randomized.

6. GENERAL ISSUES FOR DATA ANALYSIS

6.1 General

All summary statistics will be computed and displayed by treatment group. In general, summary statistics (count [n], mean, least-squares mean [LSM], median [interquartile range], standard deviation [SD], standard error [SE]) will be presented for continuous variables and counts and, if relevant, percentages will be presented for categorical variables. Graphical data displays may also be used as appropriate.

6.2 Specification of Baseline Value

The baseline efficacy variables will be summarized into diary-reported baseline migraine characteristics and other efficacy variables. Participants with ≥14 days of non-missing diary data (≥50% of 28 days) will have counts prorated to 28 days. Those with <14 days of non-missing diary data will be treated as having missing baseline values.

The efficacy baseline values during the 28-day baseline period derived from the headache diary include:

1. disease duration, years
2. number of monthly migraine days (MMDs) at baseline
3. number of monthly headache days (MHDs) at baseline
4. number of monthly days with acute headache medication use at baseline
5. acute headache medication overuse at baseline

The other baseline efficacy variables that will be summarized include：

1. disability-related

- Headache Impact Test (HIT-6) total score at baseline

2. quality of life-related

- Migraine-Specific Quality of Life (MSQoL) Role Function-Restrictive domain at baseline
- MSQoL Role Function-Preventive domain at baseline
- MSQoL Emotional Function domain at baseline

7. STUDY POPULATION SUMMARY

7.1 Participant Disposition

Data from participants screened, participants screened but not randomized and reason not randomized, participants who are randomized (i.e., in the ITT set), participants randomized but not treated, participants in the safety and other analysis sets, participants who complete the study, and participants

who withdraw from the study will be summarized using descriptive statistics. Data from participants who withdraw from the study will also be summarized by reason for withdrawal using descriptive statistics.

7.2 Withdrawals

For each period, the number of participants who withdraw from treatment, which also implies that they withdraw from the study, will be summarized by group, and primary reason for withdrawal, and by treatment group and all reasons for withdrawal.

Participants who withdraw will be listed for each period. The listings will include the number of days in the study until withdrawal, the date of first treatment, the date of the latest treatment prior to withdrawal, the number of days since latest treatment, the primary reason for withdrawal, all reasons for withdrawal.

7.3 Demographics and Baseline Characteristics

Participant's demographics and baseline characteristics, including age, sex, time since initial migraine diagnosis, and any acute headache medication use, will be summarized for the ITT population. The baseline diary efficacy variables listed in section 6.2, HIT-6 total score, MSQoL scores, will be summarized by treatment group for the ITT set.

7.4 Prior Medications or Therapy

The incidence of prior medications or therapy will be summarized using descriptive statistics by therapeutic class. Participants are counted only once in each therapeutic class category. Prior medications will be summarized for the categories as preventive migraine medication and acute headache medication.

7.5 Diary Compliance

For each period, the rate of days where the diary has been missed within each 4-week interval will be summarized and presented by 4-week interval and treatment group. Furthermore, the number of participants missing 14 days or more in a 28-day period will be presented by 4-week interval, separately for each period. A day where the diary has been missed is defined as a day where the subject did not fill out anything in the headache diary.

8. PROTOCOL DEVIATIONS

Protocol violations include enrolling participants in violation of key eligibility criteria designed to ensure a specific subject population, failing to collect data necessary to interpret outcomes, or any other deviations that may have an impact on the processes put in place for the care and safety of the participants or compromise the scientific value of the trial.

9.EFFICACY ANALYSIS

9.1 General

The efficacy data for this study consist of headache related questions responses (e.g., occurrence of headache, duration of headache in each day, maximum severity of headache, and acute headache medication use) collected daily by headache diary (**Appendix A**).

In addition, the following questionnaires will be used for the assessments of migraine disability, quality of life and psychological profile during the study (see table 1 for the schedule of the assessments).
- Disability using the HIT-6 (**Appendix B**)
- Quality of life measured by the MSQoL questionnaire (**Appendix C**)

The primary analysis will be performed on the ITT set.

9.2 Primary Efficacy Variable and Analysis

For the purpose of this study, a migraine day will be defined as a calendar day with a headache lasting at least 4 consecutive hours, with features meeting ICHD-3 criteria (without aura) or probable migraine (subtype in which only one migraine criterion is absent), or a day in which acute migraine-specific medication (triptans or ergots derivatives) was used to treat a headache of any duration.

9.2.1 Variable Definition

The primary efficacy variable is change from baseline in number of monthly migraine days (MMDs) during weeks 1-4. The following describes the derivation of MMDs. The derivation of monthly headache days follows the same principles.

4-Week Intervals

For Baseline and Treatment in the study, the number of MMDs will be derived as the number of migraine days within each 28-day interval using the imputation rules described below. The 4-week periods post-baseline that are considered are the following: Weeks 1-4.

In general, for 4-week periods where the diary is completed on at least 14 days out of the 28 days, prorating will be used to calculate the MMDs, and for 4-week periods where the diary is completed on less than 14 days out of the 28 days, the MMDs will be set to missing.

Prorating

Intended to be used for 4-week periods where the diary is completed on at least 14 out of the 28 days, the prorating procedure consists of imputing days with missing information with the observed mean number of migraine days in the period as follows:

$$28 \times \left(\frac{Reported\ Migraine\ Days}{Reported\ diary\ Days}\right)$$

This imputation rule will also be used for the baseline value regardless of the number of reported diary days in the baseline period. Participants are, however, required to demonstrate compliance with the diary and fill it out for at least 24 out of the 28 days in the baseline period in order to be eligible for

randomization.

In case of a reporting day where a participant answers in the diary that they experienced a headache on that day but then never fills out an actual headache in the headache diary, the day will be assigned as a no headache and no migraine day.

When the number of MMDs have been calculated for a given 4-week period, the result will be rounded to two decimals.

Multiple imputation

For participants with missing days and fewer than 14 days of headache diary data for 28 days, the MMDs were considered missing before the multiple imputation procedure. The statistics were based on 10 sets of imputed data, where the mean is the average of the means from the 10 data sets and the standard error (SE) of the mean is adjusted based on the imputation variance estimates.

9.2.2 Primary Efficacy Analysis

The hypothesis testing for the primary analysis is:

$$H_0: \quad \delta_1 = \delta_2$$

$$H_1: \quad \delta_1 \neq \delta_2$$

where δ1 and δ2 are the estimates of change from baseline in number of MMDs for the treatment group and the control group respectively.

Continuous variables were first assessed for distributional characteristics. For outcomes that departed from normality, comparisons between the RA and SA groups were performed using the Wilcoxon rank-sum test (Mann-Whitney U), and the Hodges-Lehmann estimate was used as the point estimate of the median difference; the Hodges-Lehmann estimate and its 95% confidence interval were derived using the Wilcoxon test's normal-approximation method. All tests were two-sided with $\alpha = 0.05$. Results are presented as median and interquartile range (IQR; 25th-75th percentiles) for each group, with between-group differences given as the Hodges-Lehmann estimate and its 95% confidence interval (CI) together with the two-sided P-value. For outcomes that were approximately normally distributed, comparisons were made with independent-samples t-tests; results are reported as mean change with standard error (SE) and 95% CI, and between-group differences as mean difference with 95% CI and two-sided P-value. The proportion of responders with $\geqslant$ 50% reduction in MMDs was assessed using a logistic regression model. For this outcome at week 12, percentages with SE and odds ratios (OR) with 95% CI were presented.

9.3 Secondary Efficacy Variables and Analysis

9.3.1 Variable Definition

9.3.1.1. Headache Related Data During Treatment Period

9.3.1.1.1. Migraine Responder Rates

The following describes the derivation for migraine responder rates.

The following responder rates will be derived: 50%. A responder is a participant, who achieves a ≥50% reduction in MMDs, compared to the baseline number of MMDs. The population-level summary is the odds ratio of a successful response between two groups. The derivation of these responder outcomes will be based on the number of MMDs resulting from the imputations described in section 9.2.1.

For the 4-week period post-baseline in the study, the responder status of a participant will be derived based on the percentage change from baseline in MMDs. If the MMDs value is missing for the month in question, the response status will also be missing.

For the 4-week interval, the 50% responder status will be derived as follows:

$$50\%\ Responder\ Status(Weeks1-4)=\begin{cases}1, & if\dfrac{ave(\Delta_M)}{Baseline}\le -0.5\\ 0, & if\dfrac{ave(\Delta_M)}{Baseline}> -0.5\end{cases}$$

where ΔM is the change from baseline value for week 4.

For the secondary outcome of ≥50% reduction in the number of monthly migraine days with any patient that does not have a value of monthly migraine days will be imputed with a non-response.

9.3.1.1.2. Other Headache Related Data

The change from baseline in number of monthly days/hours of secondary efficacy variables (headache days, days with acute headache medication use) during the treatment period will be derived similar to the primary variable using the diary data collected through the corresponding headache diary questions.

9.3.1.2. Questionnaires During Treatment Period

9.3.1.2.1. HIT-6 Headache Impact Test

Migraine related disability will be assessed using the HIT-6 completed before the first treatment, the end of treatment, and the end of follow-up period[3]. Each participant will answer the HIT-6 questionnaire questions to measure the impact headaches that have on his/her ability to function on the job, at school, at home and in social situations. Each question is answered on the scale ranging with the following response options: 6 points (never), 8 points (rarely), 10 points (sometimes), 11 points (very often), and 13 points (always). The total score is obtained from summation of the 6 question points. The HIT-6 total score ranges between 36 and 78, with larger scores reflecting greater impact. If 1 or more items are

missing, then the total score is missing.

9.3.1.2.2 Migraine-Specific Quality of Life (MSQoL)

The self-administered, migraine-specific, 14-item MSQoL questionnaire is designed to measure how migraines affect and/or limit daily functioning across 3 domains: Role Function-Restrictive domain comprising 7 items assessing how migraines limit one's daily social and work-related activities; Role Function-Preventive domain comprising 4 items assessing how migraines prevent these activities, and Emotional Function domain comprising 3 items assessing the emotions associated with migraines[4]. Items are rated on a six-point scale (none of the time, a little bit of the time, some of the time, a good bit of the time, most of the time, and all of the time). The raw dimension scores are computed as a sum of item responses and rescaled to a 0 to 100 scale such that higher scores indicate better quality of life and improvement.

The items going into each domain are specified in **table 2**. Each item score is mapped from the recorded value of the item as shown in **table 3**. For each domain, the score is derived from the final item values by summing the scores from items within each domain and transforming the summed scores as shown in **table 4**.

Table 2. Items in Each Domain of MSQOL

Domain	Item number	Abbreviated content
Role function restrictive	1	. . . . . interfered with how well you dealt with family, friends, and others
	2	. . . . . interfered with your leisure time activities such as reading or exercising
	3	. . . . . had difficulty in performing work or daily activities
	4	. . . . . kept you from getting as much done at work or at home
	5	. . . . . limited your ability to concentrate on work or daily activities
	6	. . . . . left you too tired to do work or daily activities
	7	. . . . . limited the number of days you felt energetic
Role function preventive	8	. . . . . canceled work or daily activities . . .
	9	. . . . . needed help in handling routine tasks
	10	. . . . . stopped work or daily activities . . .
	11	. . . . . not able to go to social activities . . .
Emotional function	12	. . . . . felt fed up or frustrated
	13	. . . . . felt like a burden on others
	14	. . . . . afraid of letting others down

Table 3. MSQOL Item Values

Response	Categories Precoded item value	Final item value
None of the time	1	6
A little bit of the time	2	5
Some of the time	3	4
A good bit of the time	4	3
Most of the time	5	2
All of the time	6	1

Table 4. Derivation of MSQOL Domain Scores

Domain	Sum of item scores range	Derivation
Role function restrictive	7 to 42	(Summed score – 7) ×100/35
Role function preventive	4 to 24	(Summed score – 4) ×100/20
Emotional function	3 to 18	(Summed score – 3) ×100/15

9.3.1.2.3 Patient Global Impression of Change Scale (PGIC)

The PGIC scale is a validated generic tool for assessment of patient satisfaction. Patients will rate how they describe the change (if any) that their migraine/headaches have had in their general quality of life and health status since beginning the treatment in this study on a 7-point scale where 1=no change (or condition got worse); 2=almost the same, hardly any change at all; 3=a little better, but no noticeable change; 4=somewhat better, but the change has not made any real difference; 5=moderately better, and a slight but noticeable change; 6=better, and a definite improvement that has made a real and worthwhile difference; and 7=a great deal better, and a considerable improvement that has made all the difference (**Appendix D**).

9.3.1.2.4 Acupuncture Expectancy Scale (AES)

The AES consists of four items measuring the expectation of improvement of illness (symptom), enhanced coping, increased vitality, and symptom alleviation due to acupuncture therapy. Patients will be asked to rate from 1 to 5 on a five-point Likert scale, with 1 indicating "Not at all agree" and 5 indicating "Completely agree" with the expected improvement as result of acupuncture. The score of instrument range from 4 to 20 out of a possible 4 to 20, with higher scores indicating greater expectancy (**Appendix E**).

9.3.1.2.5 Blinding assessment

To test the success of blinding, within 5 minutes after treatment at week 1 and at week 4, participants will be told that there are two kinds of treatment groups: "RA group" and "SA group", and they will be randomly assigned to either group at 50% chance respectively. Participants will then be asked to answer the question "Do you think which kind of treatment group you have participated in during the past weeks?" The participants will be able to choose one of the following options as the answer: "Think in RA group", "Think in SA group" or "Did not know". (**Appendix F**).

The Bang blinding indices will be used to assess the success of blinding. The Bang blinding index for each group represents the proportion of participants making a correct treatment guess beyond chance; 0 represents perfect blinding, a positive index indicates a correct guess, and a negative index indicates a guess in the opposite direction.

9.3.2 Secondary Efficacy Analysis

The proportion of participants with 50% or greater reduction in MMDs was assessed using a logistic regression model. The analysis of continuous secondary outcomes were performed similarly to the primary efficacy outcome.

10. SAFETY ANALYSIS

10.1 General

The safety population will be used for all safety analyses. Summaries will be presented by treatment group unless specified otherwise.

10.2 Administration of Acupuncture

Following the baseline assessments, eligible participants will be randomly assigned (1:1) with RA group or SA group.

Acupuncture treatment will be performed by two licensed acupuncturists who had at least 20 years of acupuncture experience for twelve 30-minute sessions (3 sessions per week, ideally every other weekday) during 4 weeks. Acupoints is as follows:

In the RA group, Baihui (GV20), Fengfu (GV16), bilateral Fengchi (GB20), bilateral Taiyang (EX-HN5), bilateral Hegu (LI4) are selected as acupoints. Sterilized, single-use needles (Hwato Needles, made in Suzhou, China) will be used for acupuncture in this trial. The number of needles will be 8 in each session for both groups. Needles of 0.30mm in diameter and 40mm in length will be used for limb acupoints, and needles of 0.25/0.30mm in diameter and 25mm in length will be used for head acupoints. All needles will be inserted 10-15mm in depth and twirling lifting, and thrusting (needle manipulation) will be performed for at least 10 seconds and repeated a total of 4 times with an interval of 10 minutes to produce a characteristic sensation known as Deqi (a sensation of soreness, numbness, distention, or heaviness that indicates effective needling). While the acupuncture in the SA group is performed using the same methods as in the RA group, non-effective acupoints are selected, and the Deqi manipulation is not used.

10.3 Adverse Events

For AE recording, the study period is defined for each participant as the time period from signature of the informed consent form through completion of week 4 or the early withdrawal visit for participants who withdraw from the study for any reason.
AEs must be recorded on an Adverse Event Form through the entire study. AEs will be collected at each contact with the participant via AE inquiry.

The following are considered protocol-defined AEs:

• All AEs associated with acupuncture include broken needle, needle phobia, needling pain after treatment, numbness, intense pricking, pricking lasting more than half an hour (no matter how intense it is) after acupuncture, subcutaneous hematoma, bleeding, infection, abscess formation at the needling site, other discomfort induced by acupuncture (such as fatigue, drowsiness, nausea, vomiting, palpitation, dizziness, headache, loss of appetite, insomnia, etc.), and aggravation of existing symptoms, etc.
• intercurrent illnesses
• physical injuries
• events possibly related to concomitant medication
• significant worsening (change in nature, severity, or frequency) of the disease under study or other pre-existing conditions (Note: A condition recorded as pre-existing that is intermittently symptomatic [e.g., headache] and that occurs during this study should be recorded as an AE.)
• drug interactions
• events occurring during diagnostic procedures of this study
•migraine exacerbations, including acute headaches, requiring headache medications will be collected as part of the efficacy assessment in this study. Migraine exacerbations (including acute headaches) should be recorded as an AE only if the presentation and/or outcome is more severe than would typically be expected from the normal course of the disease in a particular patient or if they are severe enough to require hospitalization of the patient, in which case they are recorded as SAEs.

Summaries by treatment group will be presented for AEs (overall and by severity), AEs determined by the investigator to be treatment-related AEs (overall and by severity), SAEs.

The incidence of AE and severity of the AE will be summarized using descriptive statistics. Each patient will be counted only once by using the AEs with the highest severity within each category. Treatment-related AE summaries will include AEs related to medication and acupuncture.

Listings for SAEs, AEs will be presented. All information pertaining to AEs noted during the study will be listed by subject, detailing verbatim given by the investigator, date of onset, date of resolution, severity, and relationship to treatment. The onset of AEs will also be shown relative (in number of days) to the first day of treatment. In addition, AE descriptions, and AE by patient number and treatment group will be presented.

10.4 Allowed or Disallowed Concomitant Treatments

Throughout the whole trial, participants will be disallowed from any preventive treatments of migraine, such as beta blockers, anticonvulsants, tricyclic antidepressants, calcium channel blockers, angiotensin-converting enzyme inhibitor / angiotensin receptor blocker, onabotulinumtoxin A, valproate, acupuncture, occipital stimulator, nerve blocks and transcranial magnetic stimulation. Any other treatment, not affect migraine but has been used in the period of study, related information

should be recorded in CRF.

11. REFERENCES

1. Headache Classification Committee of the International Headache Society (IHS) The International Classification of Headache Disorders, 3rd edition. *Cephalalgia*. 2018;38(1)doi:10.1177/0333102417738202

2. Xu S, Yu L, Luo X, et al. Manual acupuncture versus sham acupuncture and usual care for prophylaxis of episodic migraine without aura: multicentre, randomised clinical trial. *BMJ*. 2020;368:m697. doi:10.1136/bmj.m697

3. Kosinski M, Bayliss MS, Bjorner JB, et al. A six-item short-form survey for measuring headache impact: the HIT-6. *Qual Life Res*. 2003;12(8):963-974.

4. Speck RM, Yu R, Ford JH, Ayer DW, Bhandari R, Wyrwich KW. Psychometric validation and meaningful within-patient change of the Migraine-Specific Quality of Life questionnaire version 2.1 electronic patient-reported outcome in patients with episodic and chronic migraine. *Headache*. 2021;61(3):511-526. doi:10.1111/head.14031

APPENDIX

Appendix A. Headache Diary

**Study Period**<br>（Please draw √ inside the present period brackets）	**Baseline period**<br>(-4-0 Week)<br>( )	**Treatment period**<br>(1-4 Week)<br>( )

Subject Diary Card - Headache diary

Random Number：____________________________

Time of This Visit：____________________(yy/mm/dd)

[Filling Explanation]

Your headache diary is used to record all the symptoms associated with your headache. Please try your best to answer these questions on the day of headache or the day after headache. You need to record all symptoms you observe for 4 weeks. Please try your best to ensure accuracy and completeness. Your headache diary can help your doctor develop the right treatment strategy. Your symptoms may not be obvious during this time, but once you document each of your symptoms, your pattern of symptoms and the progression of your disease over time will give a strong indication of your condition.

Your charge doctor:________________________________

Tel of your charge doctor： ___________________________

Explanation 1: Please record a headache diary **at 19:00** every day and recall your headache from 19:00 yesterday to now **without blank**. If you have a headache, please truthfully record it in the diary, and be sure to record all the details. And remember to **bring back** this headache diary on each subsequent visit. The following are the problems you may encounter when filling. If you have any questions during the filling process, please contact your doctor in charge.

• If your headache has not ended at the time of filling, please fill in "**Still continuing**" in the “End time” column

• If the headache doesn’t happen, please fill in with “**No Headache**” in the “Start time” and “End time” column.

• If you do not have headache but **have aura**, please truthfully record the aura and the medication intake.

• If your headache **recurred or disappeared within 1 day**, please fill in the start time of the earliest headache in the “Start time” column and the end time of the headache closest to the time you record today in the “End time” column.

• If the duration of a headache is **longer than 24:00 of the previous day**, when recording the headache diary on the second day, please fill in the start time of the earliest headache of the previous day in the “Start time” column and the end time of the headache closest to the time you record today in the “End time” column.

• If you **did not fill in the diary for some reason in the previous day**, please fill in the headache condition of the previous day truthfully on the second day. If you have missed records from the previous 2 or more days, you can only fill in the latest 1 day headache diary.

Explanation 2: Aura:

• **Optic aura**: visual disturbance as spots, stars, flashes, zigzag lines, heat waves or complete or partial loss of vision around the time of your headache.

• **Sensory aura:** feeling disturbance as numbness or tingling in any part of your body or face around the time of your headache.

• The discomfort in the head and neck is excluded from aura for the reason that it indicates the beginning of headache.

Explanation 3： Intensity scale:

• 0 = No pain • 1(Mild) = Not affect daily activities • 2 (moderate) = Affect daily activities

• 3 (Severe) = Unable to perform daily activities.

Explanation 4：VAS score:

Suppose 0 is no pain and 10 is the worst pain you can imagine in the world.

VAS score: please read the severity of your headache in the position:

No pain └──┴──┴──┴──┴──┴──┴──┴──┴──┴──┘ the worst pain

0 1 2 3 4 5 6 7 8 9 10

Explanation 5: If you still feel headache when you go to sleep the day before, and the headache disappears when you wake up the next morning, the end time of headache is counted as the time when you wake up the next day.

Explanation 6: Please fill in the time in **24 hours format** : year, month, day, hour.

Explanation 7： The name of the drug should be filled in the record of medication intake,? tablet/time, ? time/day, a total of ? day.

Explanation 8: Triggers of Headache (Headache related climate change, environment change, physical discomfort, stress, tiredness, special diet, menstruation, etc.)

Explanation 9：Female subjects please fill in your menstrual start and end time:_____to

**Headache diary table**									
**Symptom** / **Date**									
**Headache duration**	**Start time**								
	**End time**								
**Intensity scale（0-3）**	**0-3***<br>**(Explanation 2)**								
**VAS**	**0-10***<br>**(Explanation 3)**								
**Was it worse on one side of the head than on the other, and/or limited to one side of the head?**		☐Yes<br>--☐Left<br>--☐Right<br>☐No	☐Yes<br>--☐Left<br>--☐Right<br>☐No	☐Yes<br>--☐Left<br>--☐Right<br>☐No	☐Yes<br>--☐Left<br>--☐Right<br>☐No	☐Yes<br>--☐Left<br>--☐Right<br>☐No	☐Yes<br>--☐Left<br>--☐Right<br>☐No	☐Yes<br>--☐Left<br>--☐Right<br>☐No
**Headache site**	**(Left, right, temporal, parietal, occipitalia, forehead)**	☐Left<br>☐Right<br>☐Temporal<br>☐Parietal<br>☐Occipitalia	☐Left<br>☐Right<br>☐Temporal<br>☐Parietal<br>☐Occipitalia	☐Left<br>☐Right<br>☐Temporal<br>☐Parietal<br>☐Occipitalia	☐Left<br>☐Right<br>☐Temporal<br>☐Parietal<br>☐Occipitalia	☐Left<br>☐Right<br>☐Temporal<br>☐Parietal<br>☐Occipitalia	☐Left<br>☐Right<br>☐Temporal<br>☐Parietal<br>☐Occipitalia	☐Left<br>☐Right<br>☐Temporal<br>☐Parietal<br>☐Occipitalia

		☐Forehead	☐Forehead	☐Forehead	☐Forehead	☐Forehead	☐Forehead	☐Forehead
**Was it pulsating?**		☐Yes<br>☐No	☐Yes<br>☐No	☐Yes<br>☐No	☐Yes<br>☐No	☐Yes<br>☐No	☐Yes<br>☐No	☐Yes<br>☐No
**Aggravation after daily activities(Walk or climb stairs, etc.)**		☐Yes<br>☐No	☐Yes<br>☐No	☐Yes<br>☐No	☐Yes<br>☐No	☐Yes<br>☐No	☐Yes<br>☐No	☐Yes<br>☐No
**Accompanying symptom**	**Nausea**	☐Yes<br>☐No	☐Yes<br>☐No	☐Yes<br>☐No	☐Yes<br>☐No	☐Yes<br>☐No	☐Yes<br>☐No	☐Yes<br>☐No
	**Vomit**	☐Yes<br>☐No	☐Yes<br>☐No	☐Yes<br>☐No	☐Yes<br>☐No	☐Yes<br>☐No	☐Yes<br>☐No	☐Yes<br>☐No
	**Light intolerance**	☐Yes<br>☐No	☐Yes<br>☐No	☐Yes<br>☐No	☐Yes<br>☐No	☐Yes<br>☐No	☐Yes<br>☐No	☐Yes<br>☐No
	**Noise intolerance**	☐Yes<br>☐No	☐Yes<br>☐No	☐Yes<br>☐No	☐Yes<br>☐No	☐Yes<br>☐No	☐Yes<br>☐No	☐Yes<br>☐No
	**Other symptoms**	(_______)	(_______)	(_______)	(_______)	(_______)	(_______)	(_______)
**Aura* (Explanation 2)**<br>(Please write the specific aura in the parentheses)		☐Without<br>☐Optic<br>(_______)<br>☐Sensory<br>(_______)	☐Without<br>☐Optic<br>(_______)<br>☐Sensory<br>(_______)	☐Without<br>☐Optic<br>(_______)<br>☐Sensory<br>(_______)	☐Without<br>☐Optic<br>(_______)<br>☐Sensory<br>(_______)	☐Without<br>☐Optic<br>(_______)<br>☐Sensory<br>(_______)	☐Without<br>☐Optic<br>(_______)<br>☐Sensory<br>(_______)	☐Without<br>☐Optic<br>(_______)<br>☐Sensory<br>(_______)

		☐Other (_______)	☐Other (_______)	☐Other (_______)	☐Other (_______)	☐Other (_______)	☐Other (_______)	☐Other (_______)
**Whether you want to lie down during headache? If yes, please fill in the affected time (hours).**		☐Yes ( )h ☐No	☐Yes ( )h ☐No	☐Yes ( )h ☐No	☐Yes ( )h ☐No	☐Yes ( )h ☐No	☐Yes ( )h ☐No	☐Yes ( )h ☐No
**Whether your daily study or work are impaired during headache? If yes, please fill in the affected time (hours) and the percent impaired during headache.**		☐Yes ( )h ( )% ☐No	☐Yes ( )h ( )% ☐No	☐Yes ( )h ( )% ☐No	☐Yes ( )h ( )% ☐No	☐Yes ( )h ( )% ☐No	☐Yes ( )h ( )% ☐No	☐Yes ( )h ( )% ☐No
**Whether your daily activities are impaired during headache? If yes, please fill in the affected time (hours) and the percent impaired during headache.**		☐Yes ( )h ( )% ☐No	☐Yes ( )h ( )% ☐No	☐Yes ( )h ( )% ☐No	☐Yes ( )h ( )% ☐No	☐Yes ( )h ( )% ☐No	☐Yes ( )h ( )% ☐No	☐Yes ( )h ( )% ☐No
**Triggers of headache* (Explanation 8)**								
**Analgesic drugs intake**	**Name**	☐Triptan (_______) ☐Ergot (_______)	☐Triptan (_______) ☐Ergot (_______)	☐Triptan (_______) ☐Ergot (_______)	☐Triptan (_______) ☐Ergot (_______)	☐Triptan (_______) ☐Ergot (_______)	☐Triptan (_______) ☐Ergot (_______)	☐Triptan (_______) ☐Ergot (_______)

		☐Other (________)	☐Other (________)	☐Other (________)	☐Other (________)	☐Other (________)	☐Other (________)	☐Other (________)
	**Dose and frequency**							
	**Headache 0.5 hour after taking analgesic**	☐disappear ☐remission ☐no change	☐disappear ☐remission ☐no change	☐disappear ☐remission ☐no change	☐disappear ☐remission ☐no change	☐disappear ☐remission ☐no change	☐disappear ☐remission ☐no change	☐disappear ☐remission ☐no change
	**Headache 2 hours after taking analgesic**	☐disappear ☐remission ☐no change	☐disappear ☐remission ☐no change	☐disappear ☐remission ☐no change	☐disappear ☐remission ☐no change	☐disappear ☐remission ☐no change	☐disappear ☐remission ☐no change	☐disappear ☐remission ☐no change
	**Recovery of daily activity ability 2 hours after taking analgesic**	☐Unrecovered ☐Basically recovered	☐Unrecovered ☐Basically recovered	☐Unrecovered ☐Basically recovered	☐Unrecovered ☐Basically recovered	☐Unrecovered ☐Basically recovered	☐Unrecovered ☐Basically recovered	☐Unrecovered ☐Basically recovered
**Other drug, treatment or methods were used**								
**Adverse event**								

Appendix B. Headache Impact Test-6 (HIT-6)

HIT is a tool used to measure the impact headaches have on your ability to function on the job, at school, at home and in social situations. Your score shows you the effect that headaches have on normal daily life and your ability to function. HIT was developed by an international team of headache experts from neurology and primary care medicine in collaboration with the psychometricians who developed the SF-36 health assessment tool. This questionnaire was designed to help you describe and communicate the way you feel and what you cannot do because of headaches.

To complete, please circle one answer for each question.

When you have headaches, how often is the pain severe?

never rarely sometimes very often always

How often do headaches limit your ability to do usual daily activities including household work, work, school, or social activities?

never rarely sometimes very often always

When you have a headache, how often do you wish you could lie down?

never rarely sometimes very often always

In the past 4 weeks, how often have you felt too tired to do work or daily activities because of your headaches?

never rarely sometimes very often always

In the past 4 weeks, how often have you felt fed up or irritated because of your headaches?

never rarely sometimes very often always

In the past 4 weeks, how often did headaches limit your ability to concentrate on work or daily activities?

never rarely sometimes very often always

☐ +	☐ +	☐ +	☐ +	☐
COLUMN 1	COLUMN 2	COLUMN 3	COLUMN 4	COLUMN 5
6 points each	8 points each	10 points each	11 points each	13 points each

To score, add points for answers in each column.

If your HIT-6 is 50 or higher:

You should share your results with your doctor. Headaches that stop you from enjoying the important things in life, like family, work, school or social activities could be migraine.

TOTAL

SCORE

Appendix C. Migraine-Specific Quality Of Life Questionnaire (MSQ) (VERSION 2.1)

While answering the following questions, please think about all migraine attacks you may have had in the past 4 weeks.

1. In the past 4 weeks, how often have migraines **interfered** with how well you dealt with family, friends and others who are close to you? (Select only **one** response.)

1☐ None of the time

2☐ A little bit of the time

3☐ Some of the time

4☐ A good bit of the time

5☐ Most of the time

6☐ All of the time

2. In the past 4 weeks, how often have migraines **interfered** with your leisure time activities, such as reading or exercising? (Select only **one** response.)

1☐ None of the time

2☐ A little bit of the time

3☐ Some of the time

4☐ A good bit of the time

5☐ Most of the time

6☐ All of the time

3. In the past 4 weeks, how often have you had **difficulty** in performing work or daily activities because of migraine symptoms? (Select only **one** response.)

1☐ None of the time

2☐ A little bit of the time

3☐ Some of the time

4☐ A good bit of the time

5☐ Most of the time

6☐ All of the time

4. In the past 4 weeks, how often did migraines **keep you** from getting as much done at work or at home? (Select only **one** response.)

1☐ None of the time

2☐ A little bit of the time

3☐ Some of the time

4☐ A good bit of the time

5☐ Most of the time

6☐ All of the time

5. In the past 4 weeks, how often did migraines **limit** your ability to concentrate on work or daily activities? (Select only **one** response.)

1☐ None of the time

2☐ A little bit of the time

3☐ Some of the time

4☐ A good bit of the time

5☐ Most of the time

6☐ All of the time

6. In the past 4 weeks, how often have migraines **left you too tired** to do work or daily activities? (Select only **one** response.)

1☐ None of the time

2☐ A little bit of the time

3☐ Some of the time

4☐ A good bit of the time

5☐ Most of the time

6☐ All of the time

7. In the past 4 weeks, how often have migraines **limited** the number of days you have felt energetic?

(Select only **one** response.)

1☐ None of the time

2☐ A little bit of the time

3☐ Some of the time

4☐ A good bit of the time

5☐ Most of the time

6☐ All of the time

8. In the past 4 weeks, how often have you had to **cancel** work or daily activities because you had a migraine? (Select only **one** response.)

1☐ None of the time

2☐ A little bit of the time

3☐ Some of the time

4☐ A good bit of the time

5☐ Most of the time

6☐ All of the time

9. In the past 4 weeks, how often did you **need help** in handling routine tasks such as every day household chores, doing necessary business, shopping, or caring for others, when you had a migraine? (Select only **one** response.)

1☐ None of the time

2☐ A little bit of the time

3☐ Some of the time

4☐ A good bit of the time

5☐ Most of the time

6☐ All of the time

10. In the past 4 weeks, how often did you have to **stop** work or daily activities to deal with migraine

symptoms? (Select only **one** response.)

1☐ None of the time

2☐ A little bit of the time

3☐ Some of the time

4☐ A good bit of the time

5☐ Most of the time

6☐ All of the time

11. In the past 4 weeks, how often were you **not able to go** to social activities such as parties, dinner with friends, because you had a migraine? (Select only **one** response.)

1☐ None of the time

2☐ A little bit of the time

3☐ Some of the time

4☐ A good bit of the time

5☐ Most of the time

6☐ All of the time

12. In the past 4 weeks, how often have you **felt** fed up or frustrated because of your migraines? (Select only **one** response.)

1☐ None of the time

2☐ A little bit of the time

3☐ Some of the time

4☐ A good bit of the time

5☐ Most of the time

6☐ All of the time

13. In the past 4 weeks, how often have you **felt** like you were a burden on others because of your

migraines? (Select only **one** response.)

1☐ None of the time

2☐ A little bit of the time

3☐ Some of the time

4☐ A good bit of the time

5☐ Most of the time

6☐ All of the time

14. In the past 4 weeks, how often have you been **afraid** of letting others down because of your migraines? (Select only **one** response.)

1☐ None of the time

2☐ A little bit of the time

3☐ Some of the time

4☐ A good bit of the time

5☐ Most of the time

6☐ All of the time

Appendix D. Patient Global Impression of Change Scale (PGIC)

Since beginning treatment at this clinic, how would you describe the change (if any) in ACTIVITY LIMITATIONS, SYMPTOMS, EMOTIONS, and OVERALL QUALITY OF LIFE, related to your painful condition? Please circle the number below that matches your degree of change since beginning care at this clinic for the above stated chief complaint.

No change	Almost the same	A little better	Somewhat better	Moderately better	Better	A great deal better
1	2	3	4	5	6	7

Explanation:

1 = No change (or condition has got worse)

2 = Almost the same, hardly any change at all

3 = A little better, but no noticeable change

4 = Somewhat better, but the change has not made any real difference

5 = Moderately better, and a slight but noticeable change

6 = Better, and a definite improvement that has made a real and worthwhile difference

7 = A great deal better, and a considerable improvement that has made all the difference

Do not write in this box - FOR OFFICE USE ONLY

NOTE TO HEALTH CARE PROVIDER

A significant, favorable change is a score of 5- 7

No significant change is a 1-4 response.

Note, this is a dichotomous scale (5-7 = yes; 1-4 = no).

A 2-point change is significant from their last reported score.

Reference: Hurst H, Bolton J. Assessing the clinical significance of change scores recorded on subjective outcome measures. Journal of Manipulative Physiological Therapeutics (IMPT) 2004;27:26-35.

Appendix E. Acupuncture Expectancy Scale (AES)

Every individual may have different expectation for the effects of acupuncture. If we use the following sentences to describe your expectation of acupuncture's effect on your illness/symptom after the entire course of acupuncture therapy, how much do you agree? For each statement, please choose the closest answer.

1. My illness will improve a lot.

1 (Not at All Agree) 2 (A Little Agree) 3 (Moderately Agree)
4 (Mostly Agree) 5 (Completely Agree)

2. I will be able to cope with my illness better.

1 (Not at All Agree) 2 (A Little Agree) 3 (Moderately Agree)
4 (Mostly Agree) 5 (Completely Agree)

3. The symptoms of my illness will disappear.

1 (Not at All Agree) 2 (A Little Agree) 3 (Moderately Agree)
4 (Mostly Agree) 5 (Completely Agree)

4. My energy level will increase

1 (Not at All Agree) 2 (A Little Agree) 3 (Moderately Agree)
4 (Mostly Agree) 5 (Completely Agree)

Appendix F. Blinding Questionnaire

Do you think which kind of treatment group you have participated in the past weeks?

☐ Real acupuncture group

☐ Sham acupuncture group

☐ Did not know
