# Supplementary material for: Acupuncture for Migraine Without Aura and Connection-Based Efficacy Prediction: A Randomized Clinical Trial
Source: JAMA Netw Open. 2026 Jan 27;9(1):e2555454. doi: 10.1001/jamanetworkopen.2025.55454 (PMC12848631; doi:10.1001/jamanetworkopen.2025.55454)
Supplement: Supplement 2. — Study Protocol [file jamanetwopen-e2555454-s002.pdf]

SUPPLEMENT 2: Study Protocol

Acupuncture for Migraine Without Aura and Connection-Based Efficacy Prediction: A Randomized Clinical Trial

Contents

Study Protocol.. 2

Acupuncture for Migraine Without Aura and Connection-Based Efficacy Prediction: A Randomized Clinical Trial

STUDY PROTOCOL

Beijing Hospital of Traditional Chinese Medicine, Capital Medical University, Beijing, China

Principal Investigator

Lu Liu, MD

Version: 1.0

30 June 2020

SIGNATURE PAGE

Declaration of Principal Investigator

Title: Acupuncture for Migraine Without Aura and Connection-Based Efficacy Prediction: A Randomized Clinical Trial

Protocol version: 1.0, dated 30 June 2020

This study protocol was subjected to critical review. The information it contains is consistent with current knowledge of the risks and benefits of the treatments, as well as with the moral, ethical, and scientific principles governing clinical research as set out in the Declaration of Helsinki, and the International Council for Harmonization Guidelines on Good Clinical Practice.

Principal Investigator

Lu Liu, MD

Beijing Hospital of Traditional Chinese Medicine ____________ ______________

Capital Medical University Date Signature

SYNOPSIS

**Title:**	**Acupuncture for Migraine Without Aura and Connection-Based Efficacy Prediction: A Randomized Clinical Trial**
**Primary Objective:**	To evaluate the efficacy of acupuncture for migraine and predict its efficacy from brain connectome data using machine learning.
**Study Design:**	A single-blinded, randomized controlled trial is used to determine the effectiveness of acupuncture in dealing with migraine and to predict how well it works by analyzing brain connectome data using machine learning.
**Patient Population:**	Patients aged between 18 and 65 years meet the diagnostic criteria for migraine without aura (MWoA) in the International Classification of Headache Disorders 3rd Edition (ICHD-3), have had at least two migraine attacks in the past 4 weeks, and have a history of migraine for at least 1 year.
**Inclusion/Exclusion Criteria:**	Inclusion criteria:<br><br>Patients were eligible to be included in the study only if they met all of the following criteria at screening:<br><br>• be male or female, aged between 18 and 65 years；<br><br>• have at least two migraine attacks in the last 4 weeks;<br><br>• have a history of migraine for at least one year;<br><br>• right handedness;<br><br>• diagnosis of MWoA based on the ICHD-3:<br><br>A. At least five attacks fulfilling criteria B–D<br><br>B. Headache attacks lasting 4–72 hours (when untreated or unsuccessfully treated)<br><br>C. Headache has at least two of the following four characteristics:<br><br>1. unilateral location

	2. pulsating quality<br><br>3. moderate or severe pain intensity<br><br>4. aggravation by or causing avoidance of routine physical activity (e.g., walking or climbing stairs)<br><br>D. During headache at least one of the following:<br><br>1. nausea and/or vomiting<br><br>2. photophobia and phonophobia<br><br>E. Not better accounted for by another ICHD-3 diagnosis.<br><br>• able to complete a headache diary;<br><br>• were able and willing to follow study procedure and give signed informed consent.<br><br>Exclusion criteria:<br><br>Patients were excluded from study enrolment if they met any of the following criteria at screening:<br><br>Diagnostics Assessments:<br><br>• diagnoses of new daily persistent headache, tension-type headache, trigeminal autonomic cephalalgia, or painful cranial neuropathy as defined by the ICHD-3;<br><br>Prior/Concomitant Therapy:<br><br>• use of migraine preventive medications or prior experience with acupuncture or migraine devices within the past 3 months:<br><br>a. Beta blockers: metoprolol, propranolol, timolol, atenolol, nadolol, nebivolol, pindolol, or bisoprolol<br><br>b. Anticonvulsants: topiramate, carbamazepine, or gabapentin

	c. Tricyclic antidepressants (e.g., amitriptyline, nortriptyline, or protriptyline)<br><br>d. Calcium channel blockers: flunarizine or verapamil<br><br>e. ACEI/ARB: candesartan or lisinopril<br><br>f. Onabotulinumtoxin A<br><br>g. Valproate<br><br>•have used opioid-containing treatments on more than 4 days during the screening period;<br><br>• have used interventions or devices for migraine, during 3 months before screening. Interventions or devices for migraine are listed as: acupuncture, occipital stimulator, nerve blocks and transcranial magnetic stimulation;<br><br>Medical Conditions:<br><br>• clinically significant hematological, cardiac, renal, endocrine, pulmonary, gastrointestinal, genitourinary, neurological, hepatic, or ocular disease that, in the opinion of the investigator, could jeopardize or would compromise the patient's ability to participate in the study;<br><br>• evidence or medical history of clinically significant psychiatric issues that, in the opinion of the investigator, could jeopardize or would compromise the patient's ability to participate in this study including major depression, panic disorder, or generalized anxiety disorder, any suicide attempt in the past or suicidal ideation with a specific plan the past two years prior to screening or current suicidal ideation;<br><br>• contraindications to magnetic resonance imaging (MRI) (e.g., claustrophobia, cardiac pacemaker, or other metallic implants);<br><br>• pregnant or lactating female patients or female patients who planned to become pregnant during the study;<br><br>• history of alcohol or drug abuse/dependence during the 1

	years prior to screening;
**Randomization:**	Eligible participants were randomly assigned in a 1:1 ratio to either the real acupuncture (RA) or shame acupuncture (SA) group using an interactive web-based response system. Randomization was performed using a computer-generated code with a fixed block size of 4.
**Primary outcome:**	Change from baseline in the monthly migraine days (MMDs) during weeks 1–4
**Study Duration:**	The study design includes a screening period (3-14 days), a 4-week baseline period, and a 4-week treatment period.
**Sample Size:**	According to previous studies, during the first to fourth cycles, the reduction of MMDs after treatment was 2.2±2.0 in the RA group and 1.6±3.0 in the SA group. To detect the difference between the two groups with a power of 80% at an alpha level of 0.05 and considering a 10% dropout rate, the sample size for randomization was determined to be 120 participants (60 in each treatment group).
**Statistical Methods:**	Variables were first assessed for distributional characteristics. For outcomes that departed from normality, comparisons between the real acupuncture and sham acupuncture groups were performed using the Wilcoxon rank-sum test (Mann-Whitney U), and the Hodges-Lehmann estimate was used as the point estimate of the median difference; the Hodges-Lehmann estimate and its 95% confidence interval (CI) were derived using the Wilcoxon test. All tests were 2-sided with $\alpha = 0.05$. Results are presented as medians and interquartile range (IQRs; 25th-75th percentiles) for each group, with between-group differences given as the Hodges-Lehmann estimates and 95% CIs along with 2-sided *P* values. For outcomes that were normally distributed, comparisons were made with independent-sample, 2-tailed t tests. Results were reported as mean changes with standard error (SEs) and 95% CIs, and between-group differences as mean differences with 95% CIs and 2-sided P values. The proportion of participants with 50% or greater reduction in MMDs was assessed using a logistic regression model.

TABLE OF CONTENTS

SIGNATURE PAGE ---------- **1**
SYNOPSIS ---------- **2**
LIST OF ABBREVIATIONS ---------- **6**
1. INTRODUCTION ---------- **8**
1.1 Background ---------- 8
1.1.1 Overview ---------- 8
1.1.2 Nonclinical Data ---------- 9
1.1.3 Clinical Data ---------- 9
1.2 Rationale for the Study ---------- 9
2. OBJECTIVES AND OUTCOMES ---------- **11**
3. STUDY DESIGN ---------- **12**
3.1 Overview of the Study Design ---------- 12
3.2 Rationale for the Study Design ---------- 14
3.3 Justification of Study Design ---------- 14
3.4 Randomization and Blinding ---------- 15
3.4.1 Randomization ---------- 15
3.4.2 Blinding/Unblinding ---------- 15
3.5 Withdrawal from Study ---------- 15
3.6 Lost to Follow-up ---------- 15
4. STUDY POPULATION ---------- **16**
4.1 Participant Recruitment ---------- 16
4.2 Inclusion Criteria ---------- 16
4.3 Exclusion Criteria ---------- 16
5. INTERVENTION ---------- **17**
5.1 RA group ---------- 19
5.2 SA group ---------- 19
6. CONCOMITANT MEDICATIONS ---------- **19**
7. STUDY VISIT PLAN ---------- **20**
7.1 Screening Period ---------- 20
7.2 Baseline Period ---------- 20
7.3 Treatment Period ---------- 21
8. ASSESSMENT ---------- **21**
8.1 Screening and Baseline Procedures and Assessments ---------- 21
8.1.1 Demographics and Baseline Characteristics ---------- 21
8.2 Efficacy Assessment ---------- 22
8.2.1 Use of Clinical Outcome Assessment ---------- 22
8.2.2 Headache Diary ---------- 22
8.2.3 Headache Impact Test-6 (HIT-6) ---------- 23
8.2.4 Migraine-Specific Quality of Life (MSQOL) ---------- 23
8.2.5 Acupuncture Expectancy Scale (AES) ---------- 23
8.2.6 Patient Global Impression of Change Scale (PGIC) ---------- 23
8.2.7 Blinding assessment ---------- 24
8.3 Order of Assessments ---------- 24

8.4 Treatment Compliance ---------- 25
9. ADVERSE EVENTS---------- 25
9.1 Definitions of Adverse Event ---------- 25
9.1.1 Adverse Event Definitions ---------- 25
9.1.2 Serious Adverse Event Definitions ---------- 26
9.1.3 AE Assessment Definitions ---------- 26
9.2 Recording of Adverse Event ---------- 27
9.3 Reporting of Serious Adverse Event---------- 28
9.4 Treatment and Follow-up Visits of Adverse Events ---------- 28
9.5 Pregnancy---------- 29
9.6 Management of Reactions to Treatment ---------- 29
10. ETHICS ---------- 30
10.1 Ethical Rationale---------- 30
10.2 Informed Consent---------- 30
10.3 Personal Data Protection ---------- 31
10.4 Research Ethics Committees ---------- 31
11. DATA HANDING AND RECORD KEEPING ---------- 32
11.1 Data Collection---------- 32
11.1.1Electronic Case Report Forms (eCRFs) ---------- 32
11.1.2 Patient Binders---------- 32
11.2 Database Management and Quality Control ---------- 32
11.3 Retention of Study Documents---------- 33
12. STATISTICAL METHOLOGY---------- 33
12.1 Analysis Sets ---------- 33
12.1.1 Intention-to-Treat Analysis Set ---------- 33
12.1.2 Per-Protocol (PP) Analysis Set ---------- 34
12.1.3 Safety Analysis Set ---------- 34
12.2 Sample Size and Power---------- 34
12.3 Descriptive Statistics---------- 34
12.4 Participants Disposition ---------- 34
12.5 Demographics and Baseline Characteristics---------- 34
12.6 Recent and Concomitant Medication/Therapy ---------- 34
12.7 Efficacy Analyses---------- 35
12.7.1 General Efficacy Analysis Methodology ---------- 35
12.7.2 Primary Analysis of the Primary Outcome ---------- 35
12.7.3 Analysis of the Secondary Outcomes ---------- 35
12.8 Safety Analyses ---------- 35
12.8.1 Acupuncture---------- 35
12.8.2 Adverse Events ---------- 35
13. MONITORING PROCEDURES ---------- 36
13.1 Procedures for Monitoring Participant Compliance---------- 36
13.2 Study Monitoring---------- 36
14.STUDY DISCONTINUATION---------- 36

15. STUDY ORGANISATION ---------- 37
15.1 Steering Committee ---------- 37
15.2 Executive Committee ---------- 37
15.3 Data Monitoring Committee ---------- 37
16. PUBLICATIONS ---------- 37
17. FUNDING ---------- 38
18. REFERENCES ---------- 38
APPENDIX ---------- 40
Appendix A. Concomitant Treatments: Disallowed or Allowed with Restrictions ---------- 40
Appendix B. Headache Diary ---------- 42
Appendix C. Headache Impact Test-6 (HIT-6) ---------- 49
Appendix D.Migraine-Specific Quality Of Life Questionnaire (MSQ) (VERSION 2.1) 51
Appendix E. Patient Global Impression of Change Scale (PGIC) ---------- 56
Appendix F. Acupuncture Expectancy Scale (AES) ---------- 57
Appendix G. Blinding Questionnaire ---------- 58

LIST OF ABBREVIATIONS

Abbreviation	Definition
ACEI	Angiotensin-Converting Enzyme Inhibitor
AEs	Adverse events
AES	Acupuncture Expectancy Scale
ARB	Angiotensin Receptor Blocker
CGRP	Calcitonin gene related peptide
CIs	Confidence intervals
CPM	Connectome-based Predictive Modeling
CRF	Case Report Form
DMN	Default Mode Network
HIT-6	Headache Impact Test-6
ICHD-3	International Classification of Headache Disorders 3 version
ITT	Intention-to-Treat
LSM	Least-Squares Mean
MMDs	Monthly migraine days
MHDs	Monthly headache days
MRI	Magnetic Resonance Imaging
MSQoL	Migraine-Specific Quality of Life
MWoA	Migraine Without Aura
PGIC	Patient Global Impression of Change Scale
PP	Per-Protocol
RA	Real Acupuncture
RCTs	Randomized controlled trials
SA	Sham Acupuncture
SAE	Serious adverse event
SC	Subcortical-Cerebellum
SE	Standard error

SD	Standard deviation
SOP	Standard operating procedures
VAS	Visual Analog Scale
WHO	World Health Organization

1. INTRODUCTION

1.1 Background

1.1.1 Overview

Migraine without aura (MWoA) is a prevalent neurological disorder affecting approximately 14% of the global population[1]. It is characterized by attacks of headache and associated symptoms (such as nausea, photophobia, or phonophobia), which can lead to significant impairment in quality of life. The pathophysiology of migraine is complex, with clinical and laboratory evidence suggesting that vulnerability to migraine can be genetic or acquired. Individual migraine attacks may be triggered by a disruption of homeostatic function resulting in a cascade of effects including activation of a neuronal phenomenon known as cortical spreading depression, central and peripheral sensitization, and triggering of the trigeminovascular pathway. This pathway results in release of vasodilatory, pro-inflammatory, or pain producing neuropeptides such as calcitonin gene related peptide (CGRP), a recent target for pharmacotherapy[2].

The goals of migraine treatment are to relieve pain, restore function, and reduce headache frequency. Pharmacological interventions for the treatment of migraine include acute (symptomatic) treatments and daily preventive medications. Currently available pharmacological interventions for preventive treatment of migraine include beta blockers, anticonvulsants, tricyclic antidepressants, calcium channel blockers, angiotensin-converting enzyme inhibitor (ACEI)/angiotensin receptor blocker (ARB), onabotulinumtoxin A, and valproate. While pharmacological interventions remain first-line treatments, nearly 30% of patients demonstrate inadequate response to conventional medications, often due to limited efficacy or intolerable side effects[3,4].

Acupuncture is a developing non-pharmaceutical therapy for migraine prevention and treatment, and has been listed by the World Health Organization (WHO) as a recommended therapy for migraine. A Cochrane review suggested that acupuncture may be at least similarly effective as prophylactic drugs, and it also suggested that acupuncture reduced migraine frequency significantly more than drug prophylaxis after treatment[5].

Meanwhile, mounting evidence suggests that migraine-related pain is manifested as distinct alterations in the whole-brain functional connectivity organization, particularly involving the Default Mode Network (DMN), Subcortical-Cerebellum (SC), and Motor networks. These connectivity patterns can not only serve as diagnostic biomarkers but also potentially predict treatment responsiveness[6-8]. Based on this neurobiological framework, we hypothesized that comprehensive connectome analysis could overcome the limitations of conventional regional research methods to predict the efficacy of acupuncture in migraine treatment.

Methodologically, we chose the connectome-based predictive modeling (CPM). It employs a comprehensive data-driven framework to identify whole-brain connectivity patterns that best predict clinical outcomes[9]. This supervised learning approach objectively extracts predictive features without the need for predefined assumptions, while capturing complex distributed network interactions that often elude traditional regional analyses. Crucially, CPM can generate individualized predictions based on baseline connectivity profiles, offering clinically actionable insights.

1.1.2 Nonclinical Data

The trigger factors of migraine are multifaceted, including environmental factors, hormonal changes, medications, and lifestyle factors, as well as a strong component of genetics, which can contribute to neuroinflammation and lead to neuronal sensitization[10-12]. Animal studies have suggested that acupuncture can decrease related vasoconstrictive neurotransmitters and neuropeptides (CGRP, substance P, and pituitary adenylate cyclase-activating polypeptide)[13], which are all considered to associate with neuroinflammation in the pathogenesis of migraine. In addition, recent neuroimaging studies suggest acupuncture modulates multiple pain-processing networks, including the DMN and primary somatosensory cortex.

1.1.3 Clinical Data

Both Chinese and overseas randomized controlled trials (RCTs) have paid great attention to the effect of acupuncture in treating migraine[14-16]. Our previous study on the efficacy of acupuncture for migraine showed that acupuncture was more effective than sham acupuncture in reducing the number of migraine attack days. Participants in the acupuncture group had a better remission rate and fewer migraine attack days, with a significant difference compared to the control group ($P < 0.05$)[17]. Further studies have demonstrated that acupuncture has shown continuous and clinically relevant benefits for migraine patients. A RCT indicated that compared with sham acupuncture, acupuncture could significantly reduce the frequency of migraine attacks, as well as the number of migraine attack days and the visual analogue scale (VAS) scores[15]. However, this trial did not further predict the efficacy of acupuncture in treating migraine. Therefore, we combined a randomized, single-blind RCT with a machine learning prediction model to further predict the efficacy of acupuncture in the treatment of MWoA.

1.2 Rationale for the Study

There is a proportion of participants who do not respond to or cannot tolerate existing treatments and there is a need for preventive treatments which are more effective and better tolerated than the current standard of care. For this reason, acupuncture offers a new direction for the treatment of migraine. Acupuncture has potential advantages in the following populations: people with contraindications to medications and intolerance to conventional therapeutic drugs; people who do not want to be treated with medications; and special populations, such as pregnant women and adolescents. In addition, acupuncture is less likely to lead to medication overuse headache (MOH) and can provide participants with individualized treatment plans. Therefore, this study will evaluate and further predict the efficacy

of acupuncture in this target population, and provide evidence - based treatment guidance for these participants with MWoA. The efficacy will be investigated during the 4-week treatment period, which will contribute to a long-term investigation of the efficacy of acupuncture.

2. OBJECTIVES AND OUTCOMES

Objectives	Outcomes
Primary Objectives<br>•To evaluate the efficacy of acupuncture for the MWoA participants	•Primary Outcomes<br>- Change from baseline in number of MMDs during weeks 1-4<br><br>•Secondary Outcomes<br>- ≥50% reduction in number of monthly migraine days during weeks 1-4<br>- Change from baseline in number of monthly headache days(MHDs) during weeks 1-4<br>- Change from baseline in VAS score at week 4<br>- Change from baseline in number of days with acute medication use during weeks 1-4
Secondary Objectives<br>•To evaluate the disability scores of participants<br><br><br>•To evaluate the quality of life of participants	•Secondary Outcomes<br>- Change from baseline in HIT-6 total score at week 4<br><br>- Change from baseline in MSQoL Role Function-Restrictive domain at week 4<br>- Change from baseline in MSQoL Role Function-Preventive domain at week 4<br>- Change from baseline in MSQoL Emotional Function domain at week 4

3. STUDY DESIGN

3.1 Overview of the Study Design

This study is a single-blind RCT in China. The study will comprise a screening period (3 to 14 days), a 4-week baseline period, and a 4-week treatment period.

An overview of the study is presented in **fig 1**.

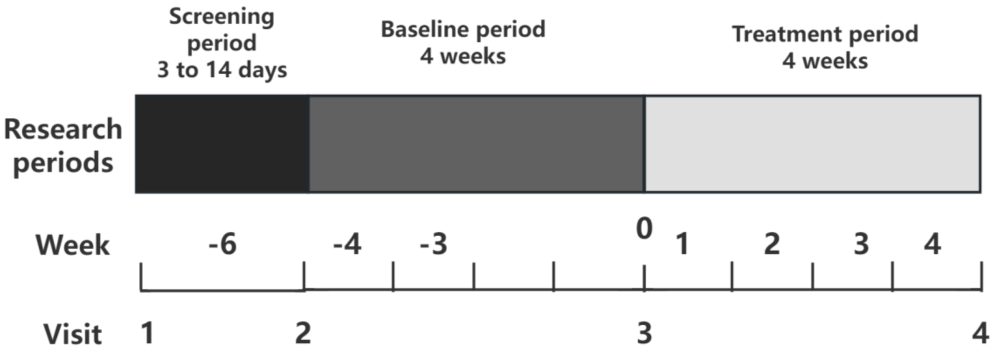

Fig 1. Study design. The study for each participant will be divided into 3 periods: a screening period (3 to 14 days), a 4-week baseline period, and a 4-week treatment period.

This study will include female and male participants, aged 18 to 65 years, suffering from migraine for at least 1 year before screening, and at least two migraine attacks in the past 4 weeks. Participants meet MWoA diagnosis of the International Classification of Headache Disorders 3 (ICHD-3) criteria[18]. The diagnosis will be prospectively confirmed via a review of headache diary recorded daily during a 4-week baseline period. See Inclusion Criteria for further details on diagnosis.

Migraine preventive medications will be prohibited during the study and 3 months prior to the start of screening. The preventive medications are listed as follows: beta blockers, anticonvulsants, tricyclic antidepressants, calcium channel blockers, ACEI/ARB, onabotulinumtoxin A, and valproate. Participants will be allowed to use acute headache medications to treat migraines as needed, with the exception of medications containing opioids, which cannot be used.

After completing the informed consent process at the end of screening period, participants will be screened for eligibility. Eligible participants will enter a 4-week baseline period. Headache information will be captured daily throughout study participation using the headache diary. After completing the baseline period, participants who have confirmed MWoA and meet all other eligibility criteria (including headache diary compliance criteria during the 4-week baseline period) will be randomly assigned in a 1:1 ratio to real acupuncture group (RA group) or sham acupuncture group (SA group).

Acupuncture will be administered 3 sessions per week (ideally every other weekday) for 4 weeks. RA involved eight acupoints with deqi sensation, while SA used sham acupoints without deqi.

Headache information will be captured daily during the entire study using a headache diary. Additional assessments of disability scores (measured by Headache Impact Test [HIT-6]), quality of life (measured by Migraine-Specific Quality of Life [MSQoL] score) will be measured at the end of baseline, treatment period (week 0, week 4).

The assessments are summarized in **table 1**. An independent data monitoring committee will oversee the data during the study.

Table 1. The schedule of enrolment, interventions, and assessments

**STUDY PERIOD**							
	**Screening**	**Baseline**	**Allocation**	**Treatment**			
**TIMEPOINT(W, week)**	-6	-4	0	1	2	3	4
**Enrolment**							
Informed consent	×						
Eligibility criteria	×	×					
Demography Characteristics		×					
Disease history of migraine		×					
Randomization			×				
**Interventions**							
RA group				⟷			
SA group				⟷			
**Assessments**							
Headache diary		⟷					
HIT-6			×				×
MSQoL			×				×
Participants' satisfaction							×
AES			×				
Assessment of blinding				×			×
Participant's compliance				×	×	×	×
Adverse events				⟷			

HIT-6=Headache Impact Test; MSQoL=Migraine-Specific Quality of Life Questionnaire; AES=Acupuncture Expectancy Scale.

The overall end of the study is defined as the last protocol-specified contact with the last participant ongoing in the study.

3.2 Rationale for the Study Design

The current study is a 4-week single-blind, randomized controlled trial to investigate whether acupuncture can lead to clinically significant improvement in MWoA patients. The primary outcome is the change from baseline in number of monthly migraine days during weeks 1-4. Outcomes evaluating disability, and quality of life are included in the study to demonstrate the impact of treatment beyond the reduction in migraine days.

MWoA is a common type of primary headache characterized by the absence of clear aura symptoms during headache attacks. According to the criteria of the 3rd edition of the ICHD-3[18], patients are required to have experienced at least 5 headache attacks that meet the diagnostic criteria. Each headache lasts from 4 to 72 hours, featuring unilateral distribution, pulsatile quality, moderate or severe pain intensity, and at least one of the following symptoms: nausea, vomiting, photophobia, or phonophobia. The diagnosis of MWoA will be confirmed through the information in the headache diaries pre - collected during the baseline period.

During the study, the participants will be asked about occurrence of aura in the headache diary. The participant will be asked about occurrence of aura based on a question; "Have you experienced aura with this headache?" Participants will be asked in the headache diary to fill in if they took any acute headache medications to treat a headache and if so, if they took this medication because they believed this was a migraine. As several medications are used both to treat non-migraine headache and migraine, the classification of a migraine-specific medication is not feasible by registering the product used alone. Furthermore, the intention is to avoid that, for example, a mild tension-type headache that was treated with paracetamol is counted as a migraine day.

Acupuncture is suggested by a review that reduces migraine frequency significantly more than shame acupuncture after treatment[19]. The acupuncture prescriptions will be developed based on the information in classical and modern literature[19], consensus with clinical experts[20], and experience from our previous study[14]. Acupoints selection will include obligatory acupoints and adjunct acupoints based on the principle of syndrome differentiation of meridians in the headache region according to the theory of Traditional Chinese Medicine.

The sample size of 120 patients for the primary outcome is based on the previous study[21], on the expected change from baseline to week 4 in the number of monthly migraine days and should provide adequate power for detection of a clinically meaningful treatment effect.

3.3 Justification of Study Design

In order to blind the treatment arms between RA and SA, acupuncture devices with similar appearances will be used for treatment.

3.4 Randomization and Blinding

After the baseline period, eligible participants will be randomly assigned (1:1) to either RA group or SA group using an interactive web-based response system (Beijing LNKMED Tech Co., Ltd). Randomization will be performed by a computer-generated random code with constant block size. The randomization sequence will be created by an independent biostatistician who did not participate in the study. The participants, outcome assessors, and the statistician will be blinded to treatment assignment.

3.4.1 Randomization

All enrolled participants will be informed of the study protocol and signed written informed consent before randomization. After the 4-week baseline period, participants will be randomly assigned (1:1) to either RA group or SA group using an interactive web-based response system (Beijing LNKMED Tech Co., Ltd). Randomization will be performed by a computer-generated random code with a fixed block size. The randomization sequence will be computer generated by an independent biostatistician who do not participate in the trial.

3.4.2 Blinding/Unblinding

The participants, outcome assessors, and the statistician will be blinded to treatment assignment. Unmasked personnel will include the dedicated investigator responsible for the randomization module. Because of the nature of the intervention, acupuncturists cannot be blinded, but they will be trained not to communicate with participants or outcome assessors about treatment procedures and responses.

In case of a serious adverse event (SAE) or pregnancy, or in cases when knowledge of the study treatment assignment is needed to make treatment decisions, the participants may unblind the treatment assignment as deemed necessary, mainly in emergency situations. When a blind is broken, the participant will be withdrawn from the study, and the event will be recorded onto the case report forms (CRFs). The circumstances leading to the breaking of the code should be fully documented in the investigator's study files and in the participant's source documentation. Treatment assignments should not be recorded in any study documents or source documents.

3.5 Withdrawal from Study

Withdrawal of consent occurs when a participant does not want to participate in the study anymore, does not want any form of follow-up, or does not want any further study related contacts. Participants have the right to withdraw from the study at any time without prejudice to their future treatment.

If a participant withdraws consent, the investigator must make every effort to determine the primary reason for this decision. If the withdrawal is due to adverse events (AEs), refer to the **section 9**. Participants who have withdrawn from the study cannot be included again in the study.

3.6 Lost to Follow-up

For participants whose status are unclear because they fail to appear for study visits without stating an

intention to withdraw, the investigator should make every effort to contact the participant. A participant should not be considered lost to follow-up until the end of the study.

4. STUDY POPULATION

4.1 Participant Recruitment

Trial participants with MWoA will be recruited by investigators from outpatient clinics at the Beijing Hospital of Traditional Chinese Medicine, Capital Medical University. Meanwhile, information flyers introducing the details of the trial will be posted at the outpatient clinics for greater exposure. The investigators will be notified immediately when the recruitment period comes to an end. A neurologist will make the diagnosis of MWoA on the basis of the ICHD-3.

4.2 Inclusion Criteria

Patients were eligible to be included in the study only if they met all of the following criteria at screening:

• be male or female, aged between 18 and 65 years；
• have at least two migraine attacks in the last 4 weeks;
• have a history of migraine for at least one year;
• right handedness;
• diagnosis of MWoA based on the ICHD-3:
• able to complete a headache diary;
• were able and willing to follow study procedure and give signed informed consent.

4.3 Exclusion Criteria

Patients were excluded from study enrolment if they met any of the following criteria at screening:

Diagnostics Assessments:

• diagnoses of new daily persistent headache, tension-type headache, trigeminal autonomic cephalalgia, or painful cranial neuropathy as defined by the ICHD-3;

Prior/Concomitant Therapy:

• use of migraine preventive medications or prior experience with acupuncture or migraine devices within the past 3 months:

a. Beta blockers: metoprolol, propranolol, timolol, atenolol, nadolol, nebivolol, pindolol, or bisoprolol

b. Anticonvulsants: topiramate, carbamazepine, or gabapentin

c. Tricyclic antidepressants (e.g., amitriptyline, nortriptyline, or protriptyline)

d. Calcium channel blockers: flunarizine or verapamil

e. ACEI/ARB: candesartan or lisinopril

f. Onabotulinumtoxin A

g. Valproate

• have used opioid-containing treatments on more than 4 days during the screening period;

• have used interventions or devices for migraine, during 3 months before screening. Interventions or devices for migraine are listed as: acupuncture, occipital stimulator, nerve blocks and transcranial magnetic stimulation;

Medical Conditions:

• clinically significant hematological, cardiac, renal, endocrine, pulmonary, gastrointestinal, genitourinary, neurological, hepatic, or ocular disease that, in the opinion of the investigator, could jeopardize or would compromise the patient's ability to participate in the study;

• evidence or medical history of clinically significant psychiatric issues that, in the opinion of the investigator, could jeopardize or would compromise the patient's ability to participate in this study including major depression, panic disorder, or generalized anxiety disorder, any suicide attempt in the past or suicidal ideation with a specific plan the past two years prior to screening or current suicidal ideation;

• contraindications to magnetic resonance imaging (MRI) (e.g., claustrophobia, cardiac pacemaker, or other metallic implants);

• pregnant or lactating female patients or female patients who planned to become pregnant during the study;

• history of alcohol or drug abuse/dependence during the 1 years prior to screening;

5. INTERVENTION

We chose the acupuncture prescriptions as a result of the information in classical and modern literature[5], consensus with clinical experts[20], and experience from our previous study[14]. Acupuncture treatment will be performed by two licensed acupuncturists who had at least 5 years of acupuncture experience. All the acupuncturists will be trained how to locate acupoints and non-effective acupoints, puncture and manipulate needles before the trial. Participants will be treated in a single treatment room for privacy and to avoid communication. The acupuncturists will be asked to have the least possible communication with the participants to minimize bias. There are two groups in this trial: RA group and SA group. The location and manipulations of acupoints and non-effective acupoints are shown in **table 2, table 3, and table 4**.

Participants will receive acupuncture treatment for 4 weeks and be given 30-minute sessions (3 sessions per week, ideally every other weekday).

Table 2. The real acupoints location

Acupoint	Meridian	Location
Bai Hui	GV20	The point is located at the top of the head, at the midpoint of the line connecting the apexes of both ears, corresponding to the vertex of the sagittal suture.
Feng Fu	GV16	Feng Fu is situated at the nape of the neck, in the depression immediately below the hairline, directly above the spinous process of the second cervical vertebra (C2).
Feng Chi	GB20	This point is found at the back of the neck, in the groove between the styloid process of the temporal bone and the upper edge of the trapezius muscle, approximately at the level of the C2-C3 interspinous space.
Tai Yang	EX-HN5	Tai Yang is located in the temporal region, about one inch posterior to the midpoint of the eyebrows, in the depression lateral to the orbit of the eye.
He Gu	LI4	He Gu is situated on the dorsum of the hand, between the first and second metacarpal bones, in the depression when the thumb and index finger are adducted. The point is typically marked at the highest point of the muscle belly when the hand is in a relaxed position.

Table 3. The sham acupoints location

Acupoint	Location
Sham acupoint (1)	On the medial aspect of the arm, at the anterior margin of the deltoid muscle insertion, where the deltoid meets the biceps muscle.
Sham acupoint (2)	Mid-way between the tip of the elbow and the axilla.
Sham acupoint (3)	On the ulnar side of the arm, at the mid-point between the medial epicondyle of the humerus and the ulnar side of the wrist.
Sham acupoint (4)	Along the edge of the tibia, approximately 1–2 cm lateral to and on the same horizontal level as Zusanli [ST36][a].

[a]Zusanli [ST36] is located 3 cun directly below Dubi and 1 finger-width lateral to the anterior edge of the tibia. As for the location of Dubi, when the knee is bent, it is at the knee, beneath the patella, in the depression beside the patellar ligament.

Table 4. The manipulations of the real acupoints.

Kinds of acupoint	Acupointss	Manipulation	Deqi
Du Meridian	Baihui (GV20), Fengfu(GV16)	GV20: obliquely inserted at an angle of 30-45 degree to a depth of 10-15mm.<br>GV16: Fengfu: inserted obliquely at an angle of 15-30 degrees to a depth of 10-15mm.	Twirling, lifting, and thrusting (needle manipulation) will be performed to produce deqi

Shaoyang Meridian	Fengchi(GB20), bilateral<br>Taiyang(EX-HN5), bilateral	Inserted vertically for about 10-15mm.	
Yangming Meridian	Hegu (LI4), bilateral	Inserted vertically for about 10-15mm.	

5.1 RA group

In the RA group, Baihui (GV20), Fengfu (GV16), bilateral Fengchi (GB20), bilateral Taiyang (EX-HN5), bilateral Hegu (LI4) are selected as acupoints. Sterilized, single-use needles (Hwato Needles, made in Suzhou, China) will be used for acupuncture in this trial. The number of needles will be 8 in each session for both groups. Needles of 0.30mm in diameter and 40mm in length will be used for limb acupoints, and needles of 0.25mm in diameter and 25mm in length will be used for head acupoints. All needles will be inserted 10-15mm in depth and twirling lifting, and thrusting (needle manipulation) will be performed for at least 10 seconds and repeated a total of 4 times with an interval of 10 minutes to produce a characteristic sensation known as Deqi (a sensation of soreness, numbness, distention, or heaviness that indicates effective needling).

5.2 SA group

In the SA group, the acupuncture will be conducted using the same methods as RA group but on different points, non-effective acupoints including three points on the arms and one point on the legs. And Deqi manipulations will be not used(**table 3 and fig 2**). The number of needles will be 8 in each session, which is the same as the acupuncture group.

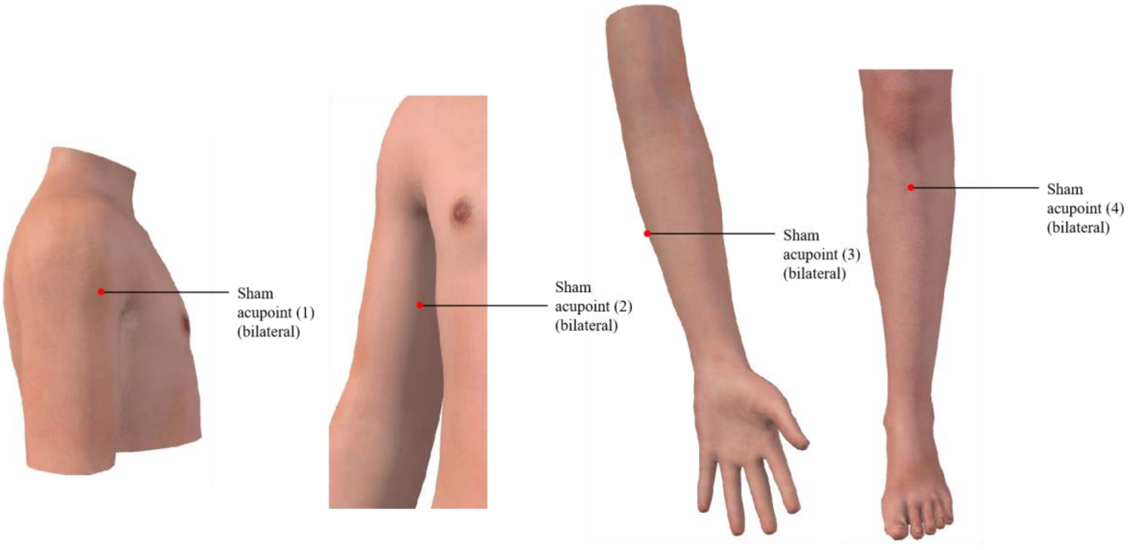

Fig 2. Location of non-effective acupoints for SA group

6. CONCOMITANT MEDICATIONS

Concomitant medication is any medication that is taken during the study up until the completion or withdrawal and during the screening period. The concomitant medications that are disallowed or allowed with restrictions during the study are summarized in **Appendix A**.

Details of all concomitant medication (including over-the-counter medications, vitamins, or herbal or nutritional supplements) taken <3 months prior to the screening period must be recorded in the CRFs at the beginning of screening period.

All concomitant medications taken during the study, must be recorded with indication, daily dose, and start and stop dates of administration. All patients will be questioned about concomitant medication use at each visit.

Any changes (including reason for changes) in concomitant medication must be recorded at each subsequent visit.

Details of all migraine preventive treatment (prescription and over-the-counter) prior to the screening period must be recorded in the CRFs at the screening period.

For any concomitant medication for which the dose has been increased due to worsening of a concurrent disorder after enrolment in the study, the worsening of the disorder must be recorded as an AE. For any concomitant medication initiated due to a new disorder after enrolment in the study, the disorder must be recorded as an AE.

7. STUDY VISIT PLAN

7.1 Screening Period

The screening period will be conducted 3 to 14 days before the baseline period. Signed informed consent will be obtained at the end of the screening period. Participant will be preliminary screened for inclusion and exclusion criteria during the screening period as well.

7.2 Baseline Period

At the beginning of a 4-week baseline period, the demography characteristics (age, sex) and disease history of migraine should be recorded.

The participant must be assisted with the provisioning and training of the headache diary at the beginning of the baseline period. The headache diary will be recorded daily during the baseline period. See **section 8.2.2** for further details on headache diary. Participants compliance will be checked and at least 85% entries with the headache diary should be attained. And an inclusion and exclusion criteria review based on headache diary must be done at the end of baseline.

Each participant will also be provided with a participant identification card that states the participant identification number, the investigator's name, and an emergency telephone number providing 24-hour service. The participant identification card should be returned to the investigator upon completion of the participant's participation in the study.

At the allocation, participants will be randomly assigned (1:1) to either RA or SA group. Additional assessments of disability scores (measured by HIT-6), and quality of life (measured by MSQoL) will be measured at the end of baseline.

7.3 Treatment Period

Participants will receive real or shame acupuncture for 12 sessions (3 sessions per week, ideally every other weekday for 4 weeks) during the treatment period.

Additional assessments of disability scores (measured by HIT-6), and quality of life (measured by MSQoL) will also be accessed at the end of treatment period. The AEs will be monitored and recorded during the treatment period.

The patient satisfaction will be measured by global impression of change scale (PGIC) at the end of treatment period. The blinding of patients will be determined by asking them which group they participate with one of the following options as the answer "Think in RA group", "Think in SA group" or "Did not know", which will be conducted within five minutes after their treatments at week 1 and week 4. Participants compliance will be checked every week during the treatment period and at least 50% entries with the headache diary should be attained.

Headache diary will be recorded daily, and AEs will be monitored and recorded during the follow-up period.

8. ASSESSMENT

8.1 Screening and Baseline Procedures and Assessments

8.1.1 Demographics and Baseline Characteristics

Prior to enrolling a patient in the study, the investigator must ascertain that the patient meets the selection criteria. The following assessments will be performed after the Informed Consent Form has been signed:

- Demographics (age, sex)
- Prior migraine treatment history for review and documentation of previous treatment of different migraine preventive medications
- Other recent medication
- Disease-specific history

- Relevant history (medical, psychiatric, neurological)
- Substance use
- Signs and symptoms present at screening and/or baseline (before treatment start)

8.2 Efficacy Assessment

8.2.1 Use of Clinical Outcome Assessment

The clinical outcome assessment and guidance will be given to the patients on how to complete them. The clinical outcome assessment will be administered in Chinese. All the following questionnaires are compared with the original text, and the Chinese version was confirmed without any addition or subtraction. The expression of each item is simple and easy to understand, and conforms to the expression habits of Chinese culture.

The following clinical outcome assessment will be used:

Headache diary – to assess daily headache and migraine variables, that is the number of hours with headache, presence of associated symptoms, and use of acute headache medications start and stop dates, headache severity (**Appendix B**)

HIT-6 – to assess headache-related disability (**Appendix C**)

MSQoL – to measure the quality of life (**Appendix D**)

PGIC – to assess patient satisfaction (**Appendix E**)

AES – to measure the expectation of improvement of illness (symptom), enhanced coping, increased vitality, and symptom alleviation due to acupuncture therapy **(Appendix F)**

Blinding assessment – to test the success of blinding (**Appendix G**)

8.2.2 Headache Diary

The patient will be instructed to complete a headache diary daily from baseline until completion/withdrawal. Eligible patients will receive comprehensive training from investigators on the use of the headache diary. Investigators will also instruct patients on the requirement for timely and daily completion of the headache diary.

The content of the headache diary is developed on key symptoms and characteristics as mentioned in the definition of migraine. The day of headache diary distribution will start the 28-day headache diary baseline period during which the patient will record daily information regarding headache characteristics, severity, length, and intake of headache/migraine medication. Headache and migraine items will be assessed with a yes/no response; and severity will be rated as mild, moderate, or severe. A headache diary eligibility report will be used to summarize baseline migraine and headache days and headache diary compliance during the baseline period. Any patient found to be ineligible for the study during the baseline period or prior to randomization will not be randomized. On each day during the study until the completion/withdrawal, the patient will be asked to record diary data for the previous 24-hour period. For each day, the patient should

record if they experienced any headaches. For each experienced headache, the start and stop date and time will be collected. The information included in the definition of a migraine (for instance, headache severity, additional symptoms, and use of acute headache medication) will also be collected. Patients will also record whether photophobia, phonophobia, nausea, and vomiting are present, and they will record any migraine medications (name of drug, number of tablets/capsules, and the dose in milligrams per tablet/capsule) taken on each day. At least 85% compliance is needed during the baseline period and 50% during the treatment and follow-up periods. And the compliance will be assessed every four weeks.

8.2.3 Headache Impact Test-6 (HIT-6)

HIT-6 is a short, self-administered questionnaire developed as a global measure of adverse headache impact using widely measured, functionally relevant domains: pain, social and role limitations, cognitive functioning, vitality, and psychological distress. Each question is answered on a scale ranging with the following response options: 6 points (never), 8 points (rarely), 10 points (sometimes), 11 points (very often), and 13 points (always). The scores for all glossary-six items are summed to produce a total HIT-6 score (range: 36–78), interpreted as little or no impact (≤49), some impact (50–55), substantial impact (56–59), and severe impact (60–78) due to headache with higher scores indicating greater impact and decreased scores consistent with improvement. If 1 or more items are missing, then the total score is missing.

8.2.4 Migraine-Specific Quality of Life (MSQoL)

The self-administered, migraine-specific, 14-item MSQoL questionnaire is designed to measure how migraines affect and/or limit daily functioning across 3 domains: Role Function-Restrictive domain comprising 7 items assessing how migraines limit one’s daily social and work-related activities; Role Function-Preventive domain comprising 4 items assessing how migraines prevent these activities, and Emotional Function domain comprising 3 items assessing the emotions associated with migraines. Items are rated on a six-point scale (none of the time, a little bit of the time, some of the time, a good bit of the time, most of the time, and all of the time). The raw dimension scores are computed as a sum of item responses and rescaled to a 0 to 100 scale such that higher scores indicate better quality of life and improvement.

8.2.5 Acupuncture Expectancy Scale (AES)

The AES consists of four items measuring the expectation of improvement of illness (symptom), enhanced coping, increased vitality, and symptom alleviation due to acupuncture therapy. Patients will be asked to rate from 1 to 5 on a five-point Likert scale, with 1 indicating “Not at all agree” and 5 indicating “Completely agree” with the expected improvement as result of acupuncture. The score of instrument ranges from 4 to 20 out of a possible 4 to 20, with higher scores indicating greater expectancy.

8.2.6 Patient Global Impression of Change Scale (PGIC)

The PGIC scale is a validated generic tool for assessment of patient satisfaction. Patients will rate how they describe the change (if any) that their migraine/headaches have had in their general quality of life and health status since beginning the treatment in this study on a 7-point scale where 1=no change (or condition got worse); 2=almost the same, hardly any change at all; 3=a little better, but no noticeable change; 4=somewhat better, but the change has not made any real difference; 5=moderately better, and a slight but noticeable change; 6=better, and a definite improvement that has made a real and worthwhile difference; and 7=a great deal better, and a considerable improvement that has made all the difference.

8.2.7 Blinding assessment

To test the success of blinding, within 5 minutes after treatment at week 1 and at week 4, participants will be told that there are two kinds of treatment groups: “RA group” and “SA group”, and they will be randomly assigned to either group at 50% chance respectively. Participants will then be asked to answer the question “Do you think which kind of treatment group you have participated in during the past weeks?” The participants will be able to choose one of the following options as the answer: “Think in RA group”, “Think in SA group” or “Did not know”.

The Bang blinding indices will be used to assess the success of blinding. The Bang blinding index for each group represents the proportion of participants making a correct treatment guess beyond chance; 0 represents perfect blinding, a positive index indicates a correct guess, and a negative index indicates a guess in the opposite direction.

8.3 Order of Assessments

The assessments should preferably be administered in the following order:

No study related activities must be conducted until after the applicable Informed Consent Form is signed and the schedule of assessments is summarized in the **table 1**.

Screening period:

- Informed consent Form will be signed
- Eligibility criteria

Baseline period:

- Eligibility criteria
- Demography Characteristics
- Disease history of MWoA
- Patients must complete the daily headache diary entries prior to treatment.
- The clinical outcome assessment completed in the clinic must be done before the treatment, including HIT-6, MSQoL and AES.

Treatment period:

• Patients must complete the daily headache diary entries during treatment period.

• The clinical outcome assessment completed in the clinic must be done during treatment period, including HIT-6, MSQoL.

• PGIC will be assessed at week 4.

• The assessment of blinding will be conducted at week 1 and week 4.

• Participant’s compliance will be ensured every week.

8.4 Treatment Compliance

The performance of study treatment by the participants will be monitored by the investigator. Treatment compliance will be determined by investigator who maintained the records for acupuncture sessions. Treatment compliance verification should be documented in the CRFs. If compliance is considered poor, the participants should be counselled on the importance of performing the study treatment.

Acupuncture compliance will be assessed based on the percentage of participants who have received at least 10 sessions (12 sessions in total, compliance rates ≥ 80%) of acupuncture.

9. ADVERSE EVENTS

9.1 Definitions of Adverse Event

The study has been designed to minimize risks. Participants will be monitored closely for AEs with frequent visits. AEs will be managed by the investigator depending on the nature of the event. Examination and close follow-up of parameters capturing participants’ overall health will be collected on CRFs. These will be completed at every study visit, and data will be compiled into a prespecified format and reviewed by the investigator.

9.1.1 Adverse Event Definitions

AE is any untoward medical occurrence in a patient or clinical study patient administered a medical intervention and which does not necessarily have a causal relationship with this treatment. A new condition or the worsening of a pre-existing condition will be considered an AE. Stable chronic conditions that are present before study entry and do not worsen during this study will not be considered AEs.

An AE can therefore be any unfavorable and unintended sign, symptom, or disease temporally associated with the use of a medical intervention, regardless of whether it is considered related to the medical intervention.

Accordingly, an AE can include any of the following:

• All AEs associated with acupuncture include broken needle, needle phobia, needling pain after treatment, numbness, intense pricking, pricking lasting more than half an hour (no matter how intense it is) after acupuncture, subcutaneous hematoma, bleeding, infection, abscess formation at the needling site, other discomfort induced by acupuncture (such as fatigue, drowsiness, nausea, vomiting, palpitation, dizziness, pain, loss of appetite, insomnia, etc.), and aggravation of existing symptoms, etc.

• intercurrent illnesses

• physical injuries

• events possibly related to concomitant medication

• significant worsening (change in nature, severity, or frequency) of the disease under study or other pre-existing conditions (Note: A condition recorded as pre-existing that is intermittently symptomatic and that occurs during this study should be recorded as an AE.)

• events occurring during diagnostic procedures of this study or during any follow-up period of this study

• laboratory or diagnostic test abnormalities that result in the withdrawal of the patient from the study, are associated with clinical signs and symptoms or a SAE, require medical treatment or further diagnostic work-up, or are considered by the investigator to be clinically significant (Note: Abnormal laboratory test results at the baseline period that preclude a patient from entering the study or receiving treatment are not considered AEs.)

9.1.2 Serious Adverse Event Definitions

SAE is any adverse event that:

• results in death

• is life-threatening (this refers to an event in which the patient was at risk of death at the time of the event; it does not refer to an event that hypothetically might have caused death had it been more severe)

• requires inpatient hospitalization or prolongation of existing hospitalization

• results in persistent or significant disability/incapacity

• is a congenital anomaly/birth defect

• is medically important (this refers to an event that may not be immediately life-threatening or result in death or hospitalization, but may jeopardize the patient or may require intervention to prevent any of the SAEs defined above)

An AE that does not meet any of the criteria for seriousness listed above will be regarded as a non-serious AE.

9.1.3 AE Assessment Definitions

Assessment of Intensity

The investigator must assess the intensity of the AE using the following definitions, and record it on the Adverse Event Form:

• Mild – the AE causes minimal discomfort and does not interfere in a significant manner with the patient's normal activities.

• Moderate – the AE is sufficiently uncomfortable to produce some impairment of the patient's normal activities.

• Severe – the AE is incapacitating, preventing the patient from participating in the patient's normal activities.

Assessment of Causal Relationship

The investigator must assess the causal relationship between the AE and the treatment using the following definitions, and record it on the Adverse Event Form and the Serious Adverse Event Form (if applicable):

• Probable – the AE has a strong temporal relationship to the treatment or recurs on rechallenge, and another etiology is unlikely or significantly less likely.

• Possible – the AE has a suggestive temporal relationship to the treatment, and an alternative etiology is equally or less likely.

• Not related – the AE has no temporal relationship to the treatment or is due to underlying/concurrent disorder or effect of another therapy (that is, there is no causal relationship between the treatment and the AE).

An AE is considered causally related to the use of the treatment when the causality assessment is probable or possible.

Assessment of Outcome

The investigator must assess the outcome of the AE using the following definitions, and record it on the Adverse Event Form and the Serious Adverse Event Form (if applicable):

• Recovered – the patient has recovered completely, and no symptoms remain.

• Recovering – the patient's condition is improving, but symptoms still remain.

• Recovered with sequelae – the patient has recovered, but some symptoms remain (for example, the patient had a stroke and is functioning normally, but has some motor impairment).

• Not recovered – the patient's condition has not improved, and the symptoms are unchanged (for example, an atrial fibrillation has become chronic).

• Death.

9.2 Recording of Adverse Event

AEs must be recorded on an Adverse Event Form through the entire study. The investigator must provide information on the AE, preferably with a diagnosis, or at least with signs and symptoms; start and stop dates (and start and stop time if the AE lasts less than 24 hours); intensity; causal relationship to the treatment; action taken; and outcome. If the AE is not related to the treatment, an alternative aetiology must be recorded, if available. If the intensity changes during the course of the AE, this must be recorded on the Adverse Event Form. At each contact with the patient, the

investigator must question the patient about AEs by asking an open-ended question such as, “Have you had any unusual symptoms or medical problems since the last visit? If yes, please describe.”

If the AE is serious, this must be indicated on the Adverse Event Form. Furthermore, the investigator must fill out a Serious Adverse Event Form and report the SAE to the principal investigator immediately (within 24 hours) after becoming aware of it (see section 9.3).

If individual AEs are later linked to a specific diagnosis, the diagnosis should be reported and linked to the previously reported AEs.

The investigator does not need to actively monitor patients for AEs once the study has ended. SAEs occurring after the defined study period should be reported to the principal investigator if the investigator becomes aware of them, following the procedures described in section 9.3.

9.3 Reporting of Serious Adverse Event

The investigator must report SAEs to the principal investigator immediately (within 24 hours) after becoming aware of them by completing a Serious Adverse Event Form. The initial Serious Adverse Event Form must contain as much information as possible and, if more information about the patient’s condition becomes available, the Serious Adverse Event Form must be updated with the additional information.

It is the investigator’s responsibility to be familiar with requirements regarding reporting SAEs to the Research Ethical Committee and to act accordingly. The principal investigator will assume responsibility for reporting SUSARs (suspected, unexpected, serious, adverse reactions) to the authorities in accordance with requirements and Research Ethical Committee. The principal investigator will assess the expectedness of SAEs and inform the investigators about SUSARs in the blinded SUSAR listings.

9.4 Treatment Adverse Events

Each patient is free to withdraw from the study at any time. The investigator can assess the subject's deterioration or AEs and determine whether the withdrawal from the trial is in the subject's best interest. Should a patient decide to withdraw after the administration of treatment, or should the investigator decide to withdraw the patient, all efforts will be made to complete and report all observations up to the time of withdrawal. A complete final evaluation at the time of the patient’s withdrawal should be made and an explanation given as to why the patient is withdrawing or being withdrawn from the study. And patients with AEs must be treated in accordance with usual clinical practice at the discretion of the investigator.

Non-serious AEs must be followed up until resolution or the completion/withdrawal visit, whichever comes first. At the completion/withdrawal visit, information on new AEs, if any, and stop dates for previously reported AEs must be recorded. SAEs that are spontaneously reported by a patient to the investigator after the completion/withdrawal visit must be handled in the same manner as SAEs that occur during the study. These SAEs will be recorded in the CRFs. The investigator must follow up on all SAEs until the event has resolved or stabilized, until the patient is referred to the care of a health care professional, or until a determination of a cause unrelated to the treatment or study procedure is made, and report to the principal investigator all relevant new information using the same procedures and timelines as those for the initial Serious Adverse Event Form.

Patients with a clinically significant out-of-range clinical safety laboratory test value at the completion/withdrawal visit must be followed in accordance with usual clinical practice. If the clinically significant out-of-range clinical safety laboratory test value has not normalized or stabilized or a diagnosis or a reasonable explanation has not been established by the investigator at the completion/withdrawal visit, the investigator must decide whether further follow-up visits are required (this may include an additional medical examination and/or additional blood sampling). If further follow-up visits are made, these must be documented in the patient’s medical records and not in the CRF.

9.5 Pregnancy

Although not necessarily considered as an AE, pregnancy in a patient in the study must be recorded on an Adverse Event Form, even if no AE associated with the pregnancy has occurred. Pregnancies must be reported to the principal investigator the same as SAEs. Any female patient becoming pregnant during the study will discontinue the treatment. All patients who become pregnant will be monitored for the outcome of the pregnancy (including spontaneous or voluntary termination).

An uncomplicated pregnancy should not be reported as an SAE; hospitalization for a normal birth should not be reported as an SAE. If, however, the pregnancy is associated with an SAE, the appropriate serious criterion must be indicated on the Serious Adverse Event Form. Examples of pregnancies to be reported as SAEs (medically important) are spontaneous abortions, stillbirths, and malformations.

9.6 Management of Reactions to Treatment

A medical emergency should be treated appropriately by the investigator using proper standard of care and according to the guidelines for that emergency condition. Emergency equipment and medication for the treatment of these potential AEs must be available for immediate use. Should a medical condition arise that the investigator believes is related to the treatment, clinical judgement

should be used to provide the appropriate response, including the consideration of discontinuation of treatment.

10. ETHICS

10.1 Ethical Rationale

Acupuncture has been listed by WHO as a recommended therapy for migraine. A persistent, clinically relevant benefit of acupuncture for migraine participants has been indicated by further studies[15,22]. In the acupuncture group, the acupuncture prescriptions were developed based on the information in classical and modern literature[5], consensus with clinical experts, and experience from our previous study[20].

The participants will be fully informed about the study, including the risks and benefits of their participation in the study. Based on data from the nonclinical and clinical studies, and in combination with the cautionary measures implemented in the study design, the risks for the participants are considered well controlled and balanced with the potential benefits of the treatment.

The participants may withdraw from the study at any time, for any reason, specified or unspecified and without penalty or loss of benefits to which the participant is otherwise entitled.

In accordance with “Good Clinical Practice” guidelines of the International Conference on Harmonization, qualified medical personnel will be readily available to advise on study-related medical questions. Medical monitoring will be performed throughout the study. Safety data will be reviewed regularly by the Research Ethical Committee of Beijing Hospital of Traditional Chinese Medicine, Capital Medical University to ensure that prompt action is taken, if needed.

In accordance with Good Clinical Practice (GCP), the investigator will be responsible for all study related medical decisions.

10.2 Informed Consent

The investigator should fully inform the participant of all pertinent aspects of the study, including the written information. All written and/or oral information about the study will be provided as nontechnical as practical and understood by the participants. The participants should be given ample time and opportunity to inquire about details of the study and to decide whether or not to participate in the study.

Written informed consent will be obtained from each participant before any study-specific procedures or assessments are done and after the aims, methods, anticipated benefits, and potential hazards are explained. The participant’s willingness to participate in the study will be documented

in a consent form, which will be signed and personally dated by the participant and by the person who conducted the informed consent discussion. The investigator will keep the original consent forms, and copies will be given to the participants. It will also be explained to the participants that the participant is free to refuse entry into the study and free to withdraw from the study at any time without prejudice to future treatment. As the blood and urine sampling for clinical laboratory tests and analyses is an integral part of this study, the main Informed Consent Form covers these analyses.

The investigator must identify vulnerable participants, that is, participants whose willingness to participate in this study might be unduly influenced by the expectation, regardless of whether it is justified, of benefits associated with participation, or of a retaliatory response from senior members of a hierarchy in case of refusal to participate. Participants thus identified must be excluded from participation in the study.

The participants must be informed that persons authorized by the acupuncturist, the clinical research assistant, the statistician, and the principal investigator may view their medical records. The confidentiality of the participants will in all cases be respected.

The consent procedures described above will only be implemented if allowed by law and regulations and will only be initiated after approval by the relevant ethics committees.

10.3 Personal Data Protection

The investigator must ensure that the privacy of the participants, including their identity and all personal medical information, will be maintained at all times. In CRFs and other documents submitted to the principal investigator, participants will be identified not by their names, but by an identification code (i.e., identification number).

Personal medical information may be reviewed for the purpose of participants safety and/or verifying data in the source and transcribed onto the CRFs. This review may be conducted by the investigators. Personal medical information will always be treated as confidential.

The investigators are responsible for ensuring the privacy, health, and welfare of the participants during and after the study and must ensure that trained personnel are immediately available in the event of a medical emergency. The investigators must be familiar with the background to, and requirements of, the study and with the contents of the treatments and medications as described in the interventions.

10.4 Research Ethics Committees

Before this study starts, the protocol will be submitted to the Research Ethical Committee for

review. As required, the study will not start before the Research Ethical Committee to give written approval or a favorable opinion.

11. DATA HANDING AND RECORD KEEPING

11.1 Data Collection

11.1.1Electronic Case Report Forms (eCRFs)

CRFs will be used to collect all the data related to the study. The baseline characteristics of patients will be recorded by our investigator during the baseline period in CRFs, and the data manager will review all data. Upon the completion of the treatment, all patients' data will be completed and recorded on the original CRFs, following which the data manager will check data sets to ensure accuracy. If any inconsistencies are noted, corrections will be made according to, and marked on, the original CRFs.

The eCRFs use third party database (Beijing LNKMED Tech Company Limited) to capture data via an online system on a computer. When the investigator enters data in the eCRF (ideally during the visit or as soon as possible [<3 days] thereafter), the data will be recorded electronically in the database, and all entries and modifications to the data will be logged in an audit trail. Access to the system will only be granted after appropriate and documented training. Written instructions for using the system will be provided along with the training.

Electronic signatures will be used where signatures are required on pages and/or visits. Automated data entry checks will be implemented where appropriate; other data will be reviewed and evaluated for accuracy by the principal investigator. All entries, corrections, and changes must be made by the investigator.

11.1.2 Patient Binders

A Patient Binder will be provided for each patient. The Patient Binder contains different types of source documents, organized by visit and type. A ballpoint pen with waterproof ink must be used to enter information in the Patient Binder. The Patient Binder also contains Serious Adverse Event Fallback Forms. These forms must be used when the eCRF cannot be accessed.

11.2 Database Management and Quality Control

Quality control will be applied to ensure that all data are reliable and have been processed correctly, both during and after the trial. After the data is entered in the eCRFs, the principal investigator's designee must perform source data verification check systematically for accuracy, consistency, completeness, and reliability, including a comparison of the data in eCRFs with source documents. Data should only be included in the final analysis when checks have been satisfactorily completed. If data does not pass validation rules, data queries will be addressed to

the investigator to request clarification or correction. The investigator is obliged to respond by confirming or modifying the data questioned.

11.3 Retention of Study Documents

Principal Investigator Responsibilities

The principal investigator will have final responsibility for the processing and quality control of the data. Data management oversight will be carried out as described in the standard operating procedures (SOPs) for clinical studies. These SOPs will be reviewed by the principal investigator before the start of data management activities. The original CRFs will be archived by the principal investigator.

Investigator Responsibilities

The investigator must maintain all written and electronic records, accounts, notes, reports, and data related to the study and any additional records required to be maintained, including, but not limited to, the following: full case histories, signed informed consent forms, patient identification lists, CRFs for each patient on a per-visit basis, data results from other sources (e.g., laboratory, headache diary data), safety reports, reports of receipt, use, and disposition of the medication. The investigator will retain all records related to the study until principal investigator sends written notification that records may be destroyed.

All paper research-related documents will be maintained in a closed file cabinet. All research materials, including hard copies and digital data, will be kept on file for at least five years following publication.

Before the study has been completed, the investigator can read and entry to the eCRF. After the study has been completed, all user access to the eCRF will be revoked. And the eCRF cannot be modified again.

At the end of the study, Beijing LNKMED Tech Company Limited will be provided with all data related to the study (including CRF data, queries, and the audit trail) using a secure electronic medium. When confirmation of receipt of the data has been received from Beijing LNKMED Tech Company Limited all user access to the CRF will be revoked. If, for some reason, the data are not readable for the full retention period, the investigator may request that the data be re-sent.

12. STATISTICAL METHOLOGY

A general description of the statistical methods is outlined below. A more detailed Statistical Analysis Plan will be provided in a separate document that will be finalized prior to database lock.

12.1 Analysis Sets

12.1.1 Intention-to-Treat Analysis Set

The Intention-to-Treat (ITT) analysis set will include all randomized participants. In this population, treatment will be assigned based on the treatment to which participants are randomized, regardless of which treatment they actually received.

12.1.2 Per-Protocol (PP) Analysis Set

The per-protocol (PP) analysis set consisted of all participants who met all eligibility criteria and were randomized, complete the treatment and follow-up plan, and were without any major protocol deviations.

12.1.3 Safety Analysis Set

The safety analysis set will include all participants who receive at least one treatment. In this population, treatment will be assigned based upon the treatment participants actually receive, regardless of the treatment to which they are randomized.

12.2 Sample Size and Power

According to previous studies[21], during the first to fourth cycles, the reduction of MMDs after treatment was 2.2±2.0 in the RA group and 1.6±3.0 in the SA group. To detect the difference between the two groups with a power of 80% at an alpha level of 0.05 and considering a 10% dropout rate, the sample size for randomization was determined to be 120 participants (60 in each treatment group).

12.3 Descriptive Statistics

All summary statistics will be computed and displayed by treatment group. In general, summary statistics (count [n], mean, least-squares mean [LSM], standard deviation [SD], standard error [SE]) will be presented for continuous variables and counts and, if relevant, percentages will be presented for categorical variables.

12.4 Participants Disposition

Data from participants screened, participants screened but not randomized and reason not randomized, participants who are randomized (i.e., in the ITT set), participants randomized but not treated, participants in the safety and other analysis sets, participants who complete the study, and participants who withdraw from the study will be summarized using descriptive statistics. Data from participants who withdraw from the study will also be summarized by reason for withdrawal using descriptive statistics.

12.5 Demographics and Baseline Characteristics

Participant demographics and baseline characteristics (sex, age), disease history of MWoA and baseline efficacy variables, will be summarized by treatment group using descriptive statistics.

12.6 Recent and Concomitant Medication/Therapy

Any recent and concomitant medication/therapy will be summarized during the study. The use of

these will be summarized using descriptive statistics.

12.7 Efficacy Analyses

12.7.1 General Efficacy Analysis Methodology

The ITT set (see **section 12.1.1**) will be used for all efficacy analyses. Summaries will be presented by treatment group.

12.7.2 Primary Analysis of the Primary Outcome

Variables were first assessed for distributional characteristics. For outcomes that departed from normality, comparisons between the real acupuncture and sham acupuncture groups were performed using the Wilcoxon rank-sum test (Mann-Whitney U), and the Hodges-Lehmann estimate was used as the point estimate of the median difference; the Hodges-Lehmann estimate and its 95% confidence interval (CI) were derived using the Wilcoxon test. All tests were 2-sided with $\alpha = 0.05$. Results are presented as medians and interquartile range (IQRs; 25th-75th percentiles) for each group, with between-group differences given as the Hodges-Lehmann estimates and 95% CIs along with 2-sided P values. For outcomes that were normally distributed, comparisons were made with independent-sample, 2-tailed t tests. Results were reported as mean changes with standard error (SEs) and 95% CIs, and between-group differences as mean differences with 95% CIs and 2-sided P values.

12.7.3 Analysis of the Secondary Outcomes

The proportion of participants with 50% or greater reduction in MMDs was assessed using a logistic regression model. The analysis of continuous secondary outcomes were performed similarly to the primary efficacy outcome.

12.8 Safety Analyses

The safety population will be used for all safety analyses. Summaries will be presented by treatment group unless specified otherwise.

12.8.1 Administration of Acupuncture

Number (%) of patient receiving each session of acupuncture will be summarized using descriptive statistics by treatment group.

12.8.2 Adverse Events

The incidence of AE and severity of the AE will be summarized using descriptive statistics. Each patient will be counted only once by using the AEs with the highest severity within each category. Treatment-related AE summaries will include AEs related to medication and acupuncture.

Listings for SAEs, AEs will be presented. All information pertaining to AEs noted during the study will be listed by subject, detailing verbatim given by the investigator, date of onset, date of

resolution, severity, and relationship to treatment. The onset of AEs will also be shown relative (in number of days) to the first day of treatment. In addition, AE descriptions, and AE by patient number and treatment group will be presented.

13. MONITORING PROCEDURES

13.1 Procedures for Monitoring Participant Compliance

The investigator will be responsible for monitoring participant compliance (acupuncture sessions). If the principal investigator determines that the participant is not in compliance with the study protocol, the principal investigator must inform the Research Ethical Committee and they must review, discuss, and document the implications of the deviation.

13.2 Study Monitoring

To ensure compliance with GCP guidelines, the study monitor is responsible for ensuring that participants have signed the informed consent form and the study is conducted according to applicable SOPs, the protocol, and other written instructions and regulatory guidelines.

The main responsibilities of the study monitor are to visit the investigator before, during, and after the study to ensure adherence to the protocol, that all data are correctly and completely recorded and reported, and that informed consent is obtained and recorded for all participants before they participate in the study and when changes to the consent form are warranted, in accordance with Research Ethical Committee approvals.

The study monitor will be permitted to check and verify the various records (CRFs and other pertinent source data records, including source documentation) relating to the study to verify adherence to the protocol and to ensure the completeness, consistency, and accuracy of the data being recorded.

The investigator must agree to cooperate with the study monitor to resolve any problems, errors, or possible misunderstandings concerning the findings detected in the course of these monitoring visits and/or provided in follow-up written communication.

14.STUDY DISCONTINUATION

The investigators may choose to discontinue study treatment, if they think the continuation would be detrimental to the participants' well-being. Whenever possible, the participants should be re-challenged with study treatment if their conditions were considered appropriate by the investigator. Participants also have the right to discontinue study treatment at any time for any reason, without prejudice to further treatment. Such participants will always be asked about the primary reason for their decision to discontinue study treatment and the presence of AEs if any. If the discontinuation was due to AEs, refer to the **section 9**. It should be evaluated if the

discontinuation can be made temporarily, and permanent discontinuation should be the last choice.

It is essential to collect as much data as possible for all participants throughout the study, especially all potential endpoint events. Discontinuation of study treatment does not mean discontinuation of follow-up or termination of study participation. Participants who have discontinued performance of study treatment are expected to, and should be encouraged to the end of study.

15. STUDY ORGANISATION

15.1 Steering Committee

The steering committee will make ethical, scientific, and strategic decisions regarding the overall conduct of the trial, to ensure the study execution is of the highest quality. It will perform logistical coordination of different committees and will meet regularly to review the study progress. It will also review and approve the reporting and publications of the study.

Steering committee members: Bin Li, Lu Liu, Qiuyi Chen, Yazhuo Kong.

15.2 Executive Committee

The executive committee will help the steering committee maintain a high level of ethical, scientific, technical, and regulatory quality in all aspects of the trial. It will lead the successful implementation of the protocol according to the decisions made by the steering committee, and will monitor recruitment, compliance, and the adjudication process. It will meet more regularly to provide guidance for the day-to-day operations of the trial.

Executive committee members: Bin Li , Lu Liu, Bo Li, Jing Hu, Jing Wang.

15.3 Data Monitoring Committee

The data monitoring committee will meet periodically to monitor the progress of all aspects of the trial and ensure that the trial meets the highest standards of ethics and patient safety. The members may suggest trial amendments regarding the safety of patients or early trial termination, but the final decision rests with the steering committee. Members of the data monitoring committee will not participate in the trial.

Data monitoring committee members: Lin Zeng, Xun Li, Weijuan Gang, Shuiqing Zhang.

16. PUBLICATIONS

Principal investigator has ownership of all data and results collected during this trial. All decisions regarding the use of data and results for public presentations and publications must be approved by principal investigator.

All presentations and publications of the results will be based on clean, checked, and validated data in order to ensure the accuracy of the results. The results of this trial will be published irrespective of whether the results are regarded positive or negative.

17. FUNDING

This work was funded by Beijing Natural Science Foundation (7232270), China National Natural Science Foundation (82374575), Capital's Funds for Health Improvement and Research (CFH2024-2-2235), Out-standing Young Talents Program of Capital Medical University (B2207), and Beijing Hospital Management Center "peak" talent training plan team (DFL20241001).

18. REFERENCES

1. Wang F, Ma B, Ma Q, Liu X. Global, regional, and national burden of inguinal, femoral, and abdominal hernias: a systematic analysis of prevalence, incidence, deaths, and DALYs with projections to 2030. *Int J Surg*. 2024;110(4):1951-1967. doi:10.1097/JS9.0000000000001071
2. Diener H-C. CGRP as a new target in prevention and treatment of migraine. *Lancet Neurol*. 2014;13(11):1065-1067. doi:10.1016/S1474-4422(14)70228-5
3. Dodick DW. Triptan nonresponder studies: implications for clinical practice. *Headache*. 2005;45(2):156-162.
4. Pascual J. Recent advances in the pharmacological management of migraine. *F1000 Med Rep*. 2009;1doi:10.3410/M1-39
5. Linde K, Allais G, Brinkhaus B, Manheimer E, Vickers A, White AR. Acupuncture for migraine prophylaxis. *Cochrane Database Syst Rev*. 2009;(1):CD001218. doi:10.1002/14651858.CD001218.pub2
6. Fu C, Zhang Y, Ye Y, et al. Predicting response to tVNS in patients with migraine using functional MRI: A voxels-based machine learning analysis. *Front Neurosci*. 2022;16:937453. doi:10.3389/fnins.2022.937453
7. Horien C, Floris DL, Greene AS, et al. Functional Connectome-Based Predictive Modeling in Autism. *Biol Psychiatry*. 2022;92(8):626-642. doi:10.1016/j.biopsych.2022.04.008
8. Lehnertz K, Bröhl T, Wrede Rv. Epileptic-network-based prediction and control of seizures in humans. *Neurobiol Dis*. 2023;181:106098. doi:10.1016/j.nbd.2023.106098
9. Shen X, Finn ES, Scheinost D, et al. Using connectome-based predictive modeling to predict individual behavior from brain connectivity. *Nat Protoc*. 2017;12(3):506-518. doi:10.1038/nprot.2016.178
10. Maleki N, Becerra L, Brawn J, McEwen B, Burstein R, Borsook D. Common hippocampal structural and functional changes in migraine. *Brain Struct Funct*. 2013;218(4):903-912. doi:10.1007/s00429-012-0437-y
11. Maleki N, Becerra L, Brawn J, Bigal M, Burstein R, Borsook D. Concurrent functional and structural cortical alterations in migraine. *Cephalalgia*. 2012;32(8):607-620. doi:10.1177/0333102412445622
12. Schwedt TJ, Zuniga L, Chong CD. Low heat pain thresholds in migraineurs between attacks. *Cephalalgia*. 2015;35(7):593-599. doi:10.1177/0333102414550417
13. Zhang H, He S, Hu Y, Zheng H. Antagonism of cannabinoid receptor 1 attenuates the anti-inflammatory effects of electroacupuncture in a rodent model of migraine. *Acupunct Med*. 2016;34(6):463-470. doi:10.1136/acupmed-2016-011113

14. Wang L-P, Zhang X-Z, Guo J, et al. Efficacy of acupuncture for migraine prophylaxis: a single-blinded, double-dummy, randomized controlled trial. *Pain*. 2011;152(8):1864-1871. doi:10.1016/j.pain.2011.04.006
15. Zhao L, Chen J, Li Y, et al. The Long-term Effect of Acupuncture for Migraine Prophylaxis: A Randomized Clinical Trial. *JAMA Intern Med*. 2017;177(4):508-515. doi:10.1001/jamainternmed.2016.9378
16. Yang CP, Chang MH, Liu PE, et al. Acupuncture versus topiramate in chronic migraine prophylaxis: a randomized clinical trial. *Cephalalgia*. 2011;31(15):1510-1521. doi:10.1177/0333102411420585
17. Lipton RB, Bigal ME, Diamond M, Freitag F, Reed ML, Stewart WF. Migraine prevalence, disease burden, and the need for preventive therapy. *Neurology*. 2007;68(5):343-349.
18. Headache Classification Committee of the International Headache Society (IHS) The International Classification of Headache Disorders, 3rd edition. *Cephalalgia*. 2018;38(1)doi:10.1177/0333102417738202
19. Linde K, Allais G, Brinkhaus B, et al. Acupuncture for the prevention of episodic migraine. *Cochrane Database Syst Rev*. 2016;2016(6):CD001218. doi:10.1002/14651858.CD001218.pub3
20. Jiao Y, Wu Z, Hu J, et al. [Interpretation of Evidence-based Guidelines of Clinical Practice with Acupuncture and Moxibustion:Migraine of the version 2014]. *Zhongguo Zhen Jiu*. 2016;36(7):751-756. doi:10.13703/j.0255-2930.2016.07.022
21. Xu S, Yu L, Luo X, et al. Manual acupuncture versus sham acupuncture and usual care for prophylaxis of episodic migraine without aura: multicentre, randomised clinical trial. *BMJ*. 2020;368:m697. doi:10.1136/bmj.m697
22. Li Y, Zheng H, Witt CM, et al. Acupuncture for migraine prophylaxis: a randomized controlled trial. *CMAJ*. 2012;184(4):401-410. doi:10.1503/cmaj.110551

APPENDIX

Appendix A. Concomitant Treatments: Disallowed or Allowed with Restrictions

**Treatments Class**	**Details**
Other clinical study drug	Do not use within 3 months prior to the screening period.
Antimigraine agents	Acute treatment of migraine (prescription or over-the-counter medication recommended by a neurologist) is allowed provided the dose has been stable for at least 12 weeks prior to the screening period.<br>Do not use preventive migraine treatments within 3 months prior to the screening period and during the study. This includes daily use of:<br>– Beta blockers: metoprolol, propranolol, timolol, atenolol, nadolol, nebivolol, pindolol, or bisoprolol<br>– Anticonvulsants: carbamazepine, or gabapentin<br>– Tricyclic antidepressants: amitriptyline, nortriptyline, or protriptyline<br>– Calcium channel blockers: flunarizine or verapamil<br>– ACEI/ARB: candesartan or lisinopril<br>– Onabotulinumtoxin A<br>– Valproate<br>Do not use onabotulinumtoxin A for any medical or cosmetic reason requiring injections in the head, face, or neck during the 3 months before screening and during the study.
Interventions or devices for migraine	Do not use interventions or devices for migraine, during 3 months before screening. Interventions or devices for migraine are listed as: acupuncture, occipital stimulator, nerve blocks and transcranial magnetic stimulation.
Antihypertensives	See restrictions in use under anti-migraine agents. Other medication in the same class is allowed if prescribed for non-migraine indications.
Anticonvulsants	See restrictions in use under anti-migraine agents. Other medication in the same class is allowed if

	prescribed for non-migraine indications.
Anti-inflammatory agents	Allowed if prescribed for non-migraine indications, for example, low dose non-steroidal anti-inflammatory drugs (acetylsalicylic acid) for cardiovascular disease prevention.
Hormones	Hormonal therapy (for example, contraceptives, hormone replacement therapy) is allowed provided the dose has been stable for at least 3 months prior to the screening period.
Sedatives/hypnotics	Limited use of the following is allowed provided a stable regimen (<4 days per month) has been maintained for at least 3 months prior to the screening period. These agents may be prescribed when considered medically indicated by the neurologist during the study (including the screening period) providing its use does not exceed 4 days per month:<br>– barbiturates<br>– prescription opiates

Appendix B. Headache Diary

<u>**Study Period**</u>	<u>**Baseline period**</u>	<u>**Treatment period**</u>
（Please draw √ inside the present period brackets）	(-4-0 Week)<br>( )	(1-4 Week)<br>( )

<u>Subject Diary Card - Headache diary</u>

Random Number：____________________________

Time of This Visit：___________________(yy/mm/dd)

[Filling Explanation]

Your headache diary is used to record all the symptoms associated with your headache. Please try your best to answer these questions on the day of headache or the day after headache. You need to record all symptoms you observe for 4 weeks. Please try your best to ensure accuracy and completeness. Your headache diary can help your doctor develop the right treatment strategy. Your symptoms may not be obvious during this time, but once you document each of your symptoms, your pattern of symptoms and the progression of your disease over time will give a strong indication of your condition.

Your charge doctor:________________________________

Tel of your charge doctor: ___________________________

Explanation 1: Please record a headache diary **at 19:00** every day and recall your headache from 19:00 yesterday to now **without blank**. If you have a headache, please truthfully record it in the diary, and be sure to record all the details. And remember to **bring back** this headache diary on each subsequent visit. The following are the problems you may encounter when filling. If you have any questions during the filling process, please contact your doctor in charge.

• If your headache has not ended at the time of filling, please fill in "**Still continuing**" in the “End time” column

• If the headache doesn’t happen, please fill in with “**No Headache**” in the “Start time” and “End time” column.

• If you do not have headache but **have aura**, please truthfully record the aura and the medication intake.

• If your headache **recurred or disappeared within 1 day**, please fill in the start time of the earliest headache in the “Start time” column and the end time of the headache closest to the time you record today in the “End time” column.

• If the duration of a headache is **longer than 24:00 of the previous day**, when recording the headache diary on the second day, please fill in the start time of the earliest headache of the previous day in the “Start time” column and the end time of the headache closest to the time you record today in the “End time” column.

• If you **did not fill in the diary for some reason in the previous day**, please fill in the headache condition of the previous day truthfully on the second day. If you have missed records from the previous 2 or more days, you can only fill in the latest 1 day headache diary.

Explanation 2: Aura:

• **Optic aura**: visual disturbance as spots, stars, flashes, zigzag lines, heat waves or complete or partial loss of vision around the time of your headache.

• **Sensory aura:** feeling disturbance as numbness or tingling in any part of your body or face

around the time of your headache.

• The discomfort in the head and neck is excluded from aura for the reason that it indicates the beginning of headache.

Explanation 3：Intensity scale:

• 0 = No pain • 1(Mild) = Not affect daily activities • 2 (moderate) = Affect daily activities

• 3 (Severe) = Unable to perform daily activities.

Explanation 4：VAS score:

Suppose 0 is no pain and 10 is the worst pain you can imagine in the world.

VAS score: please read the severity of your headache in the position:

No pain |____|____|____|____|____|____|____|____|____|____| the worst pain

0 1 2 3 4 5 6 7 8 9 10

Explanation 5： If you still feel headache when you go to sleep the day before, and the headache disappears when you wake up the next morning, the end time of headache is counted as the time when you wake up the next day.

Explanation 6： Please fill in the time in **24 hours format** : year, month, day, hour.

Explanation 7： The name of the drug should be filled in the record of medication intake, ? tablet/time, ? time/day, a total of ? day.

Explanation 8：Triggers of Headache (Headache related climate change, environment change, physical discomfort, stress, tiredness, special diet, menstruation, etc.)

Explanation 9：Female subjects please fill in your menstrual start and end time:_____to_____

Headache diary table								
Symptom \ Date								
Headache duration	Start time							
	End time							
Intensity scale（0-3）	0-3* (Explanation 2)							
VAS	0-10* (Explanation 3)							
Was it worse on one side of the head than on the other, and/or limited to one side of the head?		☐Yes<br>--☐Left<br>--☐Right<br>☐No	☐Yes<br>--☐Left<br>--☐Right<br>☐No	☐Yes<br>--☐Left<br>--☐Right<br>☐No	☐Yes<br>--☐Left<br>--☐Right<br>☐No	☐Yes<br>--☐Left<br>--☐Right<br>☐No	☐Yes<br>--☐Left<br>--☐Right<br>☐No	☐Yes<br>--☐Left<br>--☐Right<br>☐No
Headache site	(Left, right, temporal, parietal, occipitalia, forehead)	☐Left<br>☐Right<br>☐Temporal<br>☐Parietal<br>☐Occipitalia	☐Left<br>☐Right<br>☐Temporal<br>☐Parietal<br>☐Occipitalia	☐Left<br>☐Right<br>☐Temporal<br>☐Parietal<br>☐Occipitalia	☐Left<br>☐Right<br>☐Temporal<br>☐Parietal<br>☐Occipitalia	☐Left<br>☐Right<br>☐Temporal<br>☐Parietal<br>☐Occipitalia	☐Left<br>☐Right<br>☐Temporal<br>☐Parietal<br>☐Occipitalia	☐Left<br>☐Right<br>☐Temporal<br>☐Parietal<br>☐Occipitalia

		☐Forehead	☐Forehead	☐Forehead	☐Forehead	☐Forehead	☐Forehead	☐Forehead
**Was it pulsating?**		☐Yes<br>☐No	☐Yes<br>☐No	☐Yes<br>☐No	☐Yes<br>☐No	☐Yes<br>☐No	☐Yes<br>☐No	☐Yes<br>☐No
**Aggravation after daily activities(Walk or climb stairs, etc.)**		☐Yes<br>☐No	☐Yes<br>☐No	☐Yes<br>☐No	☐Yes<br>☐No	☐Yes<br>☐No	☐Yes<br>☐No	☐Yes<br>☐No
**Accompanying symptom**	**Nausea**	☐Yes<br>☐No	☐Yes<br>☐No	☐Yes<br>☐No	☐Yes<br>☐No	☐Yes<br>☐No	☐Yes<br>☐No	☐Yes<br>☐No
	**Vomit**	☐Yes<br>☐No	☐Yes<br>☐No	☐Yes<br>☐No	☐Yes<br>☐No	☐Yes<br>☐No	☐Yes<br>☐No	☐Yes<br>☐No
	**Light intolerance**	☐Yes<br>☐No	☐Yes<br>☐No	☐Yes<br>☐No	☐Yes<br>☐No	☐Yes<br>☐No	☐Yes<br>☐No	☐Yes<br>☐No
	**Noise intolerance**	☐Yes<br>☐No	☐Yes<br>☐No	☐Yes<br>☐No	☐Yes<br>☐No	☐Yes<br>☐No	☐Yes<br>☐No	☐Yes<br>☐No
	**Other symptoms**	(______)	(______)	(______)	(______)	(______)	(______)	(______)
**Aura* (Explanation 2)**<br>(Please write the specific aura in the parentheses)		☐Without<br>☐Optic<br>(______)<br>☐Sensory<br>(______)<br>☐Other	☐Without<br>☐Optic<br>(______)<br>☐Sensory<br>(______)<br>☐Other	☐Without<br>☐Optic<br>(______)<br>☐Sensory<br>(______)<br>☐Other	☐Without<br>☐Optic<br>(______)<br>☐Sensory<br>(______)<br>☐Other	☐Without<br>☐Optic<br>(______)<br>☐Sensory<br>(______)<br>☐Other	☐Without<br>☐Optic<br>(______)<br>☐Sensory<br>(______)<br>☐Other	☐Without<br>☐Optic<br>(______)<br>☐Sensory<br>(______)<br>☐Other

		(______)	(______)	(______)	(______)	(______)	(______)	(______)
**Whether you want to lie down during headache? If yes, please fill in the affected time (hours).**		☐Yes<br>( )h<br>☐No	☐Yes<br>( )h<br>☐No	☐Yes<br>( )h<br>☐No	☐Yes<br>( )h<br>☐No	☐Yes<br>( )h<br>☐No	☐Yes<br>( )h<br>☐No	☐Yes<br>( )h<br>☐No
**Whether your daily study or work are impaired during headache?**<br>**If yes, please fill in the affected time (hours) and the percent impaired during headache.**		☐Yes<br>( )h<br>( )%<br>☐No	☐Yes<br>( )h<br>( )%<br>☐No	☐Yes<br>( )h<br>( )%<br>☐No	☐Yes<br>( )h<br>( )%<br>☐No	☐Yes<br>( )h<br>( )%<br>☐No	☐Yes<br>( )h<br>( )%<br>☐No	☐Yes<br>( )h<br>( )%<br>☐No
**Whether your daily activities are impaired during headache?**<br>**If yes, please fill in the affected time (hours) and the percent impaired during headache.**		☐Yes<br>( )h<br>( )%<br>☐No	☐Yes<br>( )h<br>( )%<br>☐No	☐Yes<br>( )h<br>( )%<br>☐No	☐Yes<br>( )h<br>( )%<br>☐No	☐Yes<br>( )h<br>( )%<br>☐No	☐Yes<br>( )h<br>( )%<br>☐No	☐Yes<br>( )h<br>( )%<br>☐No
**Triggers of headache***<br>**(Explanation 8)**								
**Analgesic drugs intake**	**Name**	☐Triptan<br>(______)<br>☐Ergot<br>(______)<br>☐Other	☐Triptan<br>(______)<br>☐Ergot<br>(______)<br>☐Other	☐Triptan<br>(______)<br>☐Ergot<br>(______)<br>☐Other	☐Triptan<br>(______)<br>☐Ergot<br>(______)<br>☐Other	☐Triptan<br>(______)<br>☐Ergot<br>(______)<br>☐Other	☐Triptan<br>(______)<br>☐Ergot<br>(______)<br>☐Other	☐Triptan<br>(______)<br>☐Ergot<br>(______)<br>☐Other

		(_______)	(_______)	(_______)	(_______)	(_______)	(_______)	(_______)
	**Dose and frequency**							
	**Headache 0.5 hour after taking analgesic**	☐disappear<br>☐remission<br>☐no change	☐disappear<br>☐remission<br>☐no change	☐disappear<br>☐remission<br>☐no change	☐disappear<br>☐remission<br>☐no change	☐disappear<br>☐remission<br>☐no change	☐disappear<br>☐remission<br>☐no change	☐disappear<br>☐remission<br>☐no change
	**Headache 2 hours after taking analgesic**	☐disappear<br>☐remission<br>☐no change	☐disappear<br>☐remission<br>☐no change	☐disappear<br>☐remission<br>☐no change	☐disappear<br>☐remission<br>☐no change	☐disappear<br>☐remission<br>☐no change	☐disappear<br>☐remission<br>☐no change	☐disappear<br>☐remission<br>☐no change
	**Recovery of daily activity ability 2 hours after taking analgesic**	☐Unrecovered<br>☐Basically recovered	☐Unrecovered<br>☐Basically recovered	☐Unrecovered<br>☐Basically recovered	☐Unrecovered<br>☐Basically recovered	☐Unrecovered<br>☐Basically recovered	☐Unrecovered<br>☐Basically recovered	☐Unrecovered<br>☐Basically recovered
**Other drug, treatment or methods were used**								
**Adverse event**								

Appendix C. Headache Impact Test-6 (HIT-6)

HIT is a tool used to measure the impact headaches have on your ability to function on the job, at school, at home and in social situations. Your score shows you the effect that headaches have on normal daily life and your ability to function. HIT was developed by an international team of headache experts from neurology and primary care medicine in collaboration with the psychometricians who developed the SF-36 health assessment tool. This questionnaire was designed to help you describe and communicate the way you feel and what you cannot do because of headaches.

To complete, please circle one answer for each question.

When you have headaches, how often is the pain severe?

never rarely sometimes very often always

How often do headaches limit your ability to do usual daily activities including household work, work, school, or social activities?

never rarely sometimes very often always

When you have a headache, how often do you wish you could lie down?

never rarely sometimes very often always

In the past 4 weeks, how often have you felt too tired to do work or daily activities because of your headaches?

never rarely sometimes very often always

In the past 4 weeks, how often have you felt fed up or irritated because of your headaches?

never rarely sometimes very often always

In the past 4 weeks, how often did headaches limit your ability to concentrate on work or daily activities?

never rarely sometimes very often always

☐ +	☐ +	☐ +	☐ +	☐
COLUMN 1	COLUMN 2	COLUMN 3	COLUMN 4	COLUMN 5
6 points each	8 points each	10 points each	11 points each	13 points each

To score, add points for answers in each column.

If your HIT-6 is 50 or higher:

You should share your results with your doctor. Headaches that stop you from enjoying the important things in life, like family, work, school or social activities could be migraine.

☐

TOTAL

SCORE

Appendix D. Migraine-Specific Quality Of Life Questionnaire (MSQ) (VERSION 2.1)

While answering the following questions, please think about all migraine attacks you may have had in the past 4 weeks.

1. In the past 4 weeks, how often have migraines **interfered** with how well you dealt with family, friends and others who are close to you? (Select only **one** response.)

1☐ None of the time

2☐ A little bit of the time

3☐ Some of the time

4☐ A good bit of the time

5☐ Most of the time

6☐ All of the time

2. In the past 4 weeks, how often have migraines **interfered** with your leisure time activities, such as reading or exercising? (Select only **one** response.)

1☐ None of the time

2☐ A little bit of the time

3☐ Some of the time

4☐ A good bit of the time

5☐ Most of the time

6☐ All of the time

3. In the past 4 weeks, how often have you had **difficulty** in performing work or daily activities because of migraine symptoms? (Select only **one** response.)

1☐ None of the time

2☐ A little bit of the time

3☐ Some of the time

4☐ A good bit of the time

5☐ Most of the time

6☐ All of the time

4. In the past 4 weeks, how often did migraines **keep you** from getting as much done at work or at home? (Select only **one** response.)

1☐ None of the time

2☐ A little bit of the time

3☐ Some of the time

4☐ A good bit of the time

5☐ Most of the time

6☐ All of the time

5. In the past 4 weeks, how often did migraines **limit** your ability to concentrate on work or daily activities? (Select only **one** response.)

1☐ None of the time

2☐ A little bit of the time

3☐ Some of the time

4☐ A good bit of the time

5☐ Most of the time

6☐ All of the time

6. In the past 4 weeks, how often have migraines **left you too tired** to do work or daily activities? (Select only **one** response.)

1☐ None of the time

2☐ A little bit of the time

3☐ Some of the time

4☐ A good bit of the time

5☐ Most of the time

6☐ All of the time

7. In the past 4 weeks, how often have migraines **limited** the number of days you

have felt energetic? (Select only **one** response.)

1☐ None of the time

2☐ A little bit of the time

3☐ Some of the time

4☐ A good bit of the time

5☐ Most of the time

6☐ All of the time

8. In the past 4 weeks, how often have you had to **cancel** work or daily activities because you had a migraine? (Select only **one** response.)

1☐ None of the time

2☐ A little bit of the time

3☐ Some of the time

4☐ A good bit of the time

5☐ Most of the time

6☐ All of the time

9. In the past 4 weeks, how often did you **need help** in handling routine tasks such as every day household chores, doing necessary business, shopping, or caring for others, when you had a migraine? (Select only **one** response.)

1☐ None of the time

2☐ A little bit of the time

3☐ Some of the time

4☐ A good bit of the time

5☐ Most of the time

6☐ All of the time

10. In the past 4 weeks, how often did you have to **stop** work or daily activities to deal with migraine symptoms? (Select only **one** response.)

1☐ None of the time

2☐ A little bit of the time

3☐ Some of the time

4☐ A good bit of the time

5☐ Most of the time

6☐ All of the time

11. In the past 4 weeks, how often were you **not able to go** to social activities such as parties, dinner with friends, because you had a migraine? (Select only **one** response.)

1☐ None of the time

2☐ A little bit of the time

3☐ Some of the time

4☐ A good bit of the time

5☐ Most of the time

6☐ All of the time

12. In the past 4 weeks, how often have you **felt** fed up or frustrated because of your migraines? (Select only **one** response.)

1☐ None of the time

2☐ A little bit of the time

3☐ Some of the time

4☐ A good bit of the time

5☐ Most of the time

6☐ All of the time

13. In the past 4 weeks, how often have you **felt** like you were a burden on others because of your migraines? (Select only **one** response.)

1☐ None of the time

2☐ A little bit of the time

3☐ Some of the time

4☐ A good bit of the time

5☐ Most of the time

6☐ All of the time

14. In the past 4 weeks, how often have you been **afraid** of letting others down because of your migraines? (Select only **one** response.)

1☐ None of the time

2☐ A little bit of the time

3☐ Some of the time

4☐ A good bit of the time

5☐ Most of the time

6☐ All of the time

Appendix E. Patient Global Impression of Change Scale (PGIC)

Since beginning treatment at this clinic, how would you describe the change (if any) in ACTIVITY LIMITATIONS, SYMPTOMS, EMOTIONS, and OVERALL QUALITY OF LIFE, related to your painful condition? Please circle the number below that matches your degree of change since beginning care at this clinic for the above stated chief complaint.

No change	Almost the same	A little better	Somewhat better	Moderately better	Better	A great deal better
1	2	3	4	5	6	7

Explanation:

1 = No change (or condition has got worse)

2 = Almost the same, hardly any change at all

3 = A little better, but no noticeable change

4 = Somewhat better, but the change has not made any real difference

5 = Moderately better, and a slight but noticeable change

6 = Better, and a definite improvement that has made a real and worthwhile difference

7 = A great deal better, and a considerable improvement that has made all the difference

Do not write in this box - FOR OFFICE USE ONLY

NOTE TO HEALTH CARE PROVIDER

A significant, favorable change is a score of 5- 7

No significant change is a 1-4 response.

Note, this is a dichotomous scale (5-7 = yes; 1-4 = no).

A 2-point change is significant from their last reported score.

Reference: Hurst H, Bolton J. Assessing the clinical significance of change scores recorded on subjective outcome measures. Journal of Manipulative Physiological Therapeutics (IMPT) 2004;27:26-35.

Appendix F. Acupuncture Expectancy Scale (AES)

Every individual may have different expectation for the effects of acupuncture. If we use the following sentences to describe your expectation of acupuncture's effect on your illness/symptom after the entire course of acupuncture therapy, how much do you agree? For each statement, please choose the closest answer.

1. My illness will improve a lot.

1 (Not at All Agree) 2 (A Little Agree) 3 (Moderately Agree)

4 (Mostly Agree) 5 (Completely Agree)

2. I will be able to cope with my illness better.

1 (Not at All Agree) 2 (A Little Agree) 3 (Moderately Agree)

4 (Mostly Agree) 5 (Completely Agree)

3. The symptoms of my illness will disappear.

1 (Not at All Agree) 2 (A Little Agree) 3 (Moderately Agree)

4 (Mostly Agree) 5 (Completely Agree)

4. My energy level will increase

1 (Not at All Agree) 2 (A Little Agree) 3 (Moderately Agree)

4 (Mostly Agree) 5 (Completely Agree)

Appendix G. Blinding Questionnaire

Do you think which kind of treatment group you have participated in the past weeks?

☐ Real acupuncture group

☐ Sham acupuncture group

☐ Did not know
