# Supplementary material for: Acupuncture for Migraine Without Aura and Connection-Based Efficacy Prediction: A Randomized Clinical Trial
Source: JAMA Netw Open. 2026 Jan 27;9(1):e2555454. doi: 10.1001/jamanetworkopen.2025.55454 (PMC12848631; doi:10.1001/jamanetworkopen.2025.55454)
Supplement: Supplement 3. — eMethods 1. Patient Recruitment and Study Sample Selection eMethods 2. Scales for Outcome Measures eMethods 3. Neuroimaging Data Acquisition eMethods 4. Image Preprocessing eMethods 5. Shen Atlas Registration eMethods 6. Network Anatomy eAppendix. Negative Results eFigure 1. The Real Acupoints Location eFigure 2. The Sham Acupoints Location eFigure 3. The Flowchart of Connectome-based Predictive Modeling eTable 1. The Real Acupoints Location eTable 2. The Manipulations of the Real Acupoints eTable 3. The Sham Acupoints Location eTable 4. Baseline Characteristics in the Intention-to-Treat Population eTable 5. Baseline Characteristics in the Per-Protocol Population eTable 6. Primary and Secondary Clinical Efficacy Outcomes in the Per-Protocol Population During the Treatment Period eTable 7. Participants’ Satisfaction at Week 4 eTable 8. Acupuncture Expectancy Scale Score at Baseline eTable 9. Compliance Data at Week 4 eTable 10. Participant-Blinding Assessment Results eTable 11. Adverse Events Related to Treatment eReferences [file jamanetwopen-e2555454-s003.pdf]

Supplemental Online Content

Zhang X, Chen Q, Liu Y, et al. Acupuncture for migraine without aura and connection-based efficacy prediction: a randomized clinical trial. *JAMA Netw Open*. 2026;9(1):e2555454. doi:10.1001/jamanetworkopen.2025.55454

eMethods 1. Patient Recruitment and Study Sample Selection

eMethods 2. Scales for Outcome Measures

eMethods 3. Neuroimaging Data Acquisition

eMethods 4. Image Preprocessing

eMethods 5. Shen Atlas Registration

eMethods 6. Network Anatomy

eAppendix. Negative Results

eFigure 1. The Real Acupoints Location

eFigure 2. The Sham Acupoints Location

eFigure 3. The Flowchart of Connectome-based Predictive Modeling

eTable 1. The Real Acupoints Location

eTable 2. The Manipulations of the Real Acupoints

eTable 3. The Sham Acupoints Location

eTable 4. Baseline Characteristics in the Intention-to-Treat Population

eTable 5. Baseline Characteristics in the Per-Protocol Population

eTable 6. Primary and Secondary Clinical Efficacy Outcomes in the Per-Protocol Population During the Treatment Period

eTable 7. Participants' Satisfaction at Week 4

eTable 8. Acupuncture Expectancy Scale Score at Baseline

eTable 9. Compliance Data at Week 4

eTable 10. Participant-Blinding Assessment Results

eTable 11. Adverse Events Related to Treatment

eReferences

This supplemental material has been provided by the authors to give readers additional information about their work.

© 2026 Zhang X et al. *JAMA Network Open*

eMethods 1. Patient Recruitment and Study Sample Selection

Patients with migraine without aura (MWoA) were recruited from the outpatient acupuncture clinics at Beijing TCM Hospital between July 2020 and June 2023, as part of a 4-week clinical trial evaluating acupuncture for the treatment of MWoA. Eligibility was determined by a neurologist, who assessed potential participants based on pre-defined inclusion and exclusion criteria.

The eligibility criteria were as follows: (i) diagnosis of MWoA based on the International Classification of Headache Disorders, 3rd edition (ICHD-3)[1]; (ii) age between 18 and 65 years; (iii) having at least two migraine attacks in the last 4 weeks; (iv) a history of migraine for at least one year; (v) ability to complete a headache diary; (vi) provided written informed consent; (vii) right-handedness.

The exclusion criteria were as follows: (i) diagnoses of new daily persistent headache, tension-type headache, trigeminal autonomic cephalalgia, or painful cranial neuropathy as defined by the ICHD-3; (ii) use of migraine preventive medications or prior experience with acupuncture or migraine devices within the past 3 months, or a history of medication overuse headache; (iii) presence of clinically significant diseases, including autoimmune disorders, cardiovascular, hepatic, respiratory, hematological, endocrine, psychiatric or neurological disease; (iv) pregnancy, lactation, or inadequate contraception; (v) contraindications to magnetic resonance imaging (MRI) (e.g., claustrophobia, cardiac pacemaker, or other metallic implants); (vi) evidence of drug or alcohol abuse or dependence within the past 12 months; (vii) use of opioid analgesics.

© 2026 Zhang X et al. *JAMA Network Open*

eMethods 2. Scales for Outcome Measures

Visual Analog Scale (VAS) is a rapid and simple tool for assessing pain intensity, applicable to various types of pain assessments, including acute and chronic pain[2]. It uses a simple 0 to 10-point scoring system to measure the severity of pain, with 0 indicating no pain and 10 representing unbearable severe pain. Patients are asked to mark a point on the scale according to their pain level.

6-item Headache Impact Test (HIT-6) is a six-item questionnaire designed to evaluate the impact of headaches on daily life and functioning in areas such as work, school, home, and social activities over the past 4 weeks[3,4]. Each of the six questions is scored using five response categories (never, rarely, sometimes, very often, or always) with corresponding points of 6, 8, 10, 11, or 13. The total HIT-6 score ranges from 36 to 78 and is interpreted as little or no effect (≤49), some effect (50–55), substantial effect (56–59), and severe effect (60–78), where higher scores indicate a greater impact of headaches, and lower scores indicate improvement.

The Migraine-Specific Quality of Life (MSQoL)[5,6], a 14-item patient-reported outcome instrument, evaluates the impact of migraine on three key domains: role restrictive (RR), role preventive (RP), and emotional functioning (EF). The raw domain scores are summed from the 14 items and then converted to a 0 to 100-point scale, where higher scores reflect a superior quality of life. This questionnaire has been translated into Chinese and validated for use.

© 2026 Zhang X et al. *JAMA Network Open*

eMethods 3. Neuroimaging Data Acquisition

MRI scans were conducted before and after 4 weeks of acupuncture treatment using a 3.0 T MAGNETOM Skyra Siemens with a 32-channel phase-array head coil at the MRI Center of Dong Zhimen Hospital Beijing University of Chinese Medicine. All participants underwent MRI scanning in the migraine's interictal phase (i.e., a minimum of 72 hours after a migraine attack). Participants were instructed to remain awake, relax, keep their eyes open, blink normally when staring at centrally positioned fixation cross (+), and to avoid thinking of anything in particular during the scan.

Functional images were obtained using the gradient-echo echo-planar pulse sequence (time repetition [TR]/time echo [TE] = 2420/30 ms; flip angle (FA) = 90°, voxel size = $3.1\times3.1\times4.0$ mm^3). A single-shot spin echo-based planar imaging sequence was employed to acquire the DTI (TR/TE = 12,700/91 ms, slice thickness = 2 mm, FA = 90◦, number of slices = 65, field of view (FOV) = 192×192 mm^2, matrix size = 96×96). 64 diffusion gradient orientations were used in Diffusion Tensor Imaging (DTI) scans (b = 1,000 s/mm^2), with the b = 0 repeated 2 times. High-resolution brain T1-weighted brain anatomical images were collected using a volumetric three-dimensional magnetization prepared by a rapid acquisition gradient-echo (MPRAGE) sequence with the following parameters: TR/TE = 2000/3.51 ms, voxel size: $1.3\times1.3\times1.3$ mm^3, FOV = 256×256 mm^2, FA = 8°.

© 2026 Zhang X et al. *JAMA Network Open*

eMethods 4. Image preprocessing

Functional imaging data preprocessing was performed using SPM12 (http://www.fil.ion.ucl.ac.uk/spm) and DPABI V5.1_201201 (5.1, advanced edition, https://rfmri.org/DPABI). First, raw data in the EPI DICOM format were converted to NIFTI format. The preprocessing steps included: (1) removal of the first 10 time points, (2) slice-timing correction, (3) head motion correction, (4) segmentation into gray matter, white matter, and cerebrospinal fluid (CSF), (5) spatial smoothing with a Gaussian kernel of 6 mm full-width at half-maximum, (6) linear detrending, (7) nuisance covariate regression and (8) band-pass temporal filtering (0.01 – 0.08 Hz).

© 2026 Zhang X et al. *JAMA Network Open*

eMethods 5. Shen atlas registration

The 268-node atlas was shifted from the MNI template space to the single-subject space by a sequence of integrated linear and non-linear registrations of the functional images, MPRAGE scans, and the MNI brain[7]. Each transformation pair was calculated independently and then synthesized into a single transform, which was inverted to align the functional atlas with the individual. This method, involving a solitary transformation, effectively reduces interpolation errors.

© 2026 Zhang X et al. *JAMA Network Open*

eMethods 6. Network anatomy

Networks identified and summarized using connectome-based predictive modeling (CPM) are typically complex, encompassing a multitude of nodes with diverse distributions. The connectivity values among these nodes range from high to low. Consequently, in research pertaining to whole-brain connectomes, we partition and summarize these networks by assigning nodes to either macroscale brain regions (such as the prefrontal cortex and cerebellum) or established functional networks (such as the Default Mode Network and Motor network)[8,9], through the overlap of the nodes identified by CPM with these regions and networks.

eAppendix. Negative results

Neither positive nor negative network strengths showed significant correlation with changes in the monthly migraine days (MMDs) (r_{pos} = 0.03; r_{neg} = 0.03; permutation P_{pos} = .31, P_{neg} = .33), monthly headache days (MHDs) (r_{pos} = 0.16; r_{neg} = -0.13; permutation P_{pos} = .10, P_{neg}= .67), acute medication use days (r_{pos} = 0.02; r_{neg} = -0.33; permutation P_{pos} = .35, P_{neg} = .92), MSQoL RR (r_{pos} = -0.02; r_{neg} = -0.20; permutation P_{pos} = .43, P_{neg} = .79), MSQoL RP (r_{pos} = 0.08; r_{neg} = 0.03; permutation P_{pos} = .22, P_{neg}= .33), or MSQoL EF (r_{pos} = 0.14; r_{neg} = -0.29; permutation P_{pos} = .12, P_{neg} = .90).

© 2026 Zhang X et al. *JAMA Network Open*

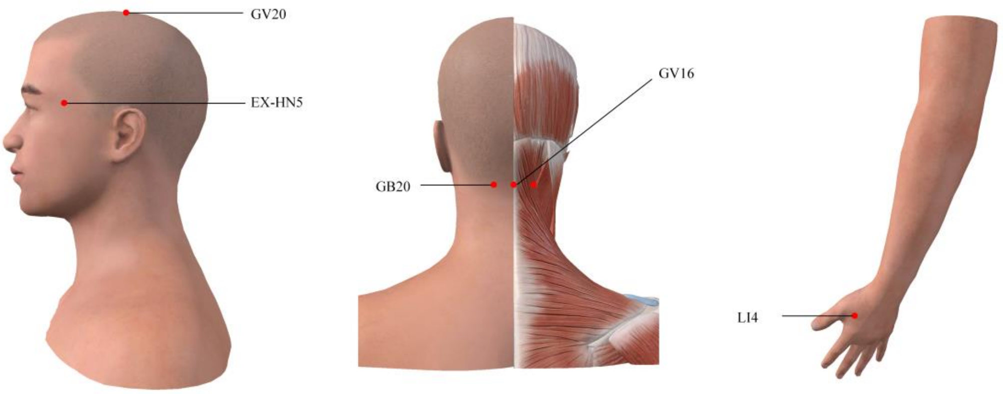

eFigures 1. The real acupoints location

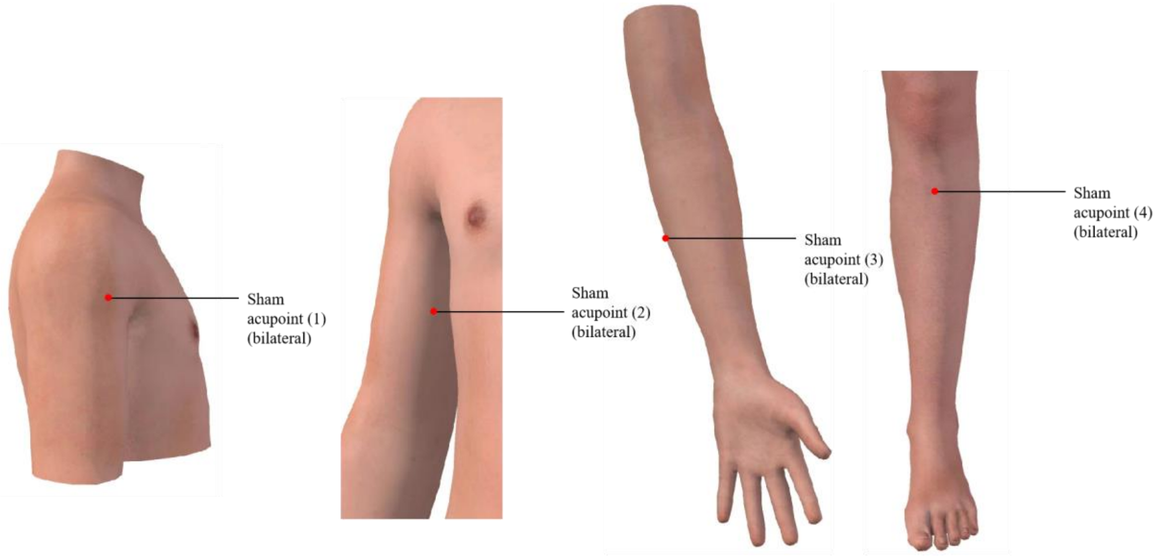

eFigure 2. The sham acupoints location

© 2026 Zhang X et al. *JAMA Network Open*

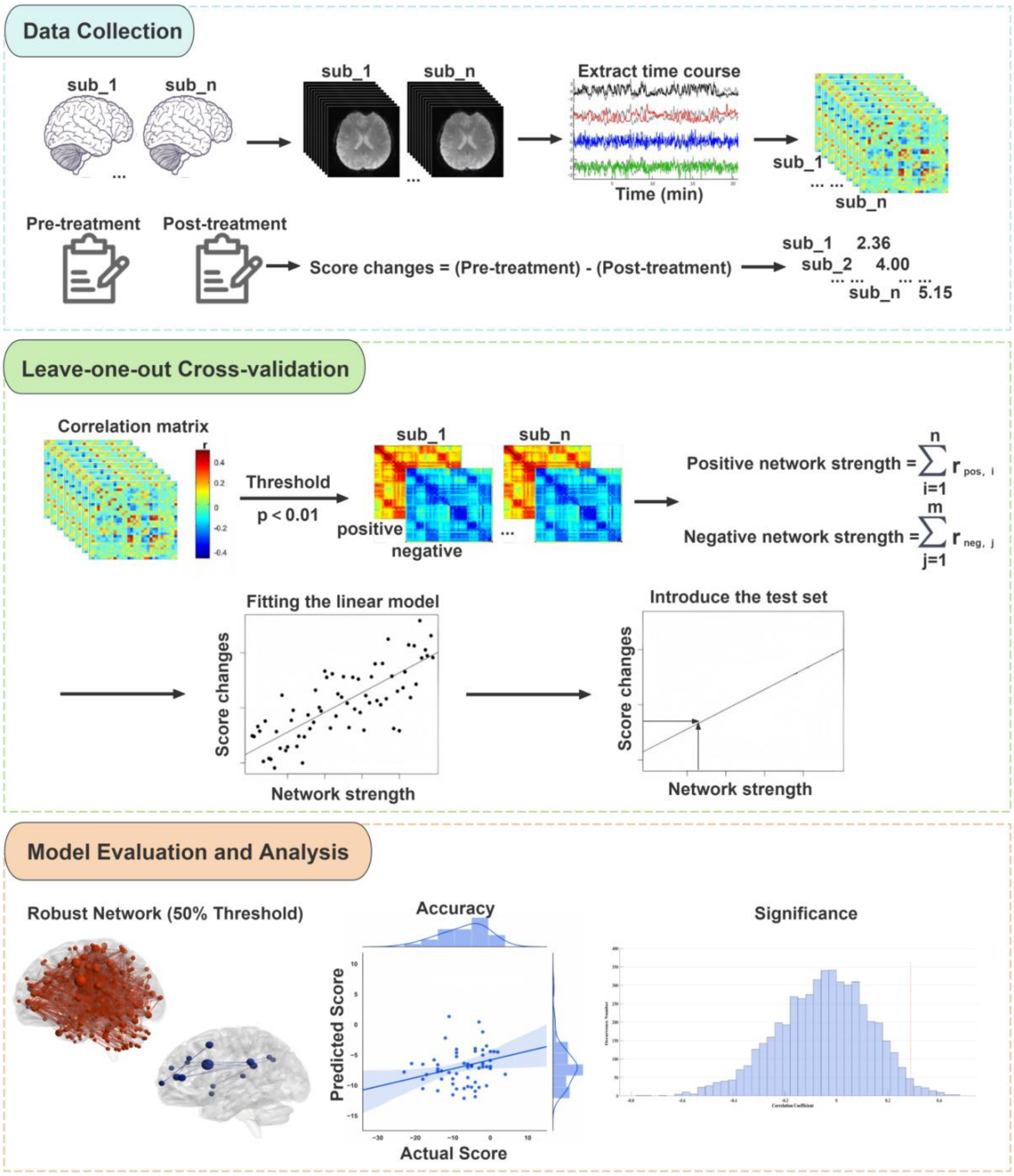

eFigures 3. The flowchart of Connectome-based predictive modeling

© 2026 Zhang X et al. *JAMA Network Open*

eTable 1. The real acupoints location

Acupoint	Meridian	Location
Bai Hui	GV20	The point is located at the top of the head, at the midpoint of the line connecting the apexes of both ears, corresponding to the vertex of the sagittal suture.
Feng Fu	GV16	Feng Fu is situated at the nape of the neck, in the depression immediately below the hairline, directly above the spinous process of the second cervical vertebra (C2).
Feng Chi	GB20	This point is found at the back of the neck, in the groove between the styloid process of the temporal bone and the upper edge of the trapezius muscle, approximately at the level of the C2-C3 interspinous space.
Tai Yang	EX-HN5	Tai Yang is located in the temporal region, about one inch posterior to the midpoint of the eyebrows, in the depression lateral to the orbit of the eye.
He Gu	LI4	He Gu is situated on the dorsum of the hand, between the first and second metacarpal bones, in the depression when the thumb and index finger are adducted. The point is typically marked at the highest point of the muscle belly when the hand is in a relaxed position.

© 2026 Zhang X et al. *JAMA Network Open*

eTable 2. The manipulations of the real acupoints.

Kinds of acupoint	Acupoints	Manipulation	Needle	Deqi
Du Meridian	Baihui (GV20), Fengfu (GV16)	GV20: obliquely inserted at an angle of 30-45 degree to a depth of 10-15mm. GV16: Fengfu: inserted obliquely at an angle of 15-30 degrees to a depth of 10-15mm.	Length: 25 Diameter: 0.30	Twirling, lifting, and thrusting (needle manipulati on) will be performed to produce deqi.
Shaoyang Meridian	Fengchi (GB20), bilateral Taiyang (EX-HN5), bilateral	Inserted vertically for about 10-15mm.	Length: 25 Diameter: 0.25	
Yangming Meridian	Hegu (LI4), bilateral	Inserted vertically for about 10-15mm.	Length: 40 Diameter: 0.30	

© 2026 Zhang X et al. *JAMA Network Open*

eTable 3. The sham acupoints location

Acupoint	Location
Sham acupoint (1)	On the medial aspect of the arm, at the anterior margin of the deltoid muscle insertion, where the deltoid meets the biceps muscle.
Sham acupoint (2)	Mid-way between the tip of the elbow and the axilla.
Sham acupoint (3)	On the ulnar side of the arm, at the mid-point between the medial epicondyle of the humerus and the ulnar side of the wrist.
Sham acupoint (4)	Along the edge of the tibia, approximately 1–2 cm lateral to and on the same horizontal level as Zusanli [ST36][a].

[a]Zusanli [ST36] is located 3 cun directly below Dubi and 1 finger-width lateral to the anterior edge of the tibia. As for the location of Dubi, when the knee is bent, it is at the knee, beneath the patella, in the depression beside the patellar ligament.

© 2026 Zhang X et al. *JAMA Network Open*

eTable 4. Baseline characteristics in the intention-to-treat population

Characteristics	Real acupuncture group (n = 60)	Sham acupuncture group (n = 60)
**Age, mean (SD), years**	36.9 (10.2)	37.0 (9.8)
**Sex, No. (%)**		
Female	48 (80.0)	47 (78.3)
Male	12 (20.0)	13 (21.7)
**Current employment status, No. (%)**		
Employed	50 (83.3)	47 (78.3)
Student	8 (13.3)	10 (16.7)
Unemployed, retired	2 (3.3)	3 (5.0)
**Education, No. (%)**		
≤High school	5 (8.3)	4 (6.7)
College	44 (73.3)	46 (76.7)
Graduate degree	11 (18.3)	10 (16.7)
**Previous use of preventive medications, No. (%)**		
Naïve	36 (60.0)	35 (58.3)
Previous use[a]	24 (40.0)	25 (41.7)
Previous preventive medication failure[b]	21 (35.0)	20 (33.3)
**Disease duration, mean (SE), years**	15.9 (1.2)	16.1 (1.1)
**MMDs at baseline, mean (SE)**	6.5 (0.3)	6.6 (0.3)
**MHDs at baseline, mean (SE)**	8.3 (0.4)	8.6 (0.3)
**Number of monthly acute medications use days at baseline, mean (SE)**	4.7 (0.2)	5.3 (0.3)
**VAS score at baseline, mean (SE)**	6.9 (0.1)	6.7 (0.1)
**HIT-6 total score at baseline, mean (SE)**	67.6 (0.7)	67.4 (0.7)
**MSQoL RR score at baseline, mean (SE)**	45.4 (3.1)	46.9 (2.9)
**MSQoL RP score at baseline, mean (SE)**	58.8 (2.8)	57.9 (3.0)
**MSQoL EF score at baseline, mean (SE)**	65.1 (3.0)	64.2 (3.3)

SD, standard deviation; SE, standard error; MMDs, monthly migraine days; MHDs, monthly headache days; VAS, visual analog scale; HIT-6, 6-item Headache Impact Test; MSQ, Migraine-Specific Quality of Life Questionnaire; RR, Role Function-Restrictive; RP, Role Function-Preventive; EF, Emotional Function.

Due to rounding, the sum of the percentages of each part may not equal 100%.

[a]Previous use was counted by participants rather than the total number of preventive medications' classes in the same participant. Includes patients with previous preventive medication use, without treatment failure due to lack of efficacy or tolerability, and patients who failed the preventive medications.

[b]Failure due to lack of efficacy or poor tolerability.

© 2026 Zhang X et al. *JAMA Network Open*

eTable 5. Baseline characteristics in the per-protocol population

Characteristics	Real acupuncture group (n = 60)	Sham acupuncture group (n = 60)
**Age, mean (SD), years**	36.9 (10.2)	37.0 (9.8)
**Sex, No. (%)**		
Female	47 (79.7)	44 (80.0)
Male	12 (20.3)	11 (20.0)
**Current employment status, No. (%)**		
Employed	49 (83.1)	44 (80.0)
Student	8 (13.6)	8 (14.5)
Unemployed, retired	2 (3.4)	3 (5.5)
**Education, No. (%)**		
≤High school	5 (8.5)	3 (5.5)
College	44 (74.6)	43 (78.2)
Graduate degree	10 (16.9)	9 (16.4)
**Previous use of preventive medications, No. (%)**		
Naïve	35 (59.3)	33 (60.0)
Previous use[a]	24 (40.7)	22 (40.0)
Previous preventive medication failure[b]	21 (35.6)	17 (30.9)
**Disease duration, mean (SE), years**	16.0 (1.2)	16.4 (1.1)
**MMDs at baseline, mean (SE)**	6.6 (0.3)	6.6 (0.3)
**MHDs at baseline, mean (SE)**	8.5 (0.3)	8.6 (0.3)
**Number of monthly acute medications use days at baseline, mean (SE)**	4.7 (0.2)	5.3 (0.3)
**VAS score at baseline, mean (SE)**	6.9 (0.1)	6.7 (0.1)
**HIT-6 total score at baseline, mean (SE)**	67.6 (0.7)	67.2 (0.7)
**MSQoL RR score at baseline, mean (SE)**	45.2 (3.2)	48.5 (2.9)
**MSQoL RP score at baseline, mean (SE)**	59.1 (2.9)	58.3 (3.2)
**MSQoL EF score at baseline, mean (SE)**	65.0 (3.0)	66.5 (3.2)

SD, standard deviation; SE, standard error; MMDs, monthly migraine days; MHDs, monthly headache days; VAS, visual analog scale; HIT-6, 6-item Headache Impact Test; MSQ, Migraine-Specific Quality of Life Questionnaire; RR, Role Function-Restrictive; RP, Role Function-Preventive; EF, Emotional Function.

Due to rounding, the sum of the percentages of each part may not equal 100%.

[a]Previous use was counted by participants rather than the total number of preventive medications' classes in the same participant. Includes patients with previous preventive medication use, without treatment failure due to lack of efficacy or tolerability, and patients who failed the preventive medications.

[b]Failure due to lack of efficacy or poor tolerability.

© 2026 Zhang X et al. *JAMA Network Open*

eTable 6. Primary and secondary clinical efficacy outcomes in the per-protocol population during the treatment period

	Participants median (IQR)		Median difference (95% CI)	*P* value
	Real acupuncture group (n = 60)	Sham acupuncture group (n = 60)		
**Primary outcome**				
Change from baseline in MMDs during wks 1 to 4	-3.0 (-5.0, -2.0)	-3.0 (-4.0, -1.0)	-1.0, (-2.0, 0)	.03
**Secondary outcomes**				
≥50% Reduction in MMDs during wks 1 to 4, percentage (SE)	66.7 (6.1)	53.3 (6.5)	OR: 1.80, (0.8, 3.7)	.14
Change from baseline in MHDs during wks 1 to 4	-4.0 (-6.0, -2.5)	-3.0 (-4.0, -1.0)	-1.0, (-2.0, 0)	.01
Change from baseline in monthly acute medication use days during weeks 1 to 4	-3.0 (-4.0, -2.0)	-2.0 (-4.0, -1.0)	-1.0, (-1.0, 0)	.04
Change from baseline in VAS total score at wk 4[a]	-2.0 (-4.0, -1.0)	-1.0 (-2.0, -1.0)	-1.00, (-1.5, 0)	.01
Change from baseline in HIT-6 total score at wk 4, mean (SE)[b]	-8.3 (0.8)	-5.4 (0.9)	Mean defference: -2.9, (-5.4, -0.5)	.02
Change from baseline in MSQ RR at wk 4[c]	20.0 (11.6, 28.1)	8.6 (0, 21.4)	10.0, (4.8, 15.7)	< .001
Change from baseline in MSQ RP at wk 4[c]	15.0 (5.0, 25.0)	10.0 (0, 20.0)	5.0, (0, 10.0)	< .001
Change from baseline in MSQ EF at wk 4[c]	13.3 (6.7, 20.0)	0 (0, 16.7)	6.7, (0, 13.3)	.03

Abbreviations: MMD, monthly migraine day; SE, standard error; MHD, monthly headache day; VAS, visual analog scale; HIT-6, 6-item Headache Impact Test ;MSQ, Migraine-Specific Quality of Life Questionnaire; OR, odds ratio; RR, Role Function-Restrictive; RP, Role Function-Preventive; EF, Emotional Function.

[a]VAS score range: 0 (indicating no pain) to 10 (indicating severe pain).

[b]HIT-6 score range: 36 to 78, with the higher scores indicating severe headache effect.

[c]MSQ score range: 0 to 100, with the higher scores indicating superior quality of life.

© 2026 Zhang X et al. *JAMA Network Open*

eTable 7. Participants' satisfaction at week 4. Values are numbers (percentages) unless stated otherwise

At week 4			
	**Real acupuncture group (n = 60)**	**Sham acupuncture group (n = 60)**	**P value[a]**
5=extremely satisfied	28 (46.7)	22 (36.7)	
4=satisfied	30 (50.0)	22 (36.7)	
3=moderately satisfied	2 (3.3)	13 (21.7)	
2=dissatisfied	0 (0)	3 (5.0)	
1=extremely dissatisfied	0 (0)	0 (0)	
Responder (score of 4-5)	58 (96.7)	44 (73.3)	.01

Due to rounding, the sum of the percentages of each part may not equal 100%.

[a]Using χ^2 test.

© 2026 Zhang X et al. *JAMA Network Open*

eTable 8. Acupuncture Expectancy Scale Score at baseline. Values are numbers (percentages) unless stated otherwise

	Real acupuncture group (n = 60)	Sham acupuncture group (n = 60)
**Acupuncture expectation of improvement[a]**		
Ineffective	0 (0)	0 (0)
May be ineffective	2 (3.3)	4 (6.7)
Unclear	6 (10.0)	8 (13.3)
May be effective	27 (45.0)	30 (50.0)
Effective	25 (41.7)	18 (30.0)

Due to rounding, the sum of the percentages of each part may not equal 100%.

[a]The Fisher's exact test was used to compare the expectations of treatment's effect between the two groups (P = .52)

© 2026 Zhang X et al. *JAMA Network Open*

eTable 9. Compliance data at week 4. Values are numbers (percentages) unless stated otherwise

	Real acupuncture group (n = 60)	Sham acupuncture group (n = 60)
**The number of treatment sessions received, mean (SD)**	11.9 (0.9)	11.6 (1.3)
**Participants received at least 10 sessions of treatment (12 sessions in total, compliance rates ≥ 80%)[a]**	59 (98.3)	55 (91.7)

SD, standard deviation.

[a]There was no significant difference between groups in the compliance of acupuncture (P = .22).

© 2026 Zhang X et al. *JAMA Network Open*

eTable 10. Participant-Blinding Assessment Results. Values are numbers (percentages) unless stated otherwise

	Think in Real acupuncture group (n = 29)	Think in Sham acupuncture group (n = 9)	Did not know	P value[a]	Bang blinding index (95%CI)[b]
**At week 4[c]**					
**Real acupuncture group (n = 59)**	16 (27.1)	4 (6.8)	39 (66.1)	.85	0.6, [0.35, 0.85]
**Sham acupuncture group (n = 55)**	13 (23.6)	5 (9.0)	37 (67.3)		-0.4, [-0.59, -0.29]

Due to rounding, the sum of the percentages of each part may not equal 100%.

[a]Using χ^2 test.

[b]The Bang blinding index for each group represents the proportion of participants making a correct treatment guess beyond chance: 0 represents perfect blinding; a positive index indicates a correct guess, and a negative index indicates a guess in the opposite direction.

[c]Six participants (1 in RA group and 5 in SA group) did not complete the 4-week treatment due to various reasons (Details in eFigure 1).

© 2026 Zhang X et al. *JAMA Network Open*

eTable 11. Adverse events related to treatment

	Real acupuncture group (n = 60)	Sham acupuncture group (n = 60)
**Overall[a] , No. (%)**	5 (8.3)	5 (8.3)
**Serious adverse event, No. (%)**	0 (0)	0 (0)
**Adverse event, No. (%)**	5 (8.3)	5 (8.3)
Subcutaneous hematoma	2 (3.3)	3 (5.0)
Needling pain after treatment	1 (1.7)	1 (1.7)
Numbness	2 (3.3)	1 (1.7)

Adverse events were analyzed in all participants who received treatment. Adverse events were counted by type rather than frequency in the same participant. Adverse events with different types occurring in a single participant were defined as independent adverse events. An adverse event with multiple occurrences in a single participant was defined as 1 adverse event.

[a]The Fisher's exact test was used to analyze adverse events between the two groups (*P* = .77).

© 2026 Zhang X et al. *JAMA Network Open*

eReferences

1. Headache Classification Committee of the International Headache Society (IHS) The International Classification of Headache Disorders, 3rd edition. *Cephalalgia*. 2018;38(1)doi:10.1177/0333102417738202

2. Hawker GA, Mian S, Kendzerska T, French M. Measures of adult pain: Visual Analog Scale for Pain (VAS Pain), Numeric Rating Scale for Pain (NRS Pain), McGill Pain Questionnaire (MPQ), Short-Form McGill Pain Questionnaire (SF-MPQ), Chronic Pain Grade Scale (CPGS), Short Form-36 Bodily Pain Scale (SF-36 BPS), and Measure of Intermittent and Constant Osteoarthritis Pain (ICOAP). *Arthritis Care Res (Hoboken)*. 2011;63 Suppl 11:S240-S252. doi:10.1002/acr.20543

3. Houts CR, Wirth RJ, McGinley JS, et al. Content Validity of HIT-6 as a Measure of Headache Impact in People With Migraine: A Narrative Review. *Headache*. 2020;60(1):28-39. doi:10.1111/head.13701

4. Kosinski M, Bayliss MS, Bjorner JB, et al. A six-item short-form survey for measuring headache impact: the HIT-6. *Qual Life Res*. 2003;12(8):963-974.

5. Jhingran P, Davis SM, LaVange LM, Miller DW, Helms RW. MSQ: Migraine-Specific Quality-of-Life Questionnaire. Further investigation of the factor structure. *Pharmacoeconomics*. 1998;13(6):707-717.

6. Jhingran P, Osterhaus JT, Miller DW, Lee JT, Kirchdoerfer L. Development and validation of the Migraine-Specific Quality of Life Questionnaire. *Headache*. 1998;38(4):295-302.

7. Shen X, Finn ES, Scheinost D, et al. Using connectome-based predictive modeling to predict individual behavior from brain connectivity. *Nat Protoc*. 2017;12(3):506-518. doi:10.1038/nprot.2016.178

8. Finn ES, Shen X, Scheinost D, et al. Functional connectome fingerprinting: identifying individuals using patterns of brain connectivity. *Nat Neurosci*. 2015;18(11):1664-1671. doi:10.1038/nn.4135

9. Rosenberg MD, Finn ES, Scheinost D, et al. A neuromarker of sustained attention from whole-brain functional connectivity. *Nat Neurosci*. 2016;19(1):165-171. doi:10.1038/nn.4179

© 2026 Zhang X et al. *JAMA Network Open*
