# Supplementary material for: Acupuncture for Migraine Without Aura and Connection-Based Efficacy Prediction: A Randomized Clinical Trial
Source: JAMA Netw Open. 2026 Jan 27;9(1):e2555454. doi: 10.1001/jamanetworkopen.2025.55454 (PMC12848631; doi:10.1001/jamanetworkopen.2025.55454)
Supplement: Supplement 4. — Data Sharing Statement [file jamanetwopen-e2555454-s004.pdf]

Data Sharing Statement

Zhang. Acupuncture for Migraine Without Aura and Connection-Based Efficacy Prediction. *JAMA Netw Open*. Published January 27, 2026. doi:10.1001/jamanetworkopen.2025.55454

Data
Additional Information: Predictive effect of acupuncture on Wang's five acupoints and eight needles in brain network of migraine without aura https://www.chictr.org.cn/showproj.html?proj=65829 ChiCTR2100044251
Data available: Yes
Data types: Deidentified participant data
How to access data: lululalavictor1985@126.com
When available: With publication

Supporting Documents
Document types: None

Additional Information
Who can access the data: Researchers whose proposed use of the data has been approved
Types of analyses: For any purpose or for a specified purpose
Mechanisms of data availability: After approval of a proposal
